# A pilot study on searching for peri-nuclear NeuN-positive cells

**DOI:** 10.7717/peerj.8254

**Published:** 2020-01-07

**Authors:** Yun Yu, Meiyu Wu, Nan Zhang, Hua Yin, Bin Shu, Weigang Duan

**Affiliations:** 1 Department of Pharmacology, School of Basic Medicine, Kunming Medical University, Kunming, Yunnan, China; 2 Yunnan Provincial Key Laboratory of Molecular Biology for Sinomedicine, Faculty of Basic Medicine, Yunnan University of Traditional Chinese Medicine, Kunming, Yunnan, China; 3 Jiangsu Center for Safety Evaluation of Drugs, Jiangsu Provincial Institute of Materia Medica, Nanjing Tech University, Nanjing, Jiangsu, China

**Keywords:** High-throughput sequencing, Fluorescence microscopy, Immunohistochemistry, NeuN-positive cells, NeuN protein, Western blotting

## Abstract

The aim of this study was to find out neuron (-like) cells in peripheral organs by cell markers in rats. Adult male Sprague-Dawley rats were anaesthetized. Their organs including brain, heart, lung, liver, kidney, stomach, duodenum, and ileum were harvested. The mRNA and protein in these organs were extracted. RNA sequencing (RNA-Seq) was carried out, and NeuN, a “specific” marker for neuronal soma, was assayed with Western blotting. The sections of the aforementioned organs were obtained after a routine fixation (4% methanal)-dehydration (ethanol)-embedding (paraffin) process. NeuN in the sections and seven non-neuronal cell lines was analyzed by immunofluorescence (IF) or immunohistochemistry (IHC). Neuronal markers, such as Eno2, NeuN (Rbfox3), choline acetyltransferase (Chat), as well as tyrosine hydroxylase (Th), and neuronal-glial markers, e.g., glial fibrillary acidic protein (Gfap), S100b, 2′, 3′-cyclic nucleotide 3′-phosphodiesterase (Cnp), and other related markers, were positively expressed in all the organs at mRNA level. NeuN was further analyzed by Western blotting. The IF and IHC assays showed that NeuN-positive cells were distributed in all the peripheral tissues (mainly peri-nuclear NeuN-positive cells) though with different patterns from that in brain (nuclear NeuN-positive cells), and a NeuN-negative tissue could not be found. Especially, NeuN and Myl3 co-expressed in the cytoplasm of myocardial cells, suggesting that NeuN could possess other functions than neuronal differentiation. Also, the protein was positively expressed in seven non-neuronal cell lines. Our findings suggested that NeuN-positive cells exist widely, and without identification of its distribution pattern, the specificity of NeuN for neurons could be limited.

## Introduction

Neural cells include neuron, oligodendrocyte and astrocyte, which can be derived from neural stem cells (Sirerol-Piquer et al., 2019); among them, neuron is the most important one. The cell involves in treating biological signals including electrical and chemical signals, in which other cells’ functions can be perceived, controlled, or regulated via their dendrites and axons. Neurons are essential for multicellular organisms to harmonize cellular functions. In mammals, neurons are dominantly distributed in the central nerve system (CNS), involving brain and spinal cord. In addition, several neurons are distributed in the peripheral nerve system (PNS) (Chiu, Von Hehn & Woolf, 2012), though they were not proved to exist in all peripheral organs. Ganglions, e.g., sympathetic ganglion and parasympathetic ganglion, are places where peripheral neurons are gathered to treat signals. Studies have shown that there are numerous neurons in gastrointestinal walls, as well as in adrenal glands. In the walls, a number of neurons and their neurites form the submucosal plexus to regulate gastrointestinal secretion (Kermarrec et al., 2018), while some form the myenteric plexus to regulate gastrointestinal movements (Ozbek et al., 2018). However, there were no reports demonstrated the existence of peripheral neurons in other organs, such as kidney, liver, lung, and heart. Are neurons anywhere?

Mature neurons were believed to be permanent cells though they were able to be regenerated (Tomassy et al., 2010). A mature neuron would execute special cellular functions depending on its specific proteins (Tanapat, 2013). Neuroglial cells also express specific proteins. Some of the specific proteins expressed by neural cells can be used as their markers. A number of “accepted” cell markers are listed in Table 1. Among them, some markers are located at somas, some at dendrites, some at axons, and others at neuroglial cells. In particular, NeuN (Rbfox3) was widely accepted as the “specific” cell marker for neurons (Duan et al., 2016; Mullen, Buck & Smith, 1992). It was revealed that mouse Fox-1 (mFox-1) and another homologue, Fox-2, were both specifically expressed in neurons in addition to muscle and heart; besides, the over-expression of both Fox-1 and Fox-2 isoforms specifically activated splicing of neuronally regulated exons (Duan et al., 2016; Underwood et al., 2005). NeuN (Rbfox3) is a hexaribonucleotide binding protein 3 since Fox-3, like Fox-2 and Fox-1, binds the hexaribonucleotide UGCAUG, which is involved in the regulation of mRNA splicing (Duan et al., 2016; Underwood et al., 2005).

**Table 1:** Proteins selectively located in neural cells adapted from previous studies.

No.	Gene	Alias	Description	Location	Ref.
1	Eno2	NSE	Neuronal enolase *γ*	Soma of neuron, some oligodendroglia	Schmechel et al. (1978)
2	Rbfox3	NeuN	RNA binding protein fox-1 homolog 3	Soma of neuron	Mullen, Buck & Smith (1992)
3	Map2		Microtubule-associated protein 2	Map-2a, developed axon; Map-2b, all the axons; Map-2c, new born axon	Izant & McIntosh (1980)
4	Tubb3	Tuj1	Tubulin *β*3	Axon	Lee et al. (1990)
6	Dcx		Doublecortin	Protuberance of neurite	Francis et al. (1999)
7	Chat	ChAT	Choline O-acetyltransferase	Soma of cholinergic neuron	Perez et al. (2007)
7	Th	TH	Tyrosine hydroxylase	Soma of adrenergic neurons	Kubis et al. (2000)
8	Ncam1	PSA-NCAM	Neural cell adhesion molecule 1	Membrane of neuron	Gascon, Vutskits & Kiss (2007)
9	Ncam2	PSA-NCAM	Neural cell adhesion molecule 2	Membrane of neuron	
10	Neurod1	NeuroD	Neuronal differentiation 1	Cytoplasm and nucleus of neuron	Chae, Stein & Lee (2004)
11	Mapt	Tau	Microtubule-associated protein tau	Distal axon	Shin et al. (1991)
12	Calb1	Calbindin-D28k	Calbindin 1	Neural cells	Bastianelli (2003)
13	Calb2	Calretinin	Calbindin 2	Cytoplasma of neurons	Gulyas et al. (1992)
14	Nefh	NFP	Neurofilament, heavy polypeptide	Axon	Iwanaga, Takahashi & Fujita (1989)
15	Nefl	NFP	Neurofilament, light polypeptide	Axon	
16	Nefm	NFP	Neurofilament, medium polypeptide	Axon	
17	Gfap		Glial fibrillary acidic protein	Astrocyte, neural stem cells	Maniatis et al. (2019)
18	S100b		S100 calcium binding protein B	Astrocyte	Bianchi et al. (2007)
19	Vim		Vimentin	Astrocyte, neuron	Levin et al. (2009)
20	Cnp		3′-cyclic nucleotide 3′ phosphodiesterase	Oligodendrocyte, Schwann cell	Sprinkle (1989)

According to the theory of neurodevelopment, neural cells can migrate from one place to another during embryonic development. Early neural cells are resulted from neuroepithelial cells in the neural tube; some may migrate to the CNS, and others may migrate out to the PNS. As neural cells express specific proteins, they can be recognized by them. Therefore, the present study aimed to find out neurons or nueron-like cells in peripheral organs that were not believed to be involved.

## Experimental procedures

### Materials

Adult male Sprague-Dawley (SD) rats (age, 2 months; body weight, 180-220 g) were provided by Jianyang Dashuo Animal Science and Technology Co. Ltd. (Chengdu, China). Rats were maintained at 22 °C and humidity of 45–55% under natural light. This study was approved by the Animal Care and Use Committee of Jiangsu Provicial Institute of Materia Medica (Approved No. LL-20170830-01), Nanjing Tech University, Nanjing, China. Six cell lines were provided by Luoyu Biotechnology Co. Ltd. (Kunming, China). They are 293T (human renal epithelial cell), A549 (human lung cancer cell), BRL3A (rat heptocyte), Caco2 (human enterocyte), HL7702 (human heptocyte), PC12Adh (rat pheochromocytoma cell). These cell lines were identified according to their morphology. In addition, atrial muscle cells were achieved from primary culture.

Rabbit anti-NeuN (Rbfox3) monoclonal antibodies (BM4354 and ab177487), mouse anti-Myosin light chain 3 monoclonal antibody (Myl3) (ab680), mouse anti-β-actin monoclonal antibody, fluorescein isothiocyanate (FITC)-linked goat anti-rabbit IgG, Alexa Fluor® 647-linked donkey anti-rabbit IgG, Alexa Fluor® 647-linked goat anti-mouse IgG, and horseradish peroxidase (HRP)-linked rabbit anti-mouse IgG polyclonal antibody were purchased from Boster Bio-Engineering Co. Ltd. (Wuhan, China) and Abcam (Cambridge, UK). Besides, Hoechst 33342 and DAB (3,3-diaminobenzidine) staining kits were purchased from Sangon Biotech Co. Ltd. (Shanghai China). Enhanced chemoluminescence (ECL) detection kits were purchased from Pierce Biotechnology Inc. (Rockford, USA). NanoDrop ND-1000 spectrophotometer was manufactured by PeqLab (Erlangen, Germany). A fluorescence microscope was manufactured by Olympus Corp. (Tokyo, Japan).

### Animal treatments

In this study, 6 rats were normally treated for three days. Then, the animals were intraperitoneally anaesthetized with urethane (1.0 g/kg). Among them, three rats’ chests and abdomens were opened, and their organs, such as brain (frontal cortex), heart (left ventricle), liver, lung, kidney (left), stomach, duodenum, and the end ileum were harvested. The tunica and mesentery of the organs was removed clearly. All the organs were divided into two aliquots: one was used for mRNA sequencing, and the other was for Western blotting. Both aliquots were frozen at −80 °C for analysis or kept in icy water for immediate analysis.

Regarding the other three animals, when they were anaesthetized, their chests and abdomens were opened. Normal saline solution was injected into their left ventricle immediately. The perfusate was discharged via the right atrium by making a small hole with small scissors. After the normal saline solution (200 ml) was injected, 4% formaldehyde solution (200 ml) was followed. Then, the aforementioned organs were harvested, and fixed in 4% formaldehyde solution for immunofluorescence (IF) and immunohistochemistry (IHC) assays.

### High-throughput sequencing of mRNA

The aliquot organs for mRNA sequencing were frozen with liquid nitrogen and ground to powder. The total RNA in the powder was extracted and purified by TRIzol Plus RNA Purification kit (Invitrogen, Carlsbad, CA, USA). The quantity and quality of RNA were measured by the the NanoDrop ND-1000 spectrophotometer. RNA integrity was assessed by denaturing gel electrophoresis of RNA as previously described (Chen et al., 2017; Yin et al., 2015).

Double-stranded cDNA (ds-cDNA) was synthesized from the total RNA using a SuperScript ds-cDNA synthesis kit (Invitrogen, Carlsbad, USA) in the presence of 100 pmol/L oligo dT primers. The amplified cDNA was sequenced by Sangon Biotech Co. Ltd. (Shanghai, China). Expected value of fragments per kilobase of transcript sequence per million base pairs sequenced (FPKM) was used for normalization of expression level (Lin et al., 2017; Trapnell et al., 2010). The relative abundance was calculated by dividing FPKM of other organ by that of the brain. The analysis of the FPKM value of every gene among different organs was conducted by Sangon Biotech Co. Ltd. (Shanghai, China) as well.

### Western blotting

The alituot organs for Western blotting were homogenized in ice-cold, isotonic lysis buffer as previously described (Corson et al., 2011). Supernatant for the analysis was obtained by spinning the homogenate at 10,000 rpm at 4 °C for 5 min. The concentration of total protein in the supernatant was assayed by bicinchoninic acid (BCA) reagent (Boster Bio-Engineering Co. Ltd., Wuhan, China), and the concentration of different samples was adjusted to the same level as well.

Expression level of NeuN protein was detected by Western blotting, that was described previously (Chen et al., 2016; Chen et al., 2017; Hou et al., 2015). Briefly, total protein (20.0 µg) of samples was applied on to the sodium dodecyl sulfate-polyacrylamide gel electrophoresis (SDS-PAGE). The separated protein in SDS-PAGE was transferred to a polyvinylidene fluoride (PVDF) membrane by an electrical current at 10 V for 60 min in a semi-dry electrophoretic transfer cell. The PVDF membrane was blocked with 3% bovine serum albumin (BSA) at room temperature for 2 h; then, it was bathed in the primary antibody solution (dilution, 1:500) at 4 °C overnight. The membrane was rinsed with TST buffer (20 mmole/L Tris–HCl, pH 7.5, 0.05% Tween-20) for 10 min three times, and then bathed in the HRP-linked secondary antibody (dilution, 1:1,000) at room temperature for another 2 h. The membrane was again rinsed with TST buffer for 10 min three times, and analyzed by the ECL detection kit. The signal of ECL was recorded by a gel imager, and the band brightness was quantified by ImageJ 1.48v software.

### IF and IHC assays of tissue sections

To perform IF and IHC assays, the harvested organs were immersed in 4% paraformaldehyde solution until a routine histological operation was conducted. Paraffin-embedded sections (10 µm) of the organs were cut. The sections were affixed to glass slides, and blocked with 3% BSA. Then, the sections were bathed in the primary antibody solution (BM4354, 1:500) at 4 °C overnight. The sections were rinsed with TST buffer and bathed in the HRP- or FITC-linked secondary antibody (dilution, 1:1,000) at room temperature for another 2 h. Then, sections for IF assay were stained with Hoechst 33342 solution. When the green and blue fluorescence of the sections were excited by a high pressure mercury lamp, green (450 ± 10 nm) and blue (510 ± 10 nm) signals were filtered by a blue or green filter, and exposed to a CCD (charge coupled device) camera for 10 s. Images of the same field were recorded separately and merged together by the fluorescence microscopy. IF assay for the sections of heart, lung, liver and kidney was verified by staining with the rabbit anti-NeuN antibody (ab177487, from Abcam) and Alexa Fluor® 647-linked donkey anti-rabbit IgG (red). Especially, heart sections were further verified by double IF by staining with mouse anti Myl3 antibody and Alexa Fluor® 647-linked goat anti-mouse IgG (red). Total cells in 3 fields that contained 100 or more cells were analyzed, and cells emitting NeuN signals were counted as NeuN-positive cells. NeuN-positive cell rate was calculated by Formula (1).

(1) \begin{eqnarray*}\text{NeuN-positive} \text{cell} \text{rate}= \frac{{\text{Number}}_{\text{NeuN-positive} \text{cell}}}{{\text{Number}}_{\text{Total} \text{cell}}} \times 100\text{%}.\end{eqnarray*}

If the sections were stained with the HRP-linked secondary antibody, they were developed with the DAB staining kit, and images were visualized with the fluorescence microscope in a light mode.

### IF assays of cell lines

Routine methods of cell culture were curried on. In brief, 293T cells, A549 cells, BRL3A cells, HL7702 cells, and atrial muscle cells were cultured in Dulbecco’s modified Eagle medium (DMEM) with 2 mmol/L L-glutamine and 10% fetal bovine serum (FBS). Caco2 cells were cultured in DMEM with 2 mmol/L L-glutamine, 1 µmol/L sodium pyruvate, and 10% FBS. PC12 Adh cells were cultured in RPMI-1640 medium with 2 mmol/L L-glutamine and 10% FBS. The single cells were piped and cultured on to a coverslip in a 24-well plate. The coverslip was fixed with 4% formaldehyde and blocked with 3% BSA. Then, the coverslip was bathed in the primary antibody solution (dilution, 1:500) at 4 °C overnight. The coverslip was rinsed with TST buffer and bathed in the FITC-linked secondary antibody (dilution, 1:1,000) at room temperature for another 2 h. The coverslip was rinsed with TST buffer, and stained with Hoechst 33342 solution. When the green and blue fluorenscence of the coverslip were excited by the high pressure mercury lamp, green (450 ± 10 nm) and blue (510 ± 10 nm) signals were filtered by the blue or green filter, and exposed to the CCD camera for 10 s. Images of the same field were recorded separately and merged together by the fluorescence microscopy. Total cells in 3 fields that contained 100 or more cells were analyzed and cells emitting green fluorenscence were counted as NeuN-positive cells. NeuN-positive cell rate was calculated by formula (1).

### Statistical analysis

Data were expressed as mean ± standard deviation (SD). As for data accorded with the normal distribution, ANOVA (analysis of variance) was performed to compare means between groups; if there was a significance, post-hoc statistical tests (equal varance) or Tamhane’s tests (unequal varances) was used to compare the mean values between every two groups. As for data not accorded with the normal distribution, a non-parametric test of Mann Whiteney test (two-tailed) was used to compare the median values between every two groups. Statistical significance was accepted at *P* < 0.05.

**Table 2:** Genes encoding “specific” neural proteins were expressed at mRNA level in different organs in rats (FPKM, Mean ± SD, *n* = 3).

No.	Gene	Brain	Heart	Liver	Lung	Kidney	Stomach	Duodenum	Ileum
1	Eno2	348.06 ± 69.39	2.21 ± 1.26^*^	0.15 ± 0.06^*^	2.46 ± 0.18^*^	1.60 ± 0.32^*^	3.88 ± 0.59^*^	1.86 ± 0.84^*^	3.12 ± 0.81^*^
2	Rbfox3	130.77 ± 84.05	0.36 ± 0.14^*^	0.02 ± 0.04^*^	0.16 ± 0.07^*^	0.18 ± 0.06^*^	1.14 ± 1.06^*^	0.1 ± 0.09^*^	0.22 ± 0.08^*^
3	Map2	130.04 ± 31.14	0.34 ± 0.31^*^	0.28 ± 0.12^*^	7.16 ± 0.43^*^	2.18 ± 0.50^*^	1.09 ± 0.54^*^	0.56 ± 0.22^*^	0.34 ± 0.04^*^
4	Tubb3	348.30 ± 147.01	2.22 ± 1.72^*^	0.27 ± 0.09^*^	0.58 ± 0.15^*^	0.38 ± 0.31^*^	3.88 ± 1.22^*^	2.95 ± 0.89^*^	5.55 ± 0.67^*^
5	Dcx	4.28 ± 3.76	0.07 ± 0.11^*^	0.02 ± 0.01^*^	0.04 ± 0.02^*^	0.03 ± 0.04^*^	0.08 ± 0.03^*^	0.03 ± 0.01^*^	0.06 ± 0.01^*^
6	Chat	1.73 ± 2.15	0.07 ± 0.12^*^	0.00 ± 0.00^*^	0.06 ± 0.04^*^	0.00 ± 0.00^*^	0.06 ± 0.06^*^	0.29 ± 0.13	0.46 ± 0.10
7	Th	2.29 ± 0.73	0.03 ± 0.02^*^	0.00 ± 0.00^*^	0.03 ± 0.03^*^	0.02 ± 0.04^*^	0.04 ± 0.03^*^	0.04 ± 0.08^*^	0.06 ± 0.07^*^
8	Ncam1	56.09 ± 10.67	6.46 ± 7.61^*^	0.17 ± 0.03^*^	1.47 ± 0.34^*^	2.11 ± 0.19^*^	4.29 ± 0.88^*^	1.25 ± 0.30^*^	2.23 ± 0.15^*^
9	Ncam2	7.64 ± 2.62	0.07 ± 0.09^*^	0.03 ± 0.03^*^	0.01 ± 0.02^*^	0.00 ± 0.00^*^	0.35 ± 0.11^*^	0.21 ± 0.07^*^	0.28 ± 0.09^*^
10	Neurod1	77.49 ± 127.37	0.03 ± 0.02^*^	0.04 ± 0.08^*^	0.01 ± 0.02^*^	0.02 ± 0.04^*^	0.83 ± 0.58^*^	0.67 ± 0.18^*^	0.81 ± 0.26^*^
11	Mapt	151.78 ± 30.35	22.03 ± 3.02^*^	1.09 ± 0.71^*^	43.32 ± 2.97^*^	50.64 ± 3.48^*^	1.18 ± 0.68^*^	2.09 ± 0.58^*^	2.75 ± 0.43^*^
12	Calb1	204.52 ± 216.24	0.04 ± 0.07^*^	0.00 ± 0.00^*^	0.12 ± 0.05^*^	262.13 ± 51.19	1.70 ± 1.50^*^	0.45 ± 0.22^*^	0.80 ± 0.09^*^
13	Calb2	153.77 ± 192.94	0.05 ± 0.05^*^	0.00 ± 0.00^*^	0.05 ± 0.06^*^	0.00 ± 0.00^*^	0.56 ± 0.26^*^	3.65 ± 1.31^*^	9.35 ± 0.14^*^
14	Nefh	55.52 ± 26.91	0.06 ± 0.09^*^	0.01 ± 0.01^*^	0.06 ± 0.04^*^	0.22 ± 0.14^*^	0.31 ± 0.18^*^	0.29 ± 0.18^*^	0.32 ± 0.06^*^
15	Nefl	189.20 ± 58.28	0.60 ± 0.93^*^	0.01 ± 0.02^*^	0.10 ± 0.09^*^	0.10 ± 0.10^*^	1.58 ± 0.52^*^	1.75 ± 0.48^*^	3.46 ± 0.22^*^
16	Nefm	92.46 ± 33.55	0.27 ± 0.45^*^	0.05 ± 0.02^*^	0.02 ± 0.02^*^	2.58 ± 0.96^*^	0.46 ± 0.03^*^	0.62 ± 0.26^*^	1.29 ± 0.09^*^
17	Gfap	215.36 ± 39.62	0.35 ± 0.33^*^	0.67 ± 0.14^*^	0.3 ± 0.12^*^	0.04 ± 0.03^*^	0.53 ± 0.28^*^	1.12 ± 0.57^*^	1.41 ± 0.41^*^
18	S100b	1255.51 ± 181.26	5.24 ± 6.75^*^	1.14 ± 1.10^*^	5.58 ± 2.5^*^	13.11 ± 2.74^*^	16.62 ± 9.34^*^	3.02 ± 1.03^*^	16.37 ± 19.01^*^
19	Vim	79.93 ± 9.48	278.03 ± 90.37^**^	28.35 ± 7.67^*^	736.49 ± 34.17^**^	66.70 ± 4.50^*^	149.18 ± 59.49^**^	62.07 ± 9.82	232.08 ± 92.37^**^
20	Cnp	396.16 ± 50.56	15.30 ± 6.95^*^	5.22 ± 0.26^*^	43.70 ± 14.03^*^	4.61 ± 0.40^*^	9.83 ± 0.95^*^	11.04 ± 5.47^*^	16.36 ± 2.14^*^

**Notes.**

* *p* < 0.05, significantly lower than that in brain.

** *P* < 0.05, significantly higher than that in brain.

Mann Whiteney test (two-tailed); The location of the proteins can be seen in Table 1.

## Results

### “Neural markers” expressed in different organs

**Figure 1:**
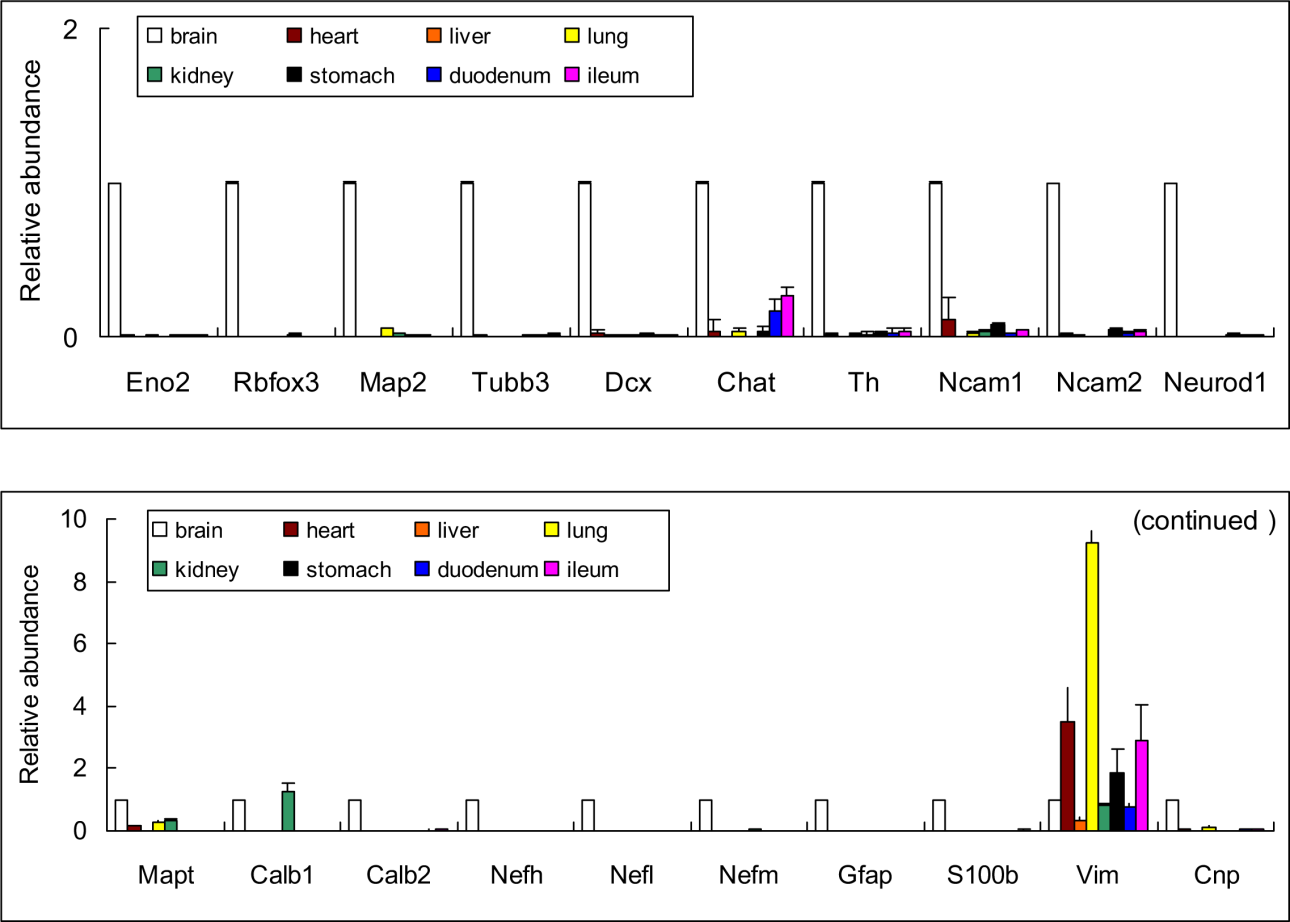
Relative abundance of genes encoded “specific” neural proteins expressed in different organs in rats (Mean ± SD, *n* = 3). Relative abundance = (other organ)/brain.

Neural cells express a number of specific proteins, some of which were typically used as markers (Table 1). We found that, neuronal markers, such as Eno2 (NSE, neuronal enolase *γ*), Rbfox3 (NeuN), Chat (ChAT, choline O-acetyltransferase), and Th (tyrosine hydroxylase), in addition to neuroglial markers, including Gfap (glial fibrillary acidic protein), S100b (S100 calcium binding protein B), Cnp (3′-cyclic nucleotide 3′ phosphodiesterase), and other related markers were positively expressed in all the organs at mRNA level (Table 2, Relative abundance was showed in Fig. 1). The results also showed that genes encoding the “neural markers” were highly expressed in the brain. The majority of the “neural markers” in gastrointestinal walls were expressed relatively at a high level, while the level was significantly lower than that in the brain. Our results were consistent with the previously reported findings (Kermarrec et al., 2018; Ozbek et al., 2018). Regarding other organs (e.g., heart, liver, lung and kidney), no studies have demonstrated that there were neurons in those organs, while the “neural markers” were detectable in the present study (Table 2). However, as shown in Table 2 and Fig. 1, Calb1 (calbindin 1) and Vim (vimentin) were not relatively selectively expressed in the rats’ CNS.

### NeuN expressed at protein level in different organs assayed by Western blotting

**Figure 2:**
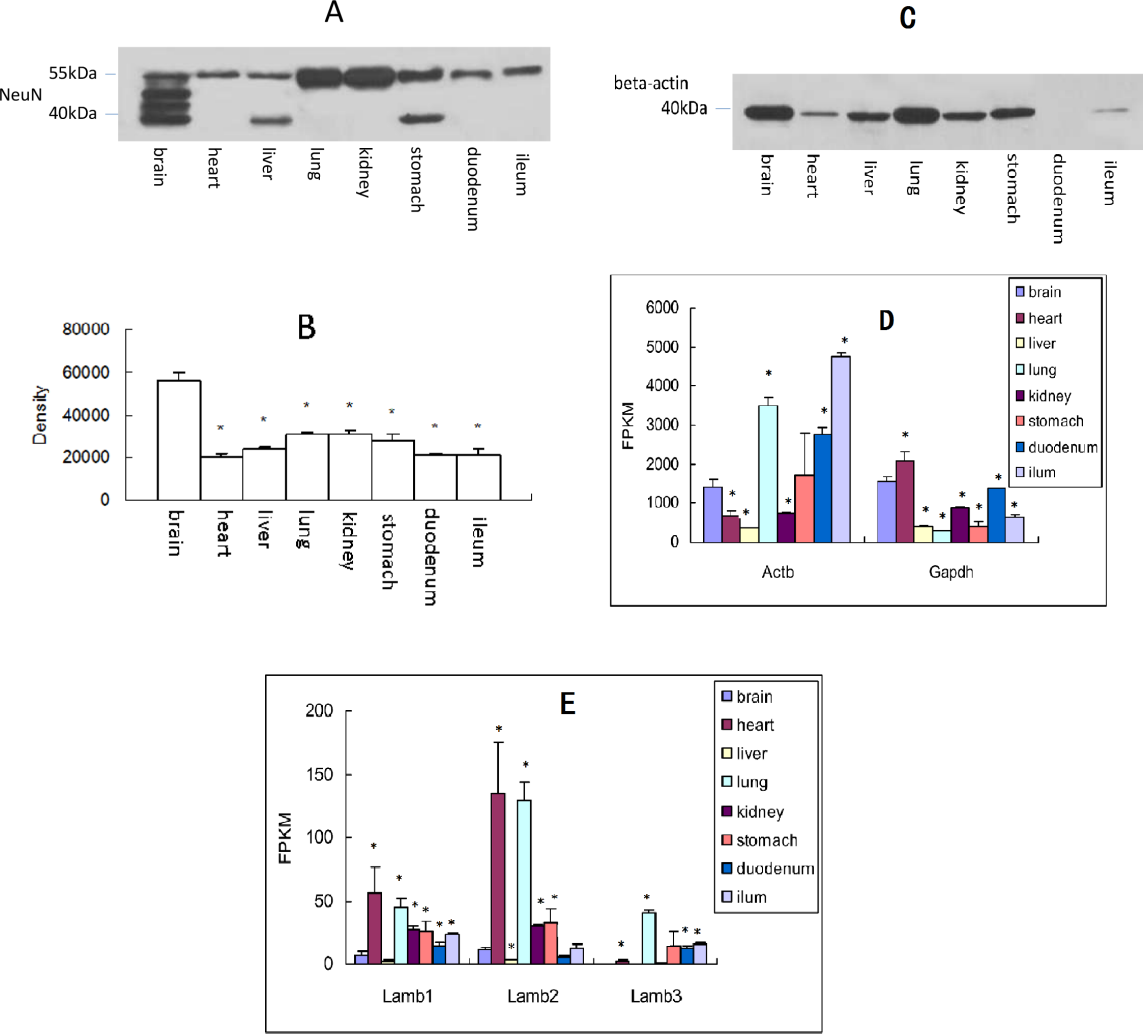
NeuN (Rbfox3) was expressed in different organs at protein level. (A) Western blot analysis; (B) semi-quantitative analysis of the results of Western blotting (Mean ± SD, *n* = 3); (C) *β*-actin expressed in different organs at protein level, * *P* < 0.05 vs. brain, ANOVA; (D) *β*-actin and Gapdh expressed in different organs at mRNA level. * *P* < 0.05 vs. brain, ANOVA; (E) Lambs expressed in different organs at mRNA level. FPKM = value of fragments per kilobase of transcript sequence per million base pairs sequenced, *, *P* < 0.05, Mann Whiteney test (two-tailed).

Among the above-mentioned genes, Rbfox3 (NeuN) has been one of the widely accepted markers for neuronal soma (Duan et al., 2016; Mullen, Buck & Smith, 1992). Western blot analysis verified the achieved results at mRNA level (Table 2). As expected, Figs. 2A and 2B showed that NeuN was existed in the eight organs, and the expression level was associated with its mRNA level (Table 2). It should be noted that, the molecular weight of NeuN is about 45 kD, and there were two or more bands in the lanes of brain, liver, and stomach (Figs. 2A and 2B), the reason of which may be associated with its tight binding to DNAs or RNAs (Mullen, Buck & Smith, 1992). The results also demonstrated that the abundance of NeuN at protein level in the organs have extra bands is higher than other organs. As internal controls, β-actin (Figs. 2C and 2D) and GAPDH (Fig. 2D) (for plasmosin) and Lamb (Fig. 2E, for nucleoprotein) were used widely in Western blot. However, their expression was different from organ to organ either at mRNA level (Figs. 2D and 2E) or at protein level (Fig. 2C).

**Figure 3:**
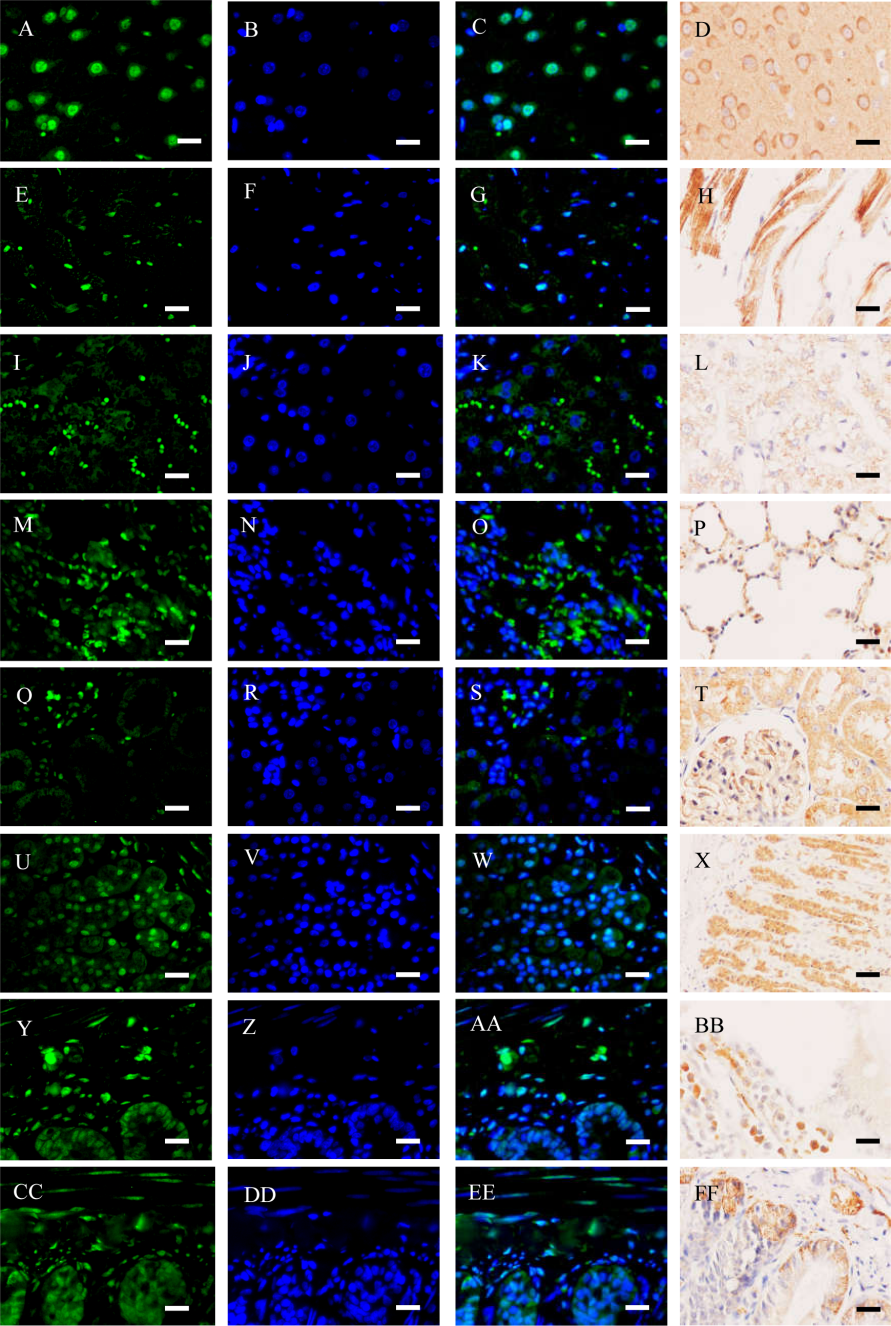
Immunofluorescence (IF) and immunohistochemistry (IHC) assays of different organs (bar = 20 µm). (A–D) Brain; (E–H) heart; (I–L), liver; (M–P) lung; (Q–T) kidney; (U–X) stomach; (Y–BB) duodenum; (CC–FF) ileum. As for IF, green fluorescence was stained to locate NeuN (BM4354, Boster) by FITC-linked secondary antibody, and blue fluorescence was stained to locate nucleus by Hoechst 33342. As for IHC, NeuN was stained in brown.

**Figure 4:**
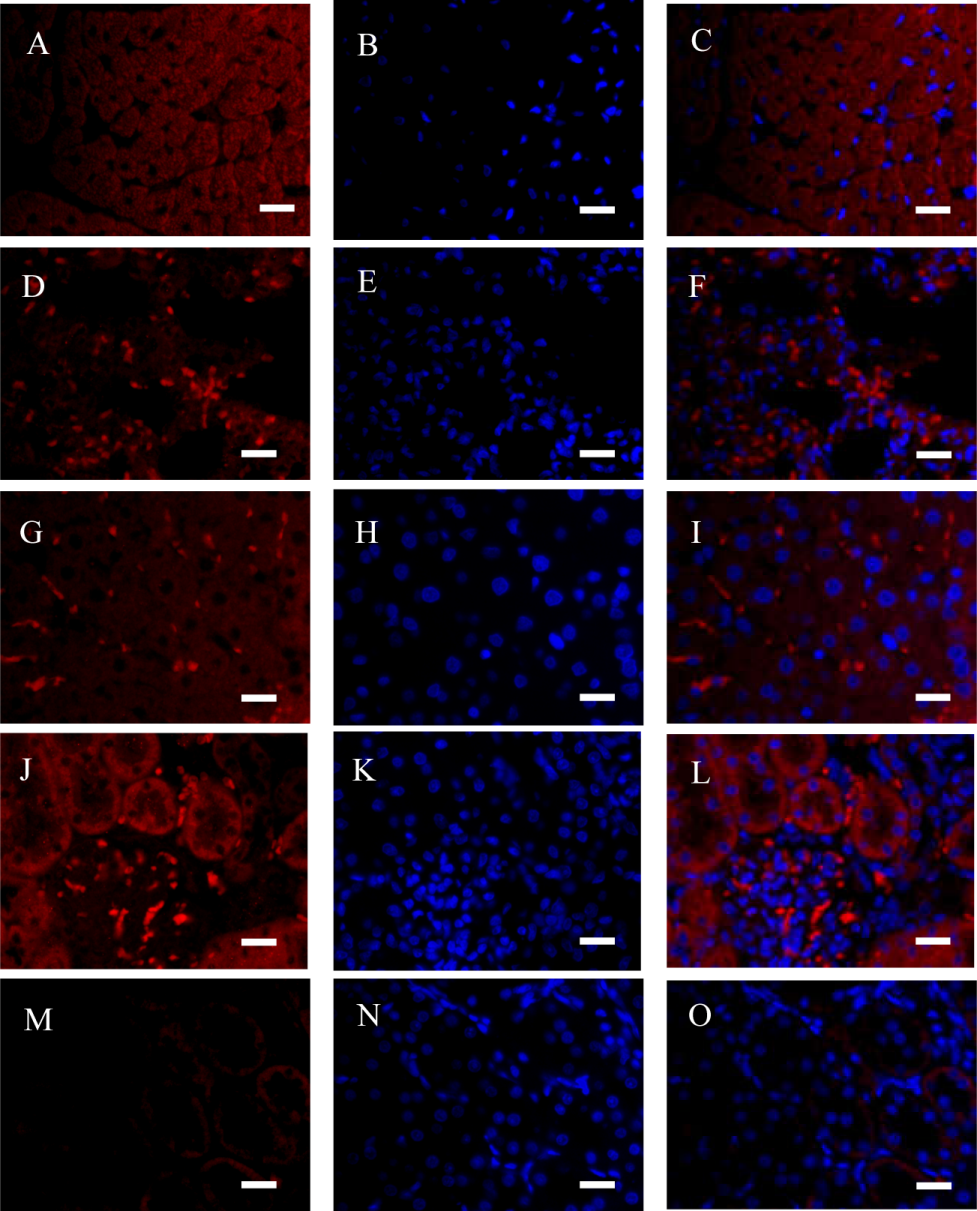
Immunofluorescence (IF) assays verified in four organs (bar = 20 µm). (A–C) Heart; (D–F) lung; (G–I) liver; (J–L) Kidney; (M–O) kidney (Negative control). Red fluorescence was stained to locate NeuN by another rabbit anti-NeuN monoclonal antibody (ab177487, Abcam), and blue fluorescence was stained to locate nucleus by Hoechst 33342. Negative control was only stained with Alexa Fluor^®^ 647-linked secondary antibody and Hoechst 33342.

**Figure 5:**
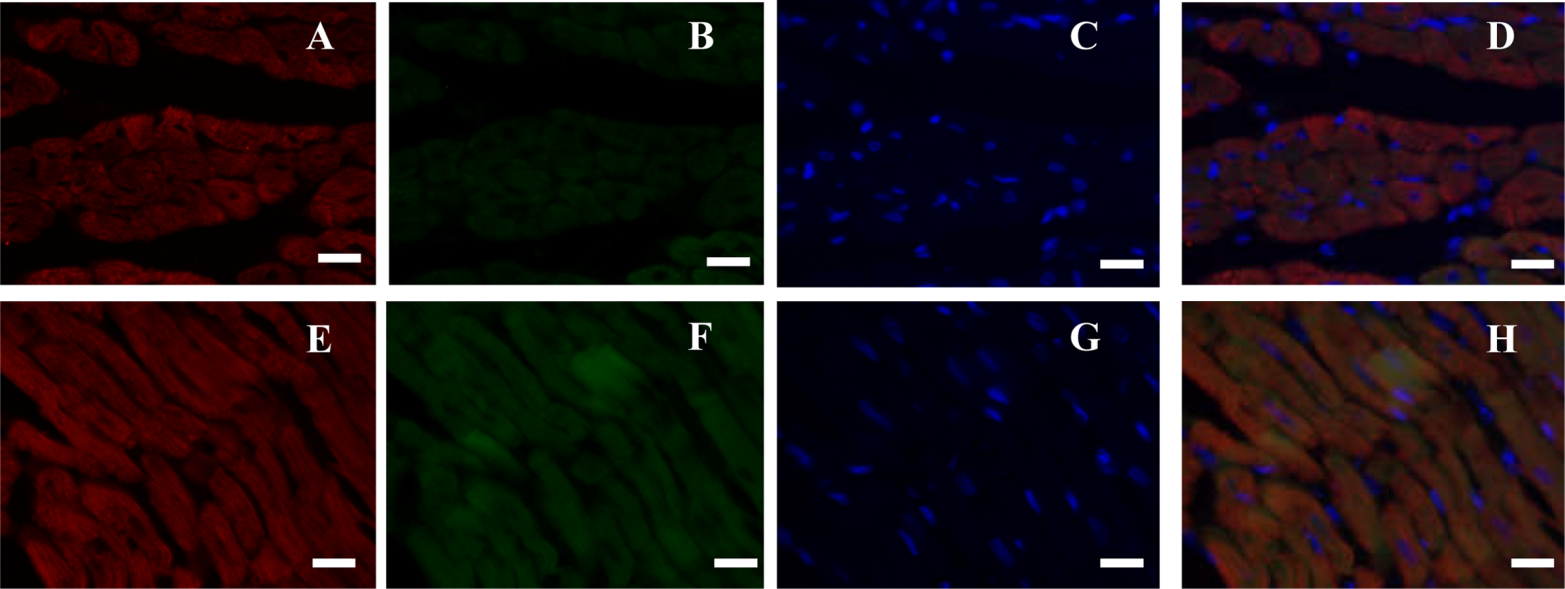
Double immunofluorescence (IF) analysis of rat heart (bar = 20 µm). (A–D) Cardiac muscle fibers in transection; (E–H) cardiac muscle fibers in longitudinal section. Red fluorescence was stained to locate Myl3 by Alexa Fluor^®^ 647-linked secondary antibody, green fluorescence was stained to locate NeuN by FITC-linked secondary antibody, and blue fluorescence was stained to locate nucleus by Hoechst 33342.

**Figure 6:**
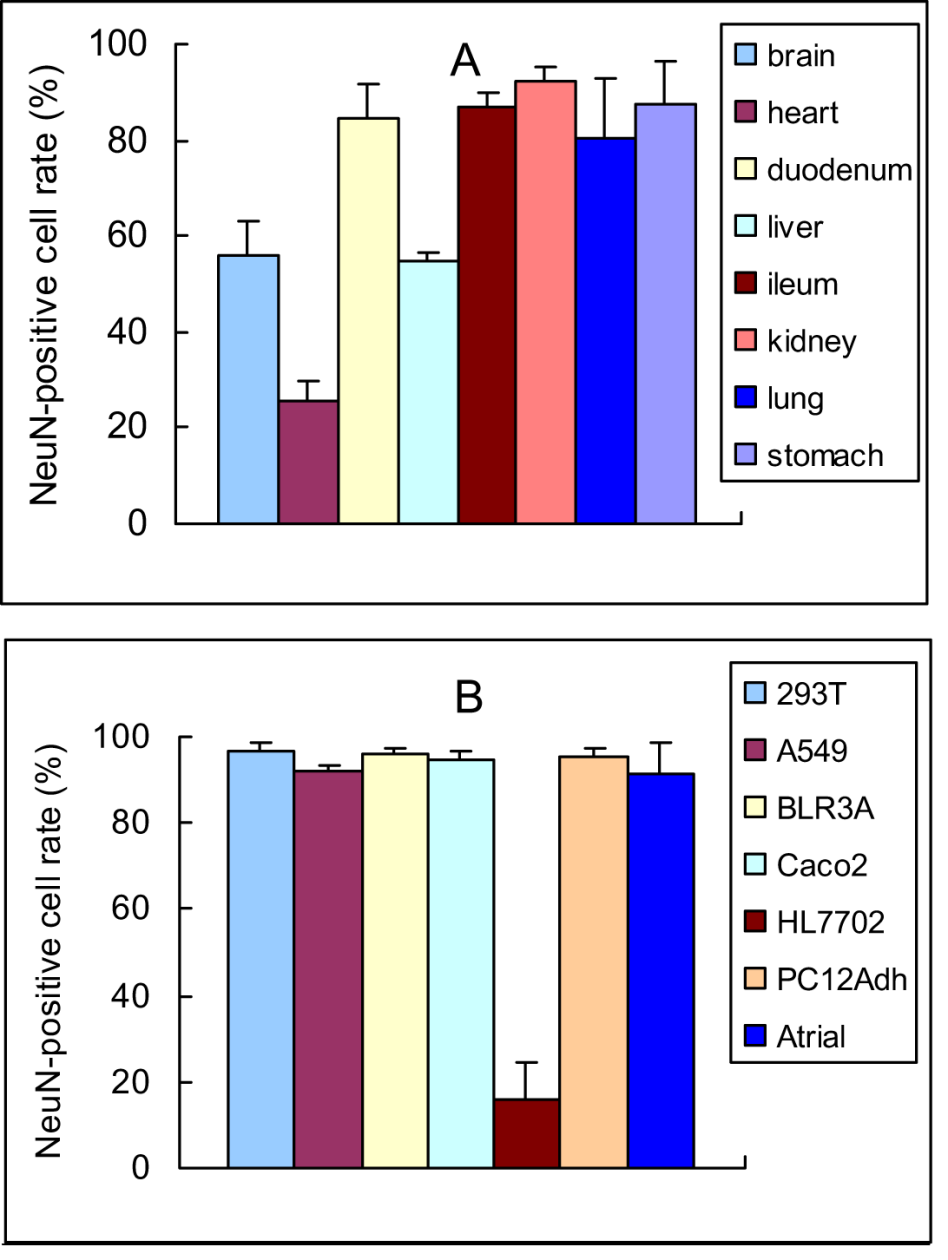
Quantitative analysis of NeuN-positive cells in rat organs and cultured cells (mean ± SD, *n* = 3). Photos can be seen in Fig. 3 (rat organs) and Fig. 7 (cultured cells). (A) NeuN-positive cell rate in rat organs; (B) NeuN-positive cell rate in cultured cells.

**Figure 7:**
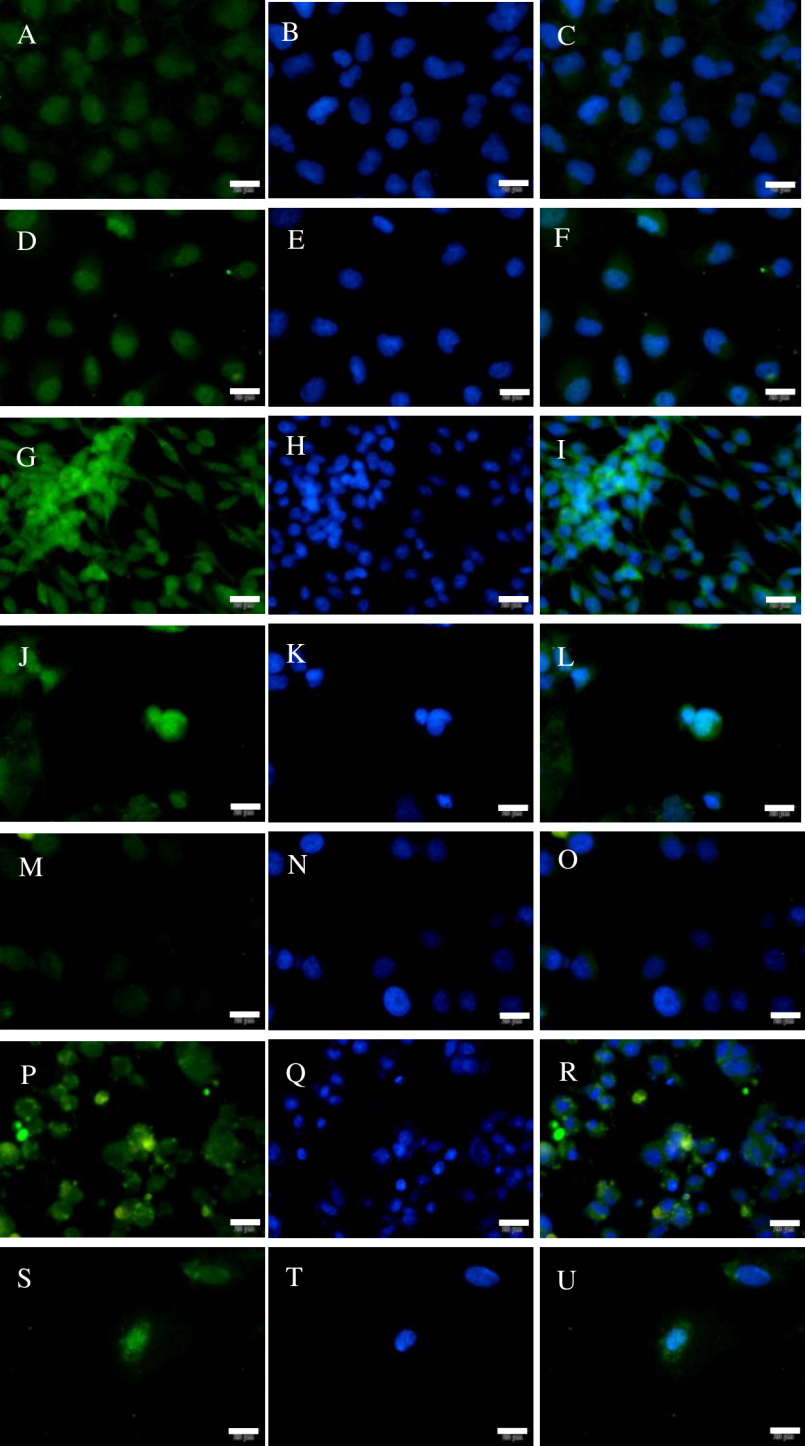
Immunofluorescence (IF) analysis of different cell lines (bar = 20 µm). (A–C) 293T cell; (D–F) A549 cell; (G–I) BRL3A cell; (J–L) Caco2 cell; (M–O) HL7702 cell; (P–R) PC12Adh cell; (S–U) atrial muscle cell. Green fluorescence was stained to locate NeuN by FITC-linked secondary antibody, and blue fluorescence was stained to locate nucleus by Hoechst 33342.

### NeuN expressed at protein level in different organs assayed by IF and IHC

NeuN in the organ sections was detected by IF and IHC assays (Fig. 3). Typically, NeuN is located in or near to cell’s nucleus. Brain contains several neurons, where NeuN can be positively stained around or in the nucleus (Figs. 3A–3D). However, NeuN can also be found in heart, liver, lung, kidney, and gastrointestinal walls. Regarding the latter seven organs, NeuN was sometimes located in cytoplasma or in nucleus, which were different from brain to some extent. For instance, NeuN was favorably found in the edge of muscle bundle in heart, in the hepatic lobules, in the walls of pulmonary alveoli, in glomeruli and the walls of tiny tubules, and under the mucosa and in muscular layers (Figs. 3E–3FF). Consideration of four organs concerned seldom having NeuN-positive reports, IF assay for the sections of heart, lung, liver and kidney was verified with another anti-NeuN antibody (ab177487, from Abcam), and similar results were obtained (Fig. 4). Especially, NeuN and Myl3 (a selective marker for myocardial cells) (Kim et al., 2016) coexpressed at cytoplasm in myocardial cells, though NeuN expressed at a lower level (Fig. 5). Though the NeuN-positive cell rates in several organs are higher than that in brain, their distribution patterns of NeuN are different. However, in accordance with the pattern of brain, the NeuN-positive cell rates in other organs could be much lower.

The NeuN-positive cell rate of brain (Fig. 6A) was similar to that of previous study (Mullen, Buck & Smith, 1992). To our surprise, however, the NeuN-positive cell rates of other organs were higher than that of the brain. Different from the brain, only a small portion of NeuN was located in or around the nucleus in other organ sections, and a good portion of the protein was located in the place far from the nucleus, suggesting the protein was able to transport to other place. To our surprise, we did not find a NeuN-negative tissue in rats.

### NeuN expressed at protein level in different cell lines assayed by IF

Here, NeuN was detected by IF assay in 7 cell lines (Fig. 7). Unexpectedly, all the cell lines expressed NeuN though HL7702 cell (Figs. 7M–7O) expressed at a lower level, and the protein was located in or near to cell’s nucleus as that in neurons (Figs. 3A–3D). Among the 7 cell lines, all of them expressed the protein (Fig. 6B) though they are believed to be non-neuronal cells.

## Discussion

The CNS plays a pivotal role in regulating body’s complex functions. The cells resided in the neural tube may develop neurons in brain and spinal cord, while a small portion of the cells may migrate outside to develop ganglions. However, in addition to the peripheral ganglions, gastrointestinal plexuses and adrenal glands, it should be elucidated whether there are other dispersed neural cells, especially neurons, apart from their fibers, in peripheral organs.

To date, several scholars have found a variety of markers for neural cells. Based on these markers (Table 1), neural cells, including neurons and neurogliocytes can be specifically spotted. According to the results presented in Table 2, almost all the markers for neural cells, including neurons and neurogliocytes, were positively detected in peripheral organs (e.g., heart, liver, lung, and kidney). Though nerve fibers are able to extend anywhere in the aforementioned organs, there was no reported evidence that these organs contained neural somas, even NeuN-positive cells.

Based on the results from rat organs (Table 2), “specific” markers for neurons and neurogliocytes were detectable in all the peripheral organs concerned. Since neurons are the most important cells in the neural system, we paid more attentions to them than to neurogliocytes. It is believed that, if there are neurons in a place in body, neurogliocytes would exist in the same place. NeuN is a well-known cell marker for neuronal soma (Duan et al., 2016; Mullen, Buck & Smith, 1992). In the present study, this marker was positively detected at both mRNA level and protein level in the above-mentioned organs and non-neuronal cell lines, making a question, whether neural cells or NeuN-positive cells exist anywhere?

As there were no reports concentrated on peripheral neurons in heart, lung, liver and kidney, and because we saw no morphological signs of neurite in the present study, we cannot conclude that NeuN-positive cells in the aforementioned organs were neurons. Cells with similar morphology or expressed similar specific antigen(s) to neuron can be recognized as a neuron-like cell. Neuron-like cells could be derived from neural cells or other differentiated cells. However, the cells typically play a role of transitional cell in neural development and differentiation, and the majority of them were identified in the CNS (Niu et al., 2018) or in differentiated cell lines (Rezaei et al., 2018). For instance, differentiated PC12 cells and other neuron-derived cells were recognized as neuron-like cells (Vainshtein et al., 2015). Because there were no identifiable dendrites and axons in the neuron-like cells, the NeuN-positive cells identified in the present study could be only another type of them. Consequently, NeuN-positive cells, rather than neurons, were distributed in heart, liver, lung, and kidney.

In particular, the distribution pattern of NeuN in peripheral cells was different from that of in the CNS (brain), for a good portion of the protein distributed in the place much far away from nucleus, suggesting that the protein in the peripheral cells were generated in the place around nucleus and transported far away. Therefore, it is possible that, NeuN from some peripheral organs positively detected by Western blotting (Figs. 2A–2B) could be caused by neural terminals. However, considering the results of cell markers expressed at mRNA level (Table 2), some NeuN-positive results could be derived from soma, because protein translation happens in ribosomes that exist in the place near to nucleus.

However, considering NeuN expressed in all the organs studied (Fig. 3) and non-neuronal cells (Fig. 7), NeuN seems to be a constitutive protein and the neuron-like cells identified by NeuN should be accepted with a pinch of salt. NeuN contains RNA recognition motif (RRM), by which the protein binds to RNA molecules and regulate mRNA splicing (Duan et al., 2016; Underwood et al., 2005). Becuase RRM domains are one of the most common stuctures in the human genome and are found in numerous proteins which bind RNA molecules (Underwood et al., 2005), there could be no specific reason for NeuN only involving in neuron development. In other words, RNA splicing is a common phenomenon in living cells, and cells could need the protein to live well, especially in some tumor cells like non-small-cell lung cancer (NSCLC) (Langenfeld et al., 2013).

Therefore, there could be at least two types of NeuN-positive cells. One is the nuclear NeuN-positive cell, and the other is the peri-nuclear NeuN-positive cell. The nuclear NeuN-positive cell is with its NeuN distributing in or around nucleus, mainly existed in the CNS and gastrointestinal walls; and the peri-nuclear NeuN-positive cell is with its most NeuN distributing in cytoplasm, existed in other peripheral organs. Considering NeuN expressed in non-neuronal cells, the protein could possess functions other than neuronal differentiation.

## Conclusion

NeuN-positive cells exist widely. Without identification of its distribution pattern, the specificity of NeuN for neurons could be limited. The effects of NeuN on cells, and the function of “NeuN-positive” cells need to be further assessed.

##  Supplemental Information

10.7717/peerj.8254/supp-1 Figure S1 Raw data for Fig. 1 Relative abundance was calculated based on original FPKM values. Values in Row 1-23 are original FPKM. Values in Row 26–48 are relative abundance calculated by Organ/Brain. Values in Row 51-71 are means (left) and SD (right) of every concerned genes. There are also to figures made based on the data of Row 51–71.

10.7717/peerj.8254/supp-2 Figure S2 Raw data for Fig. 2 Original photos of Western blotting for NeuN (Raw data for Figure 2A. pdf). Semi-quantitative analysis of original Figure 2A (Raw data for Figure 2B.xls). Original photos of Western blotting for beta-actin (Raw data for Figure 2C.pdf). *β*-actin and Gapdh expressed in different organs at mRNA level (Raw data for Figure 2D.xls). Lambs expressed in different organs at mRNA level (Raw data for Figure 2E.xls)

10.7717/peerj.8254/supp-3 Figure S3 Raw data for Fig. 3 32 photos comprising Figure 3.

10.7717/peerj.8254/supp-4 Figure S4 Raw data for Fig. 4 15 photos comprising Figure 4.

10.7717/peerj.8254/supp-5 Figure S5 Raw data for Figure 5 8 photos comprising Figure 5.

10.7717/peerj.8254/supp-6 Figure S6 Raw data for Fig. 6 Quantitative analysis of IF of Figure 3: the number of NeuN-positive cells (green) and total cells (blue), and the relative NeuN-positive cell rate was calculated.

10.7717/peerj.8254/supp-7 Figure S7 Raw data for Fig. 7 21 photos comprising Figure 7.

10.7717/peerj.8254/supp-8 Supplemental Information 8 Raw data for Table 2 The genes’ original FPKM in eight organs, means of every gene in organs, SD of every gene in organs, P-values vs brain from analysis results and the documentation of the full steps of analysis.

## Additional Information and Declarations

### Data Availability

The following information was supplied regarding data availability: The raw measurements are available in the Supplemental Files.
