# Supplementary figures and images for: A pilot study on searching for peri-nuclear NeuN-positive cells
Source: PeerJ. 2020 Jan 7;8:e8254. doi: 10.7717/peerj.8254 (PMC6953339; doi:10.7717/peerj.8254)

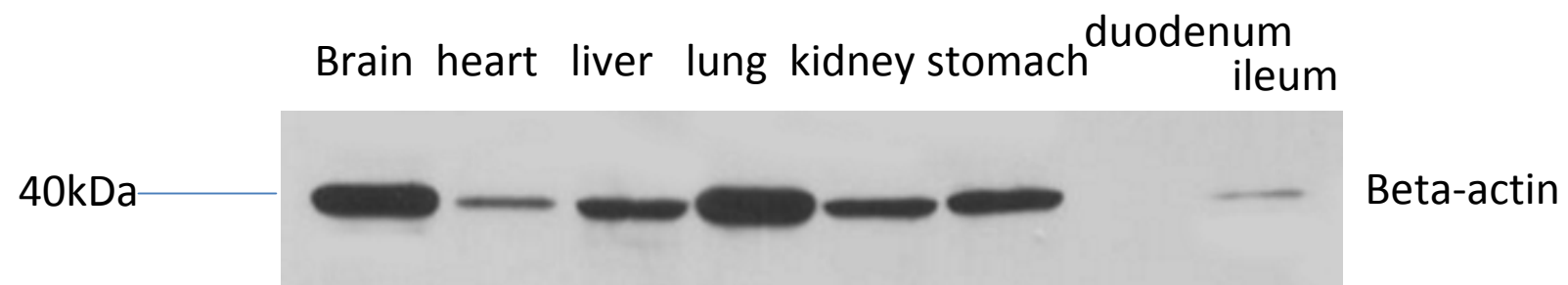

Supplement: Figure S2 — Original photos of Western blotting for NeuN (Raw data for Figure 2A. pdf). Semi-quantitative analysis of original Figure 2A (Raw data for Figure 2B.xls). Original photos of Western blotting for beta-actin (Raw data for Figure 2C.pdf). β-actin and Gapdh expressed in different organs at mRNA level (Raw data for Figure 2D.xls). Lambs expressed in different organs at mRNA level (Raw data for Figure 2E.xls) [file peerj-08-8254-s002.zip › Raw data Figure 2C.pdf]

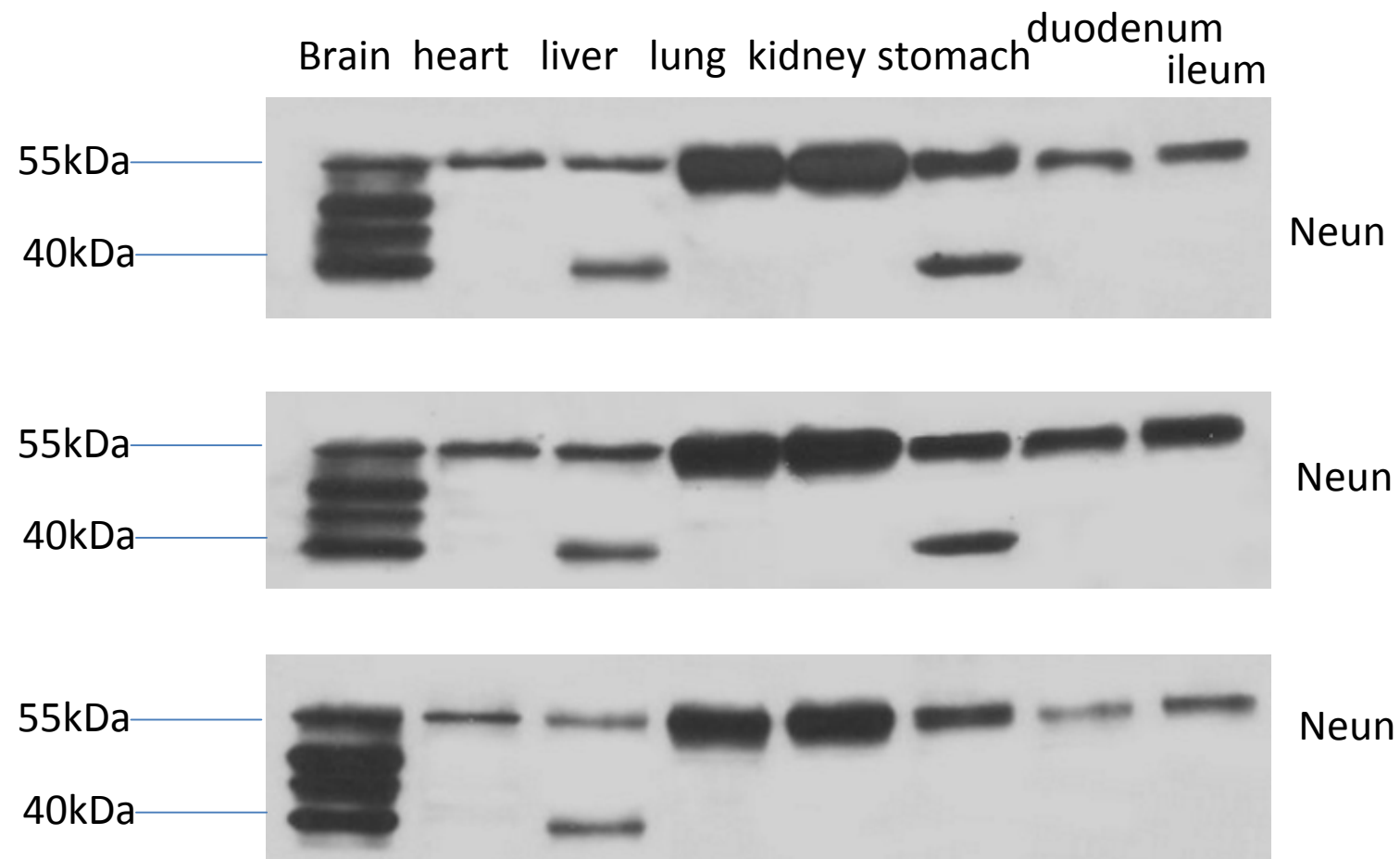

Supplement: Figure S2 — Original photos of Western blotting for NeuN (Raw data for Figure 2A. pdf). Semi-quantitative analysis of original Figure 2A (Raw data for Figure 2B.xls). Original photos of Western blotting for beta-actin (Raw data for Figure 2C.pdf). β-actin and Gapdh expressed in different organs at mRNA level (Raw data for Figure 2D.xls). Lambs expressed in different organs at mRNA level (Raw data for Figure 2E.xls) [file peerj-08-8254-s002.zip › Raw data for Figure 2A.pdf]

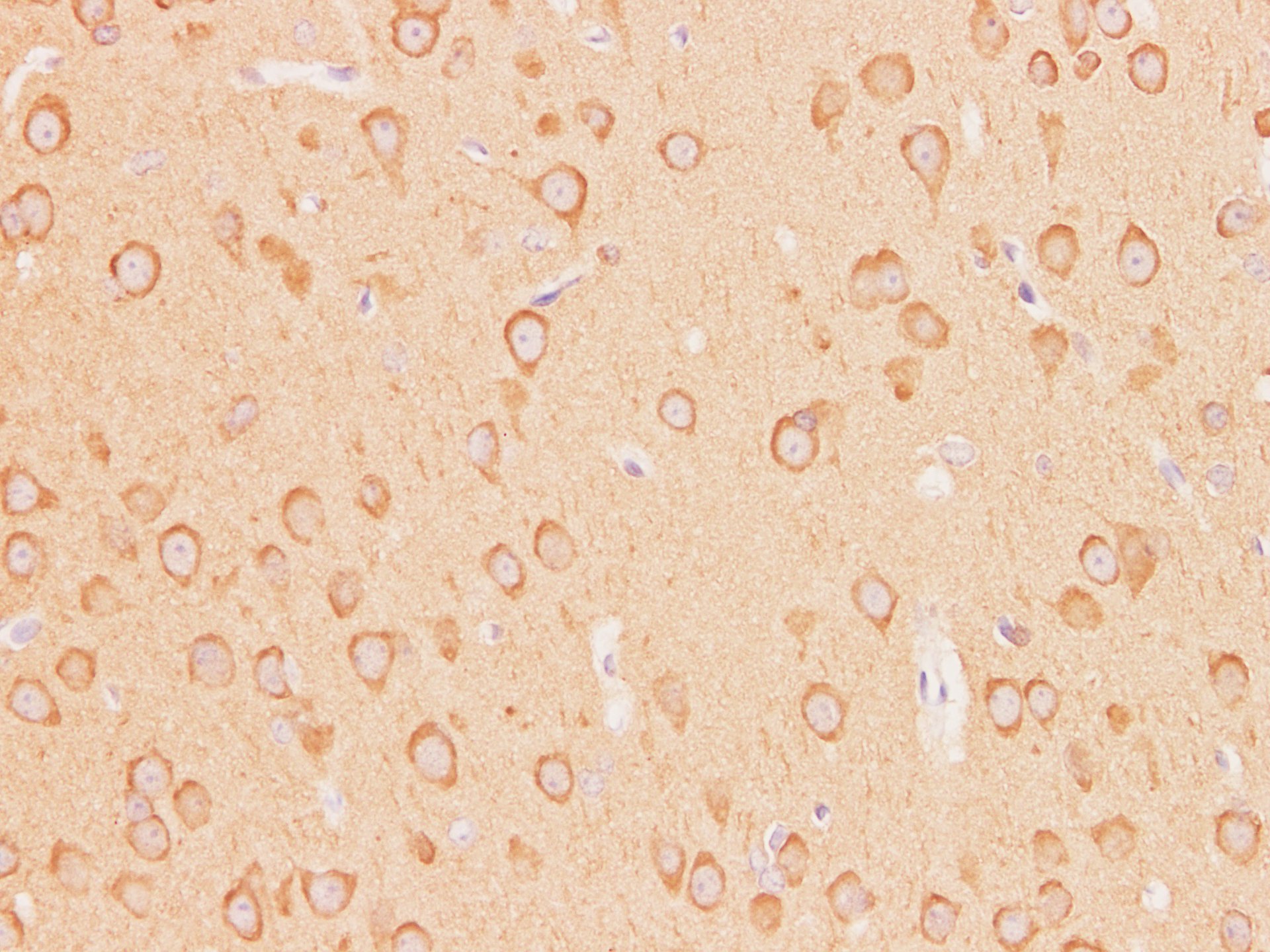

Supplement: Figure S3 — 32 photos comprising Figure 3. [file peerj-08-8254-s003.zip › Fig 3d.jpg]

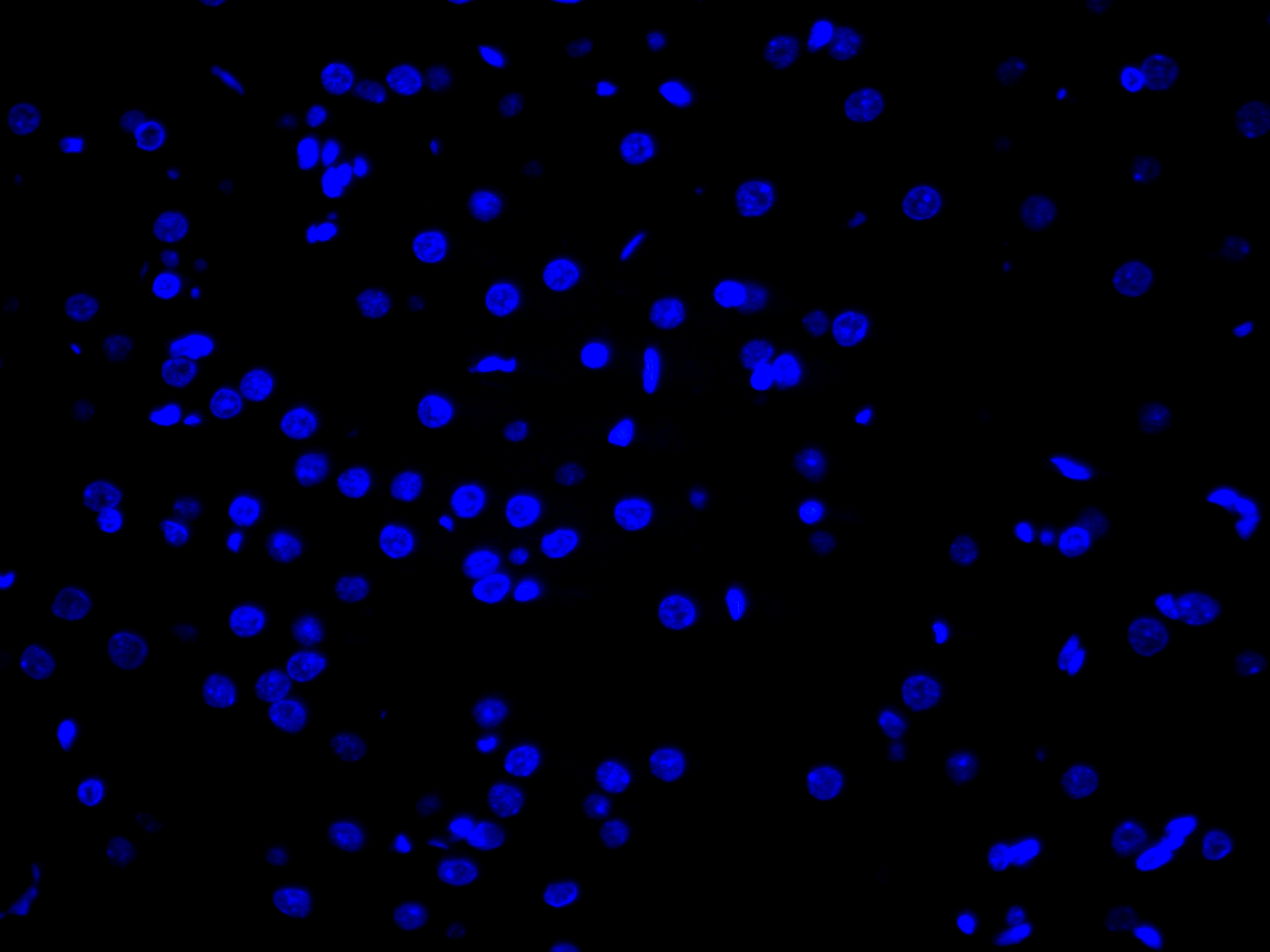

Supplement: Figure S3 — 32 photos comprising Figure 3. [file peerj-08-8254-s003.zip › Fig 3b.png]

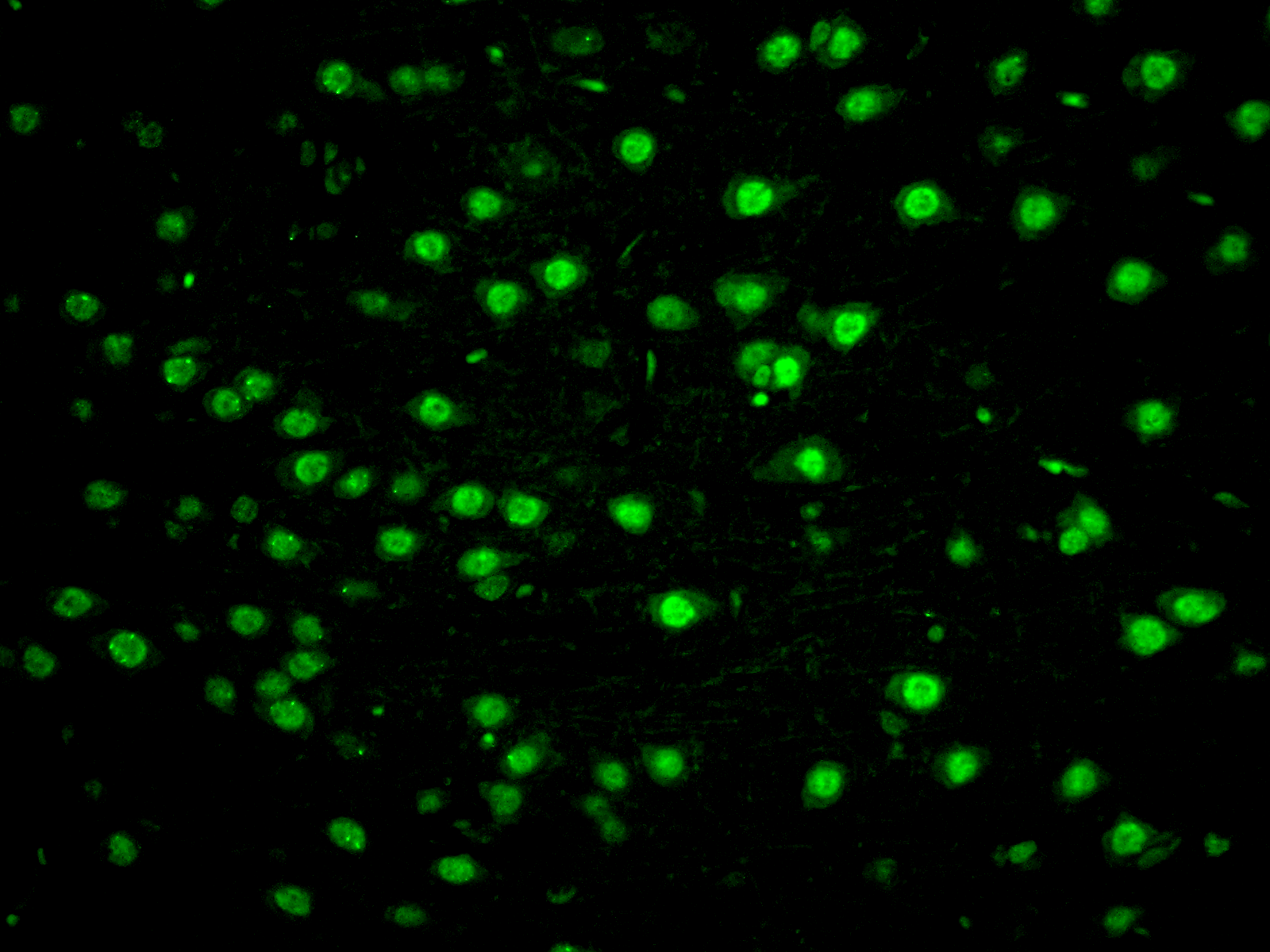

Supplement: Figure S3 — 32 photos comprising Figure 3. [file peerj-08-8254-s003.zip › Fig 3a.png]

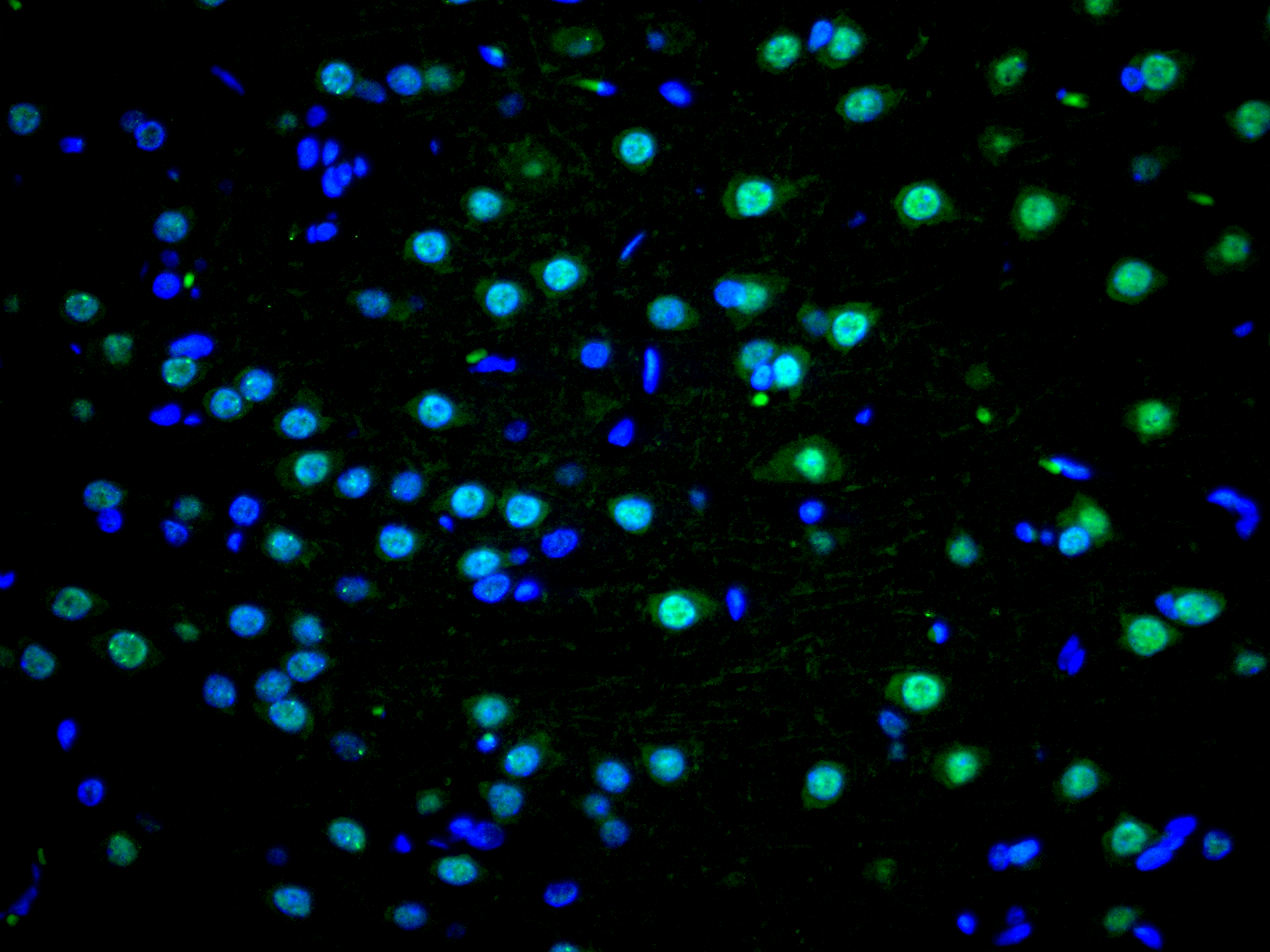

Supplement: Figure S3 — 32 photos comprising Figure 3. [file peerj-08-8254-s003.zip › Fig 3c.png]

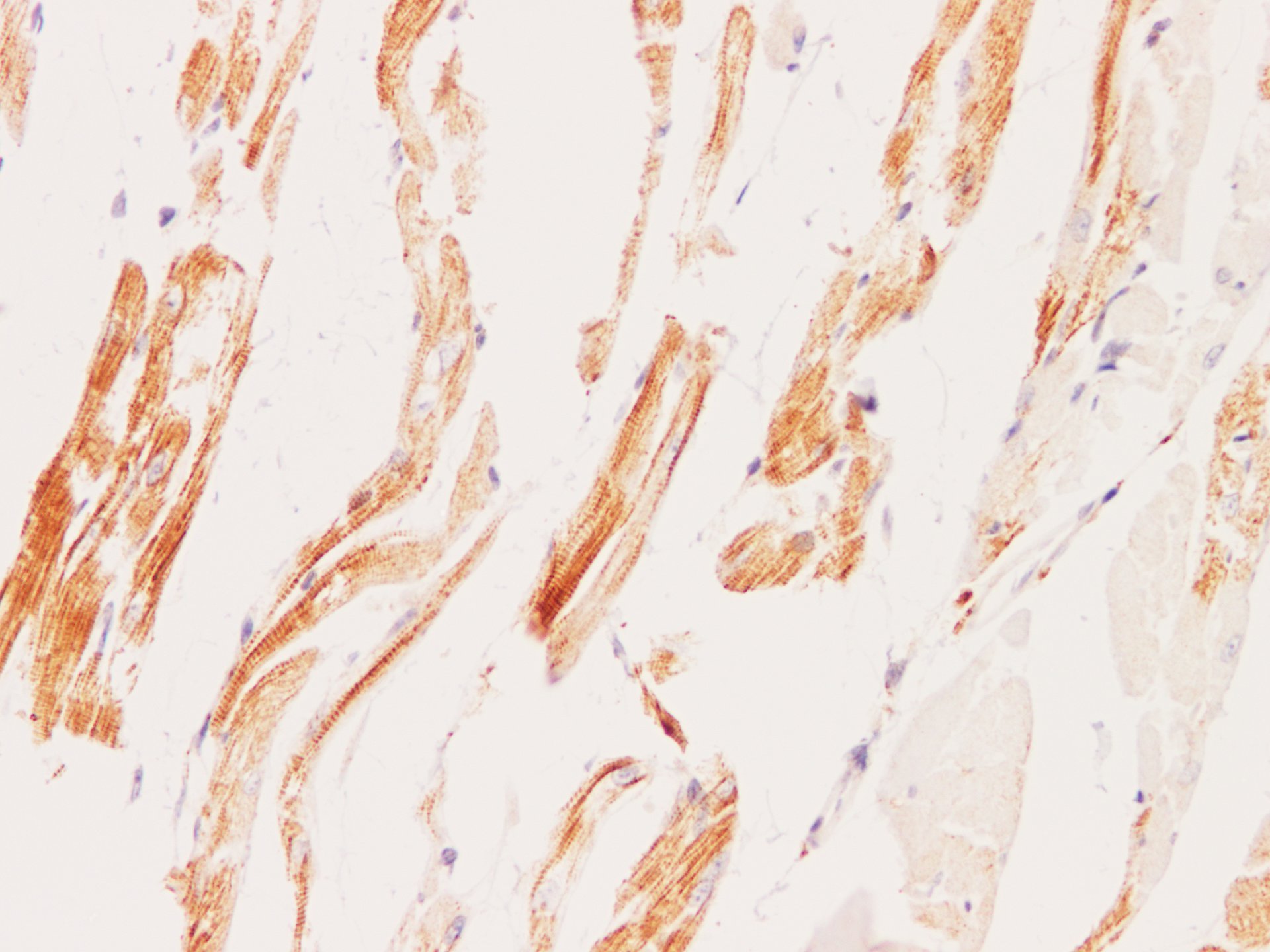

Supplement: Figure S3 — 32 photos comprising Figure 3. [file peerj-08-8254-s003.zip › Fig 3h.jpg]

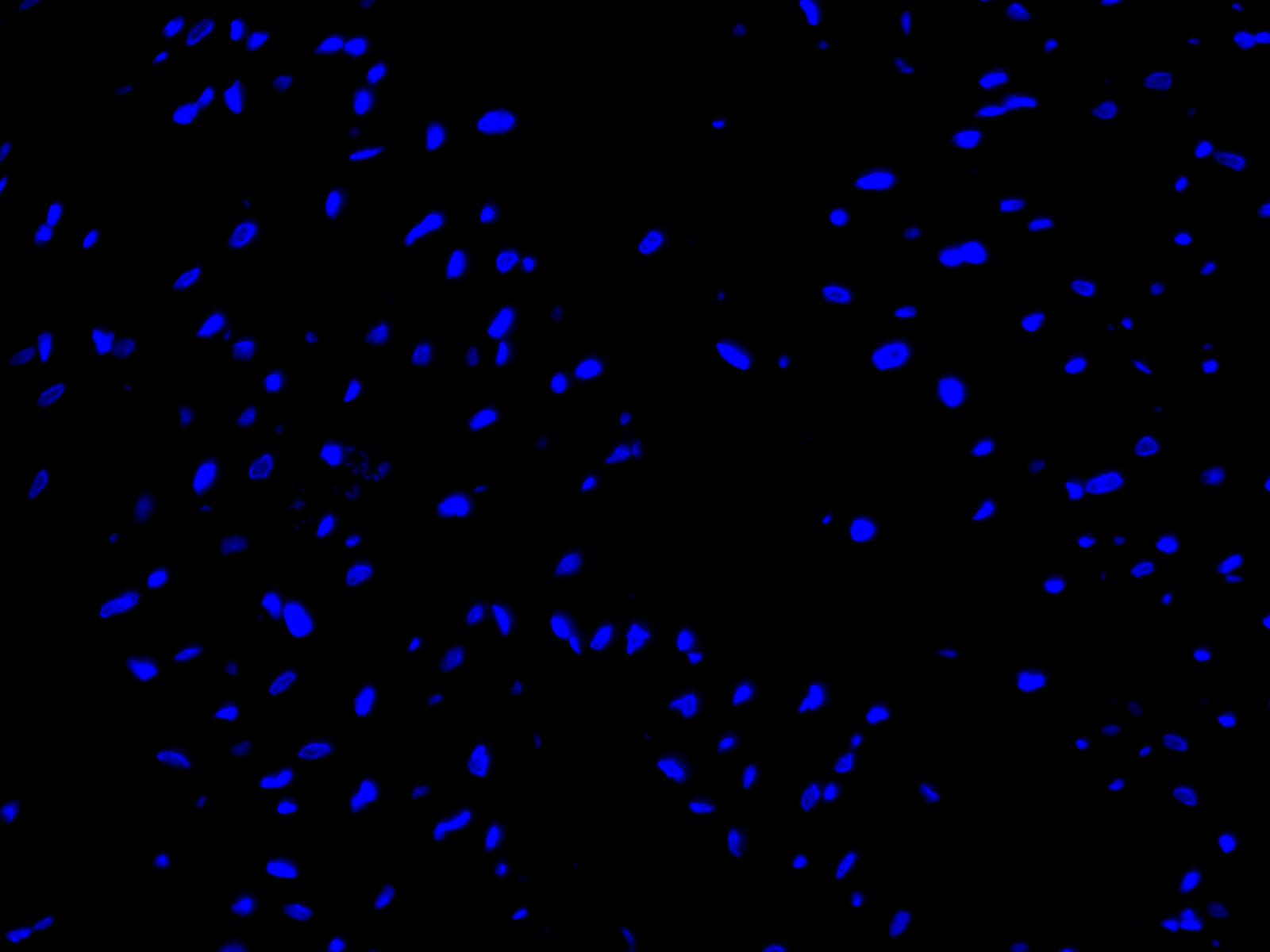

Supplement: Figure S3 — 32 photos comprising Figure 3. [file peerj-08-8254-s003.zip › Fig 3f.png]

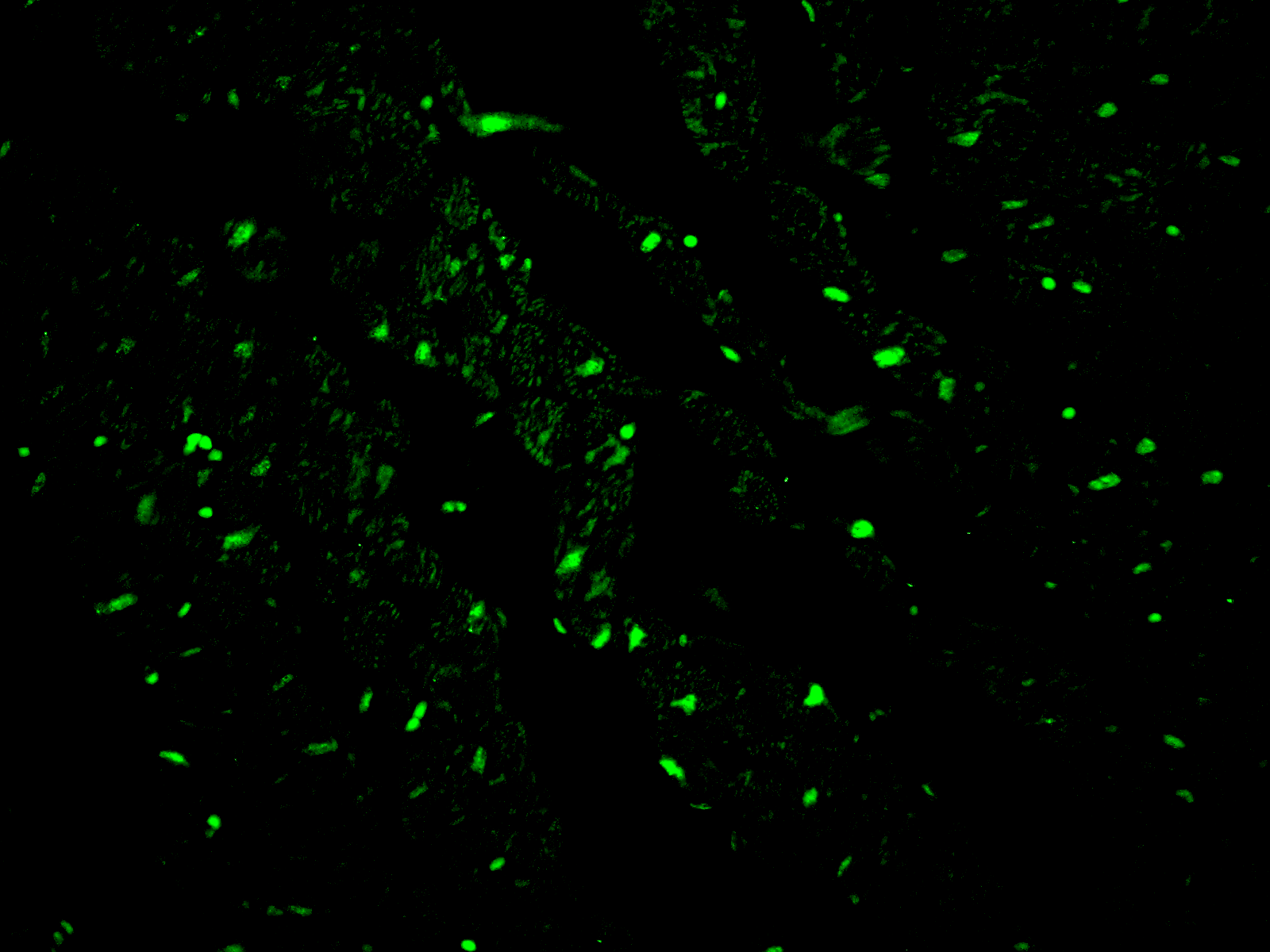

Supplement: Figure S3 — 32 photos comprising Figure 3. [file peerj-08-8254-s003.zip › Fig 3e.png]

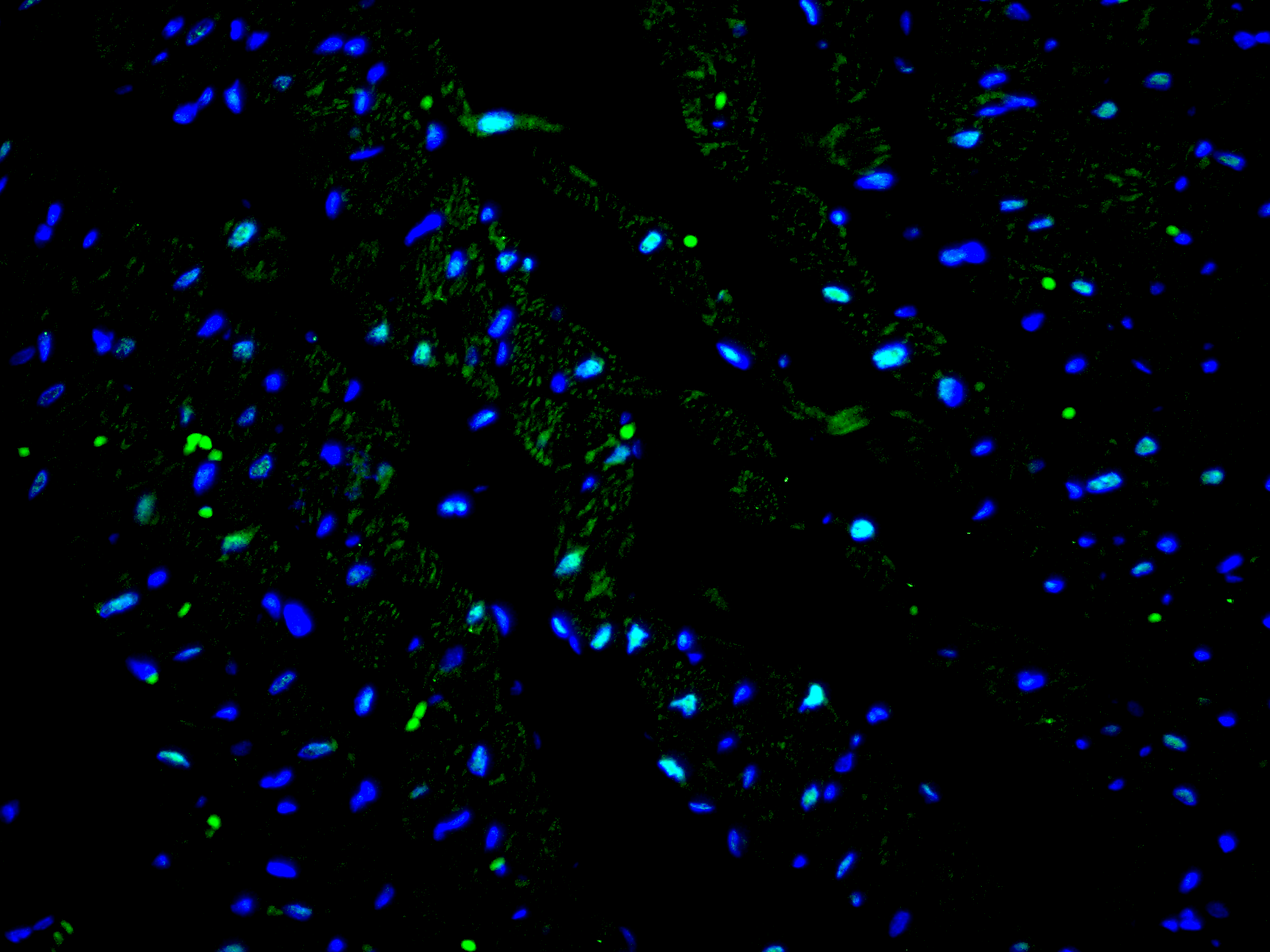

Supplement: Figure S3 — 32 photos comprising Figure 3. [file peerj-08-8254-s003.zip › Fig 3g.png]

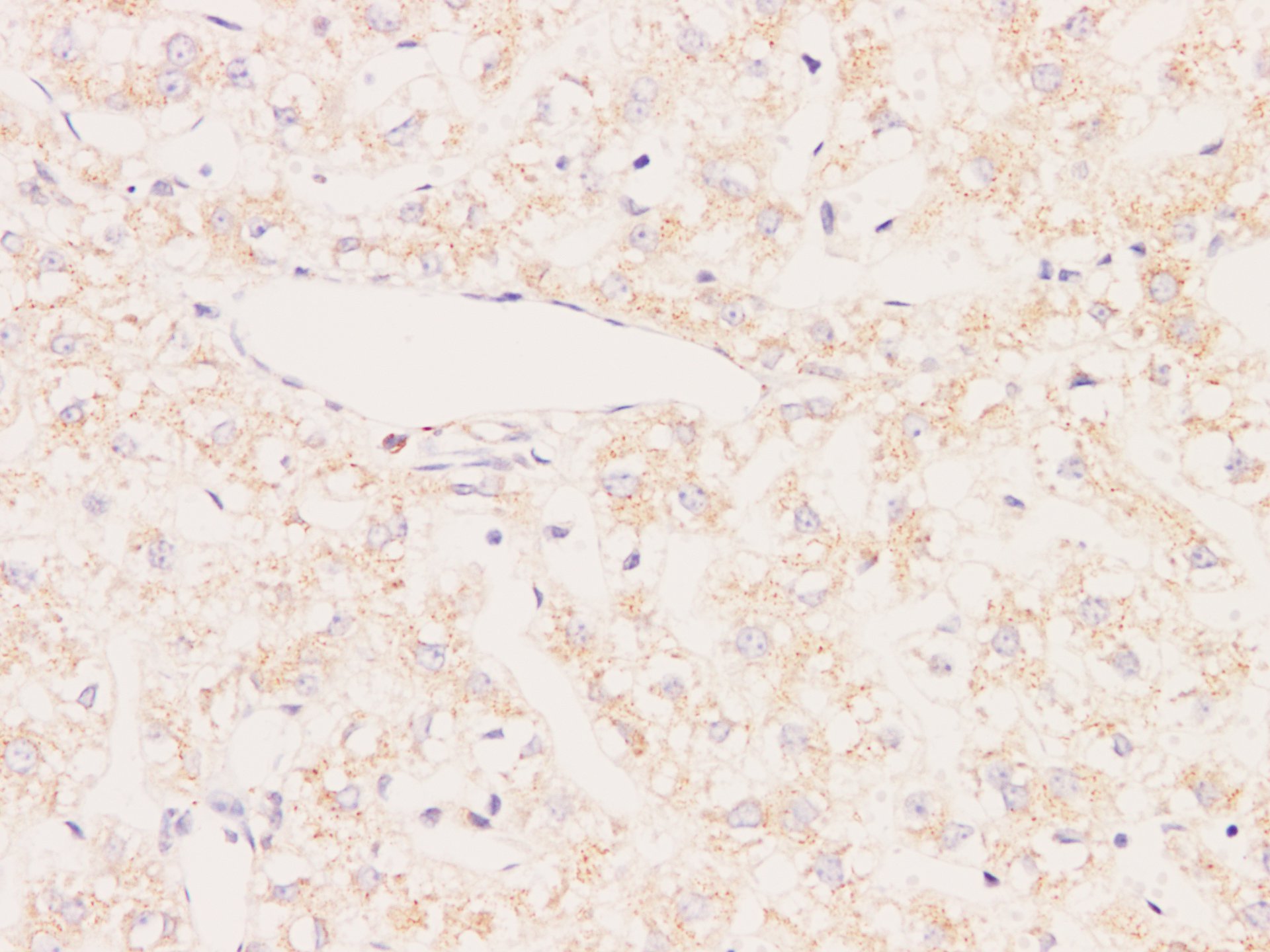

Supplement: Figure S3 — 32 photos comprising Figure 3. [file peerj-08-8254-s003.zip › Fig 3l.jpg]

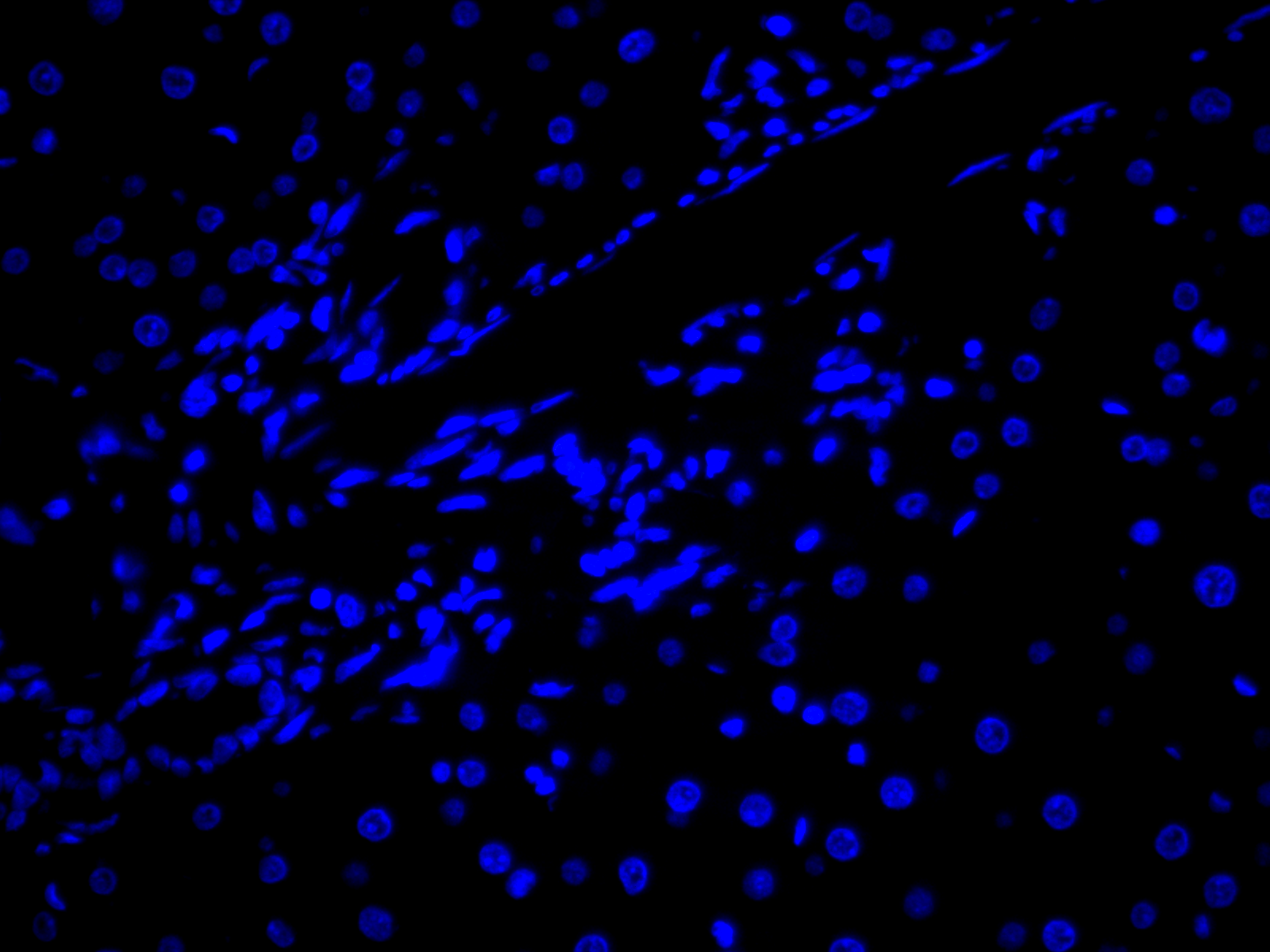

Supplement: Figure S3 — 32 photos comprising Figure 3. [file peerj-08-8254-s003.zip › Fig 3j.png]

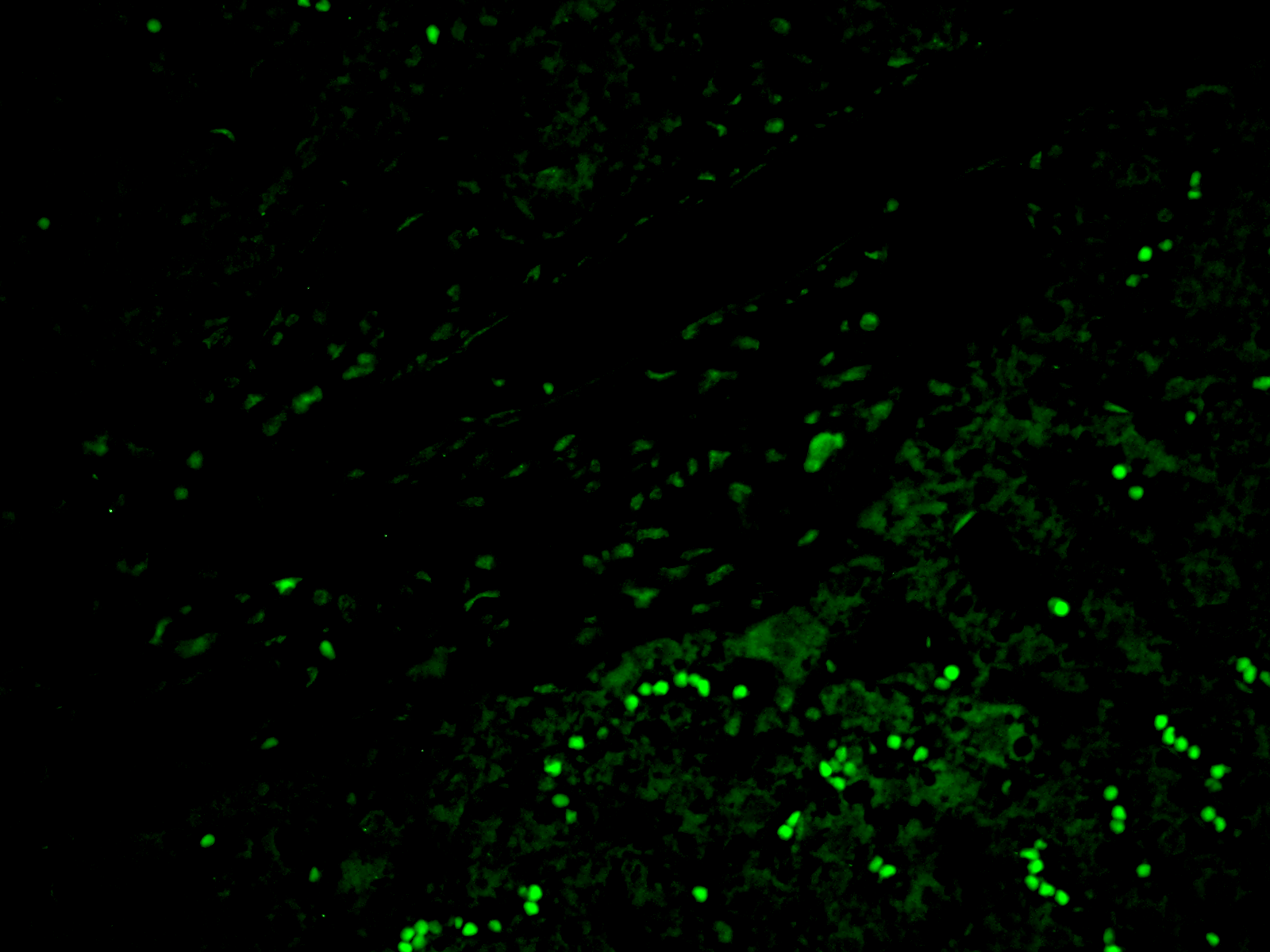

Supplement: Figure S3 — 32 photos comprising Figure 3. [file peerj-08-8254-s003.zip › Fig 3i.png]

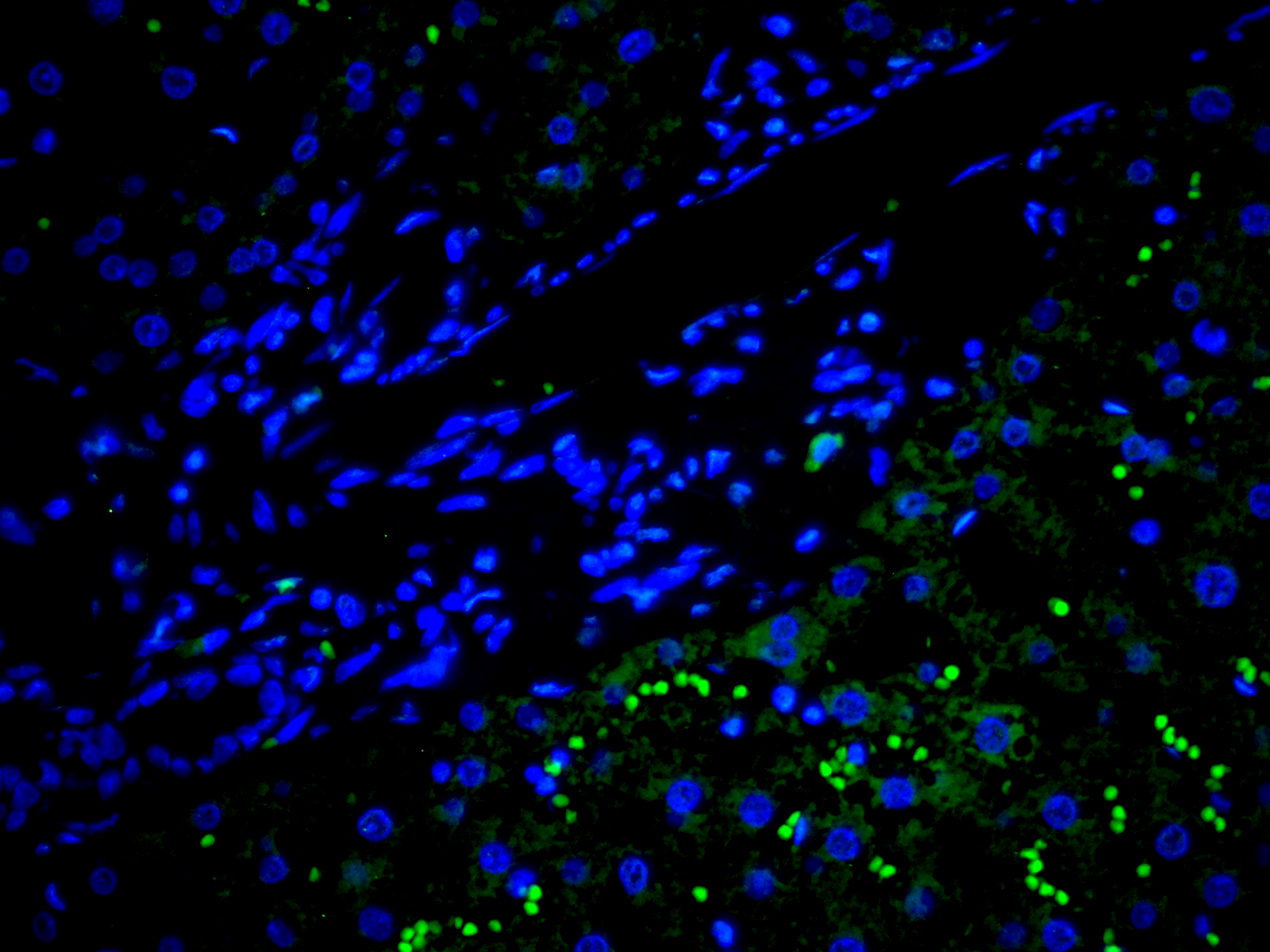

Supplement: Figure S3 — 32 photos comprising Figure 3. [file peerj-08-8254-s003.zip › Fig 3k.png]

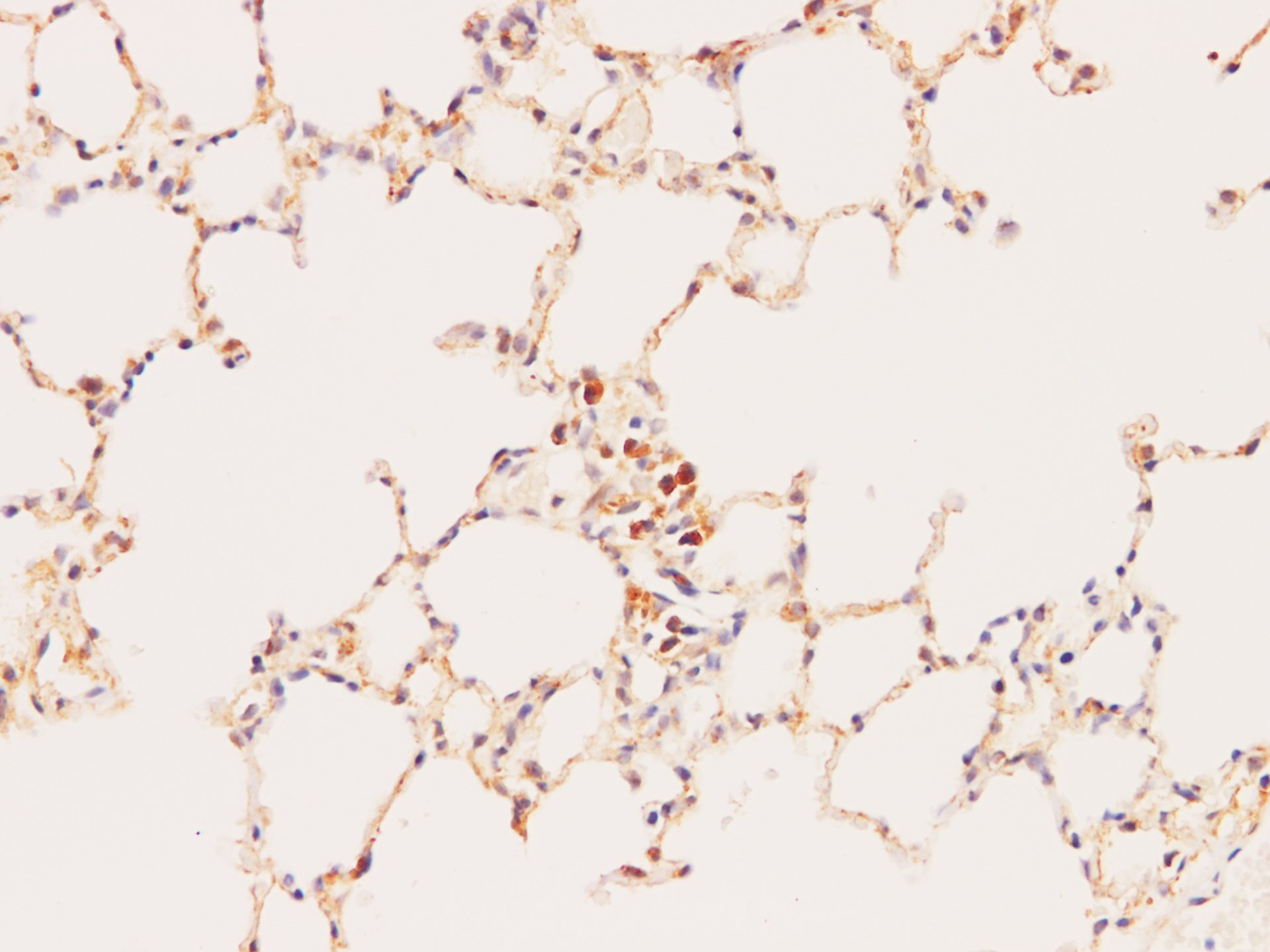

Supplement: Figure S3 — 32 photos comprising Figure 3. [file peerj-08-8254-s003.zip › Fig 3p.jpg]

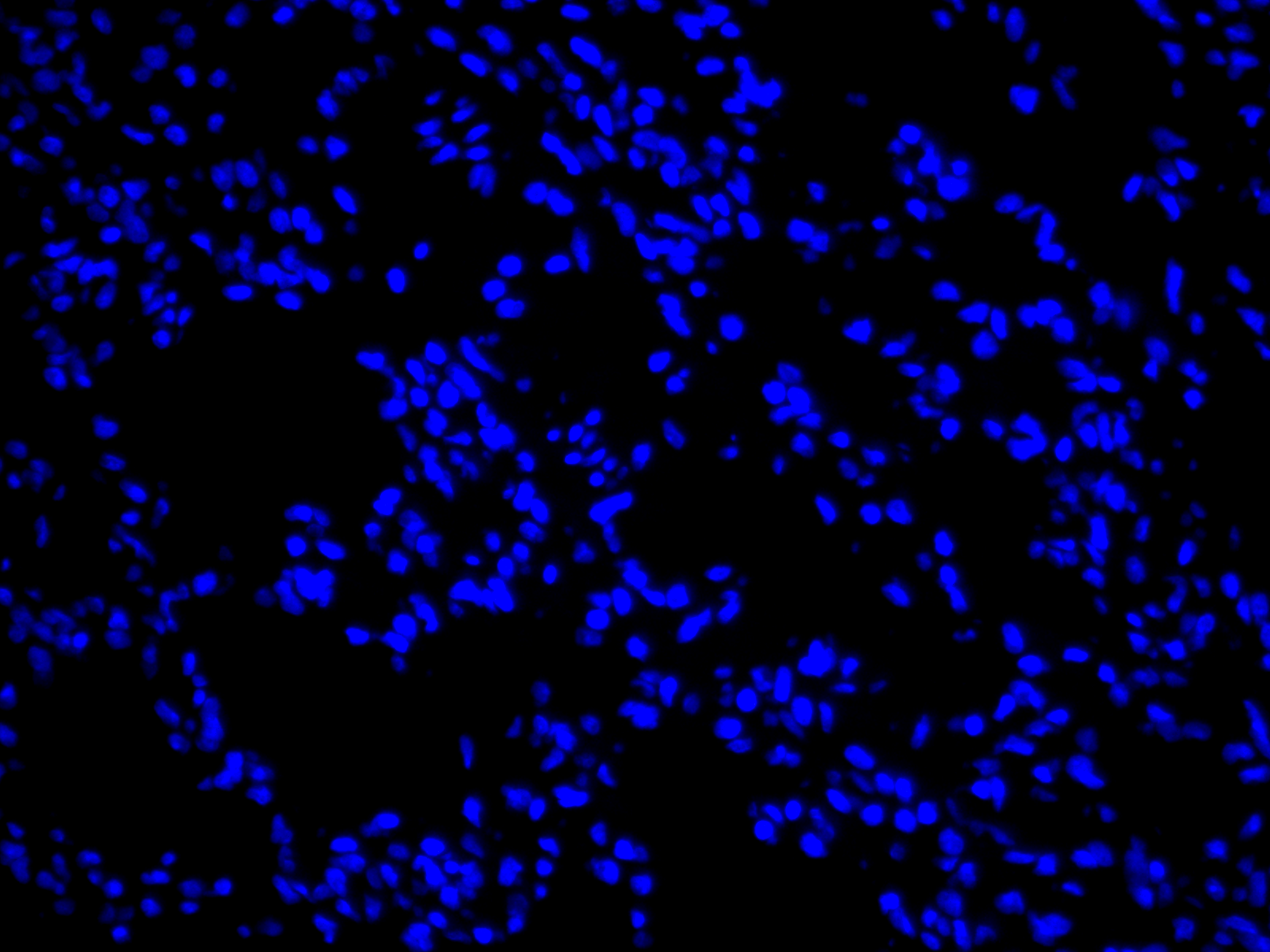

Supplement: Figure S3 — 32 photos comprising Figure 3. [file peerj-08-8254-s003.zip › Fig 3n.png]

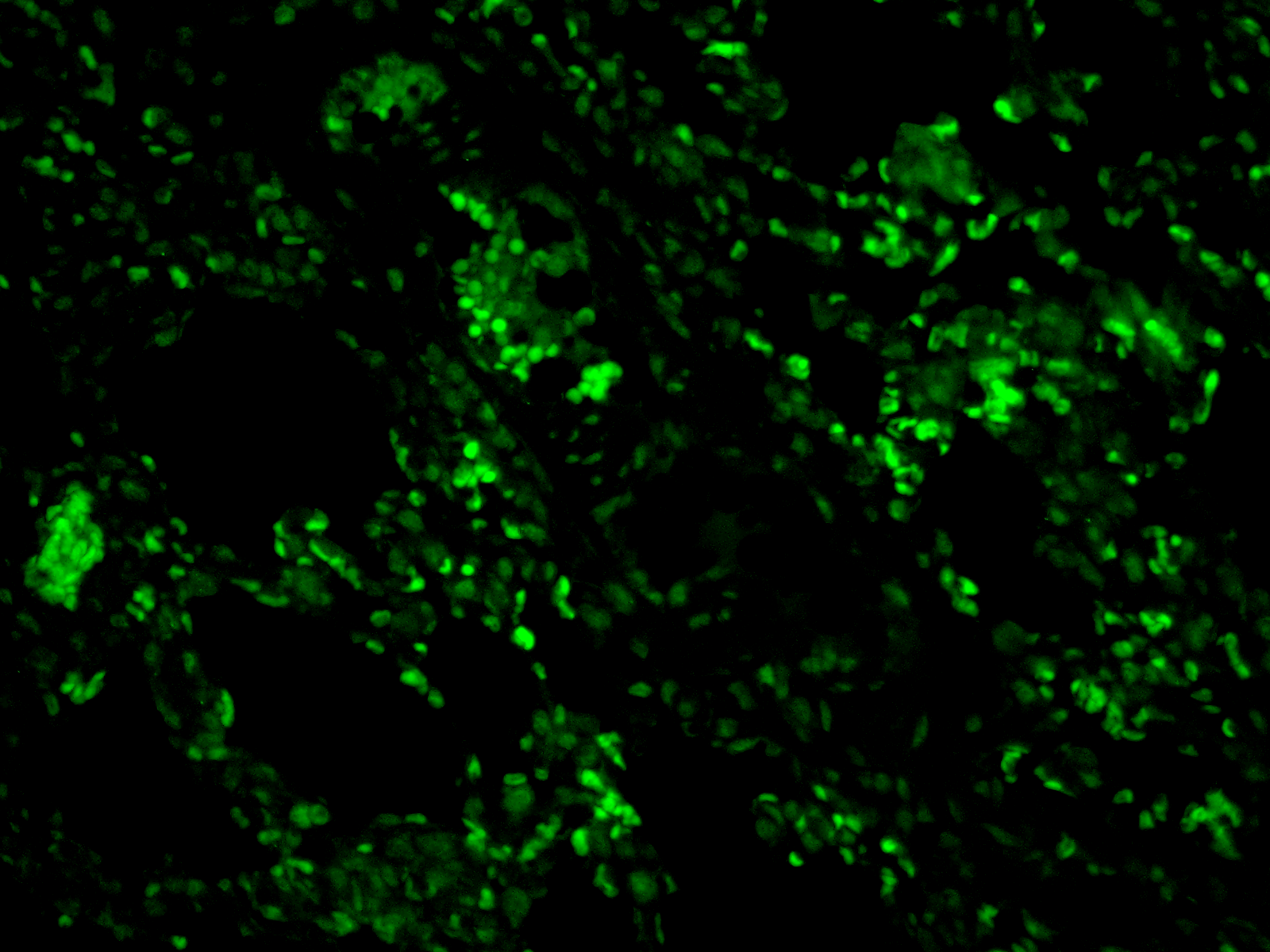

Supplement: Figure S3 — 32 photos comprising Figure 3. [file peerj-08-8254-s003.zip › Fig 3m.png]

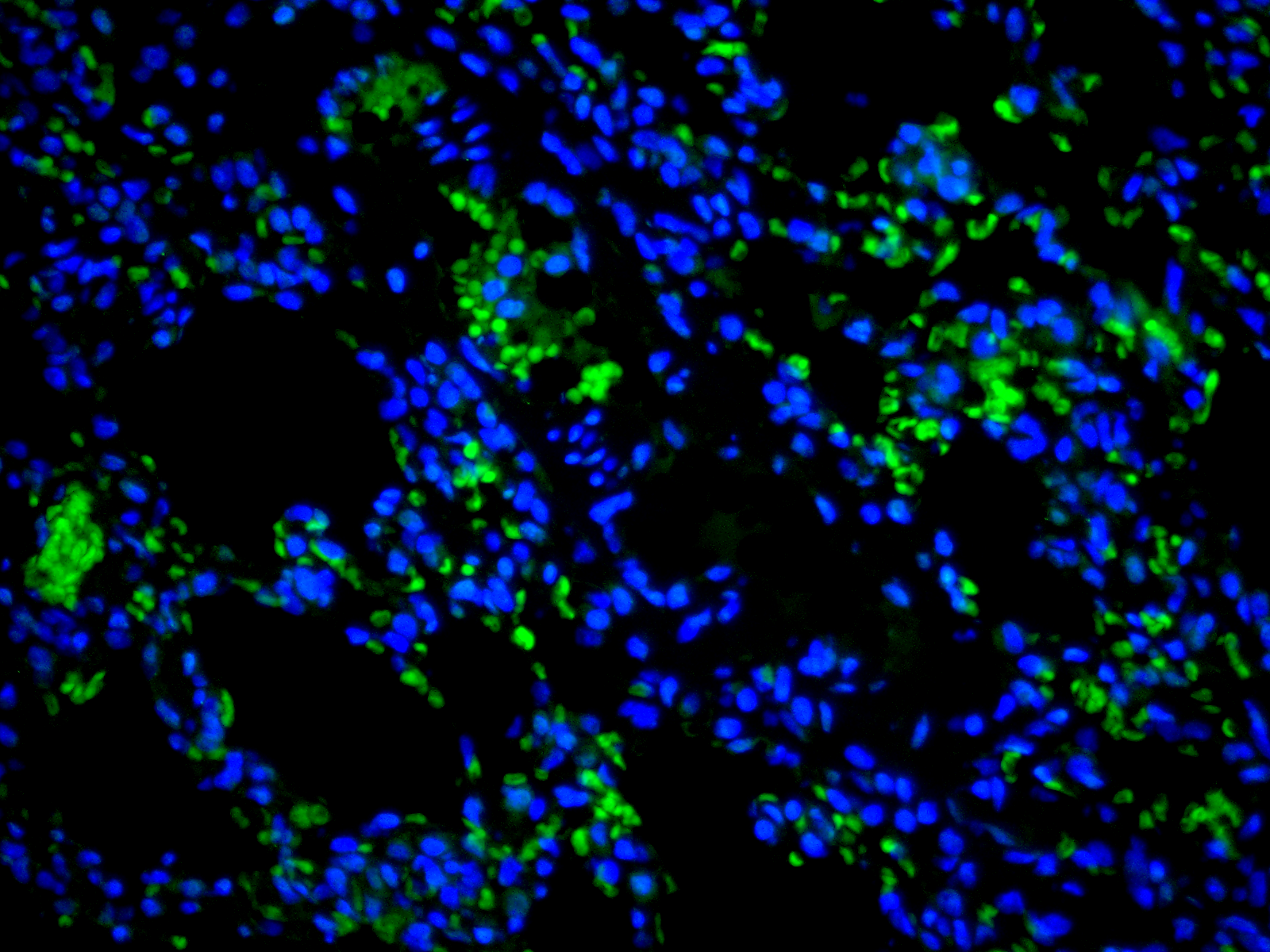

Supplement: Figure S3 — 32 photos comprising Figure 3. [file peerj-08-8254-s003.zip › Fig 3o.png]

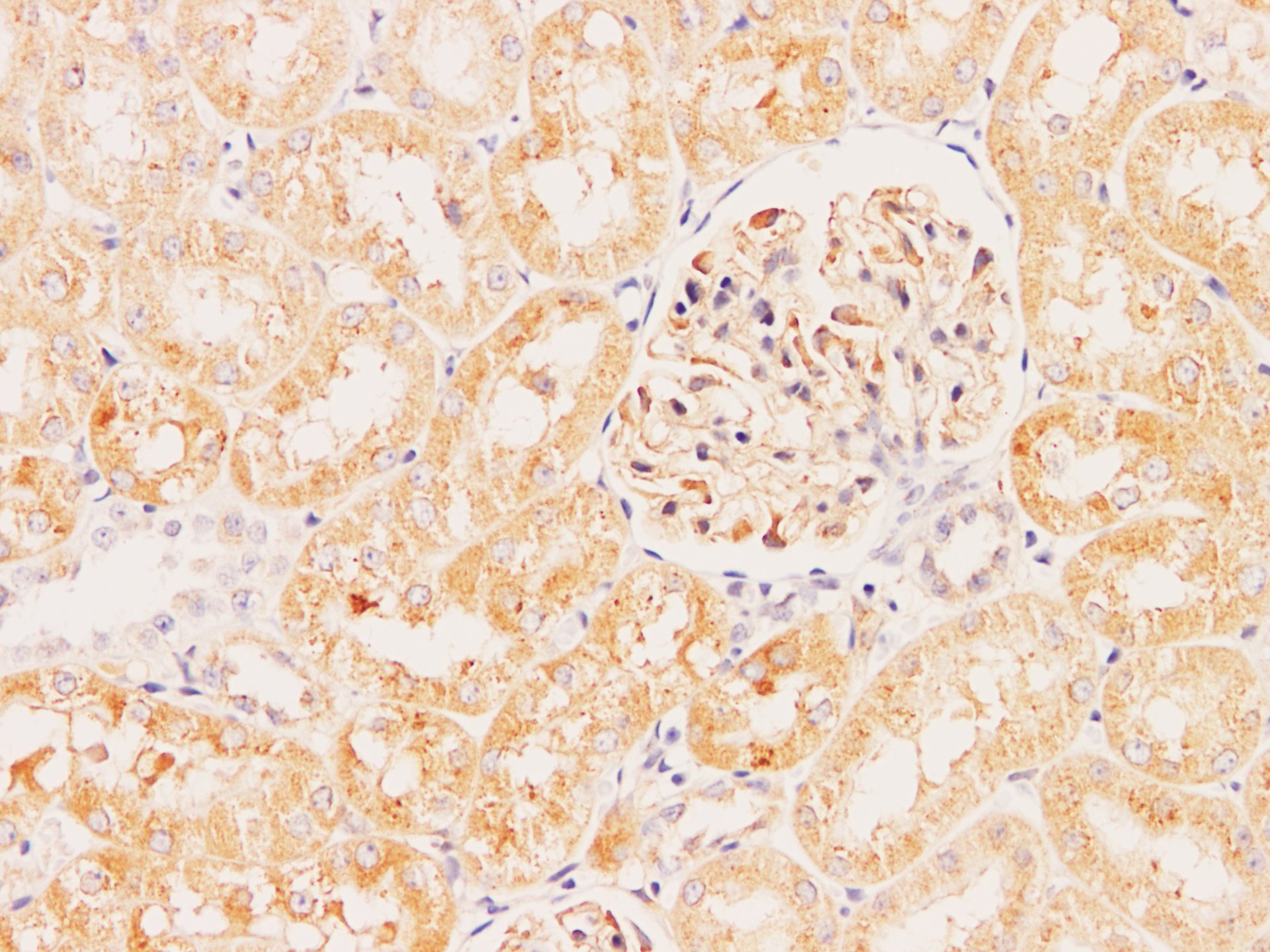

Supplement: Figure S3 — 32 photos comprising Figure 3. [file peerj-08-8254-s003.zip › Fig 3t.jpg]

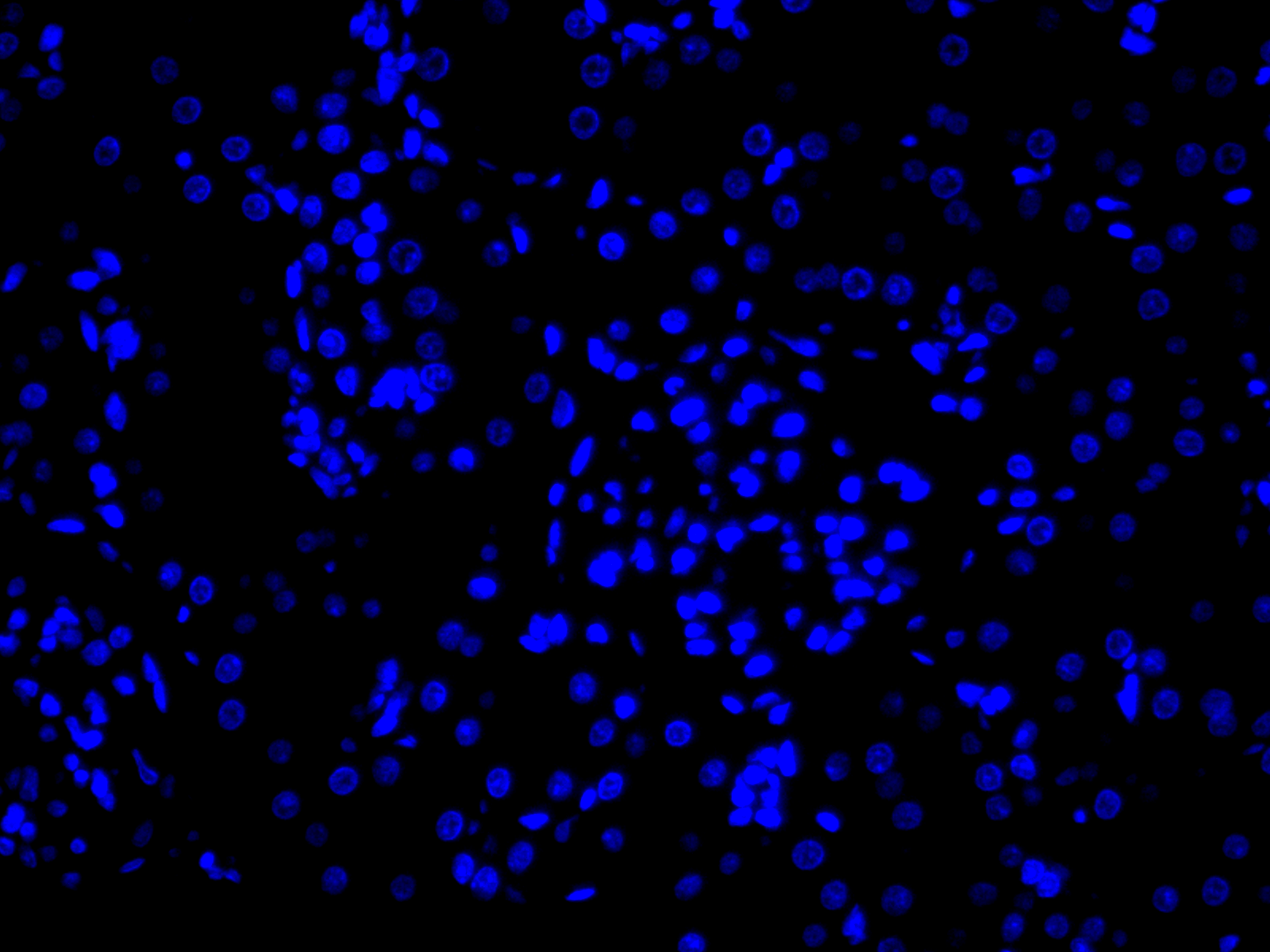

Supplement: Figure S3 — 32 photos comprising Figure 3. [file peerj-08-8254-s003.zip › Fig 3r.png]

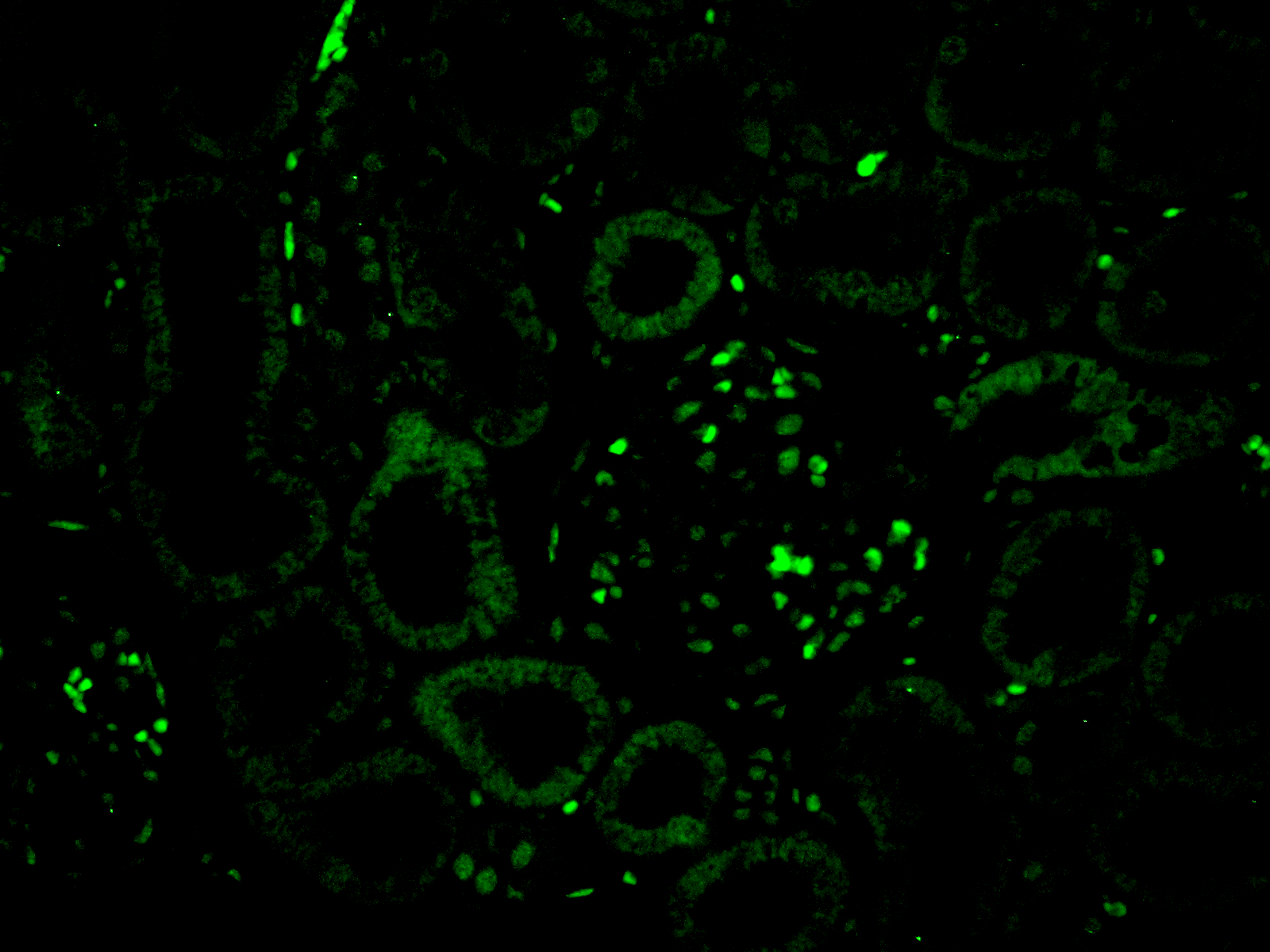

Supplement: Figure S3 — 32 photos comprising Figure 3. [file peerj-08-8254-s003.zip › Fig 3q.png]

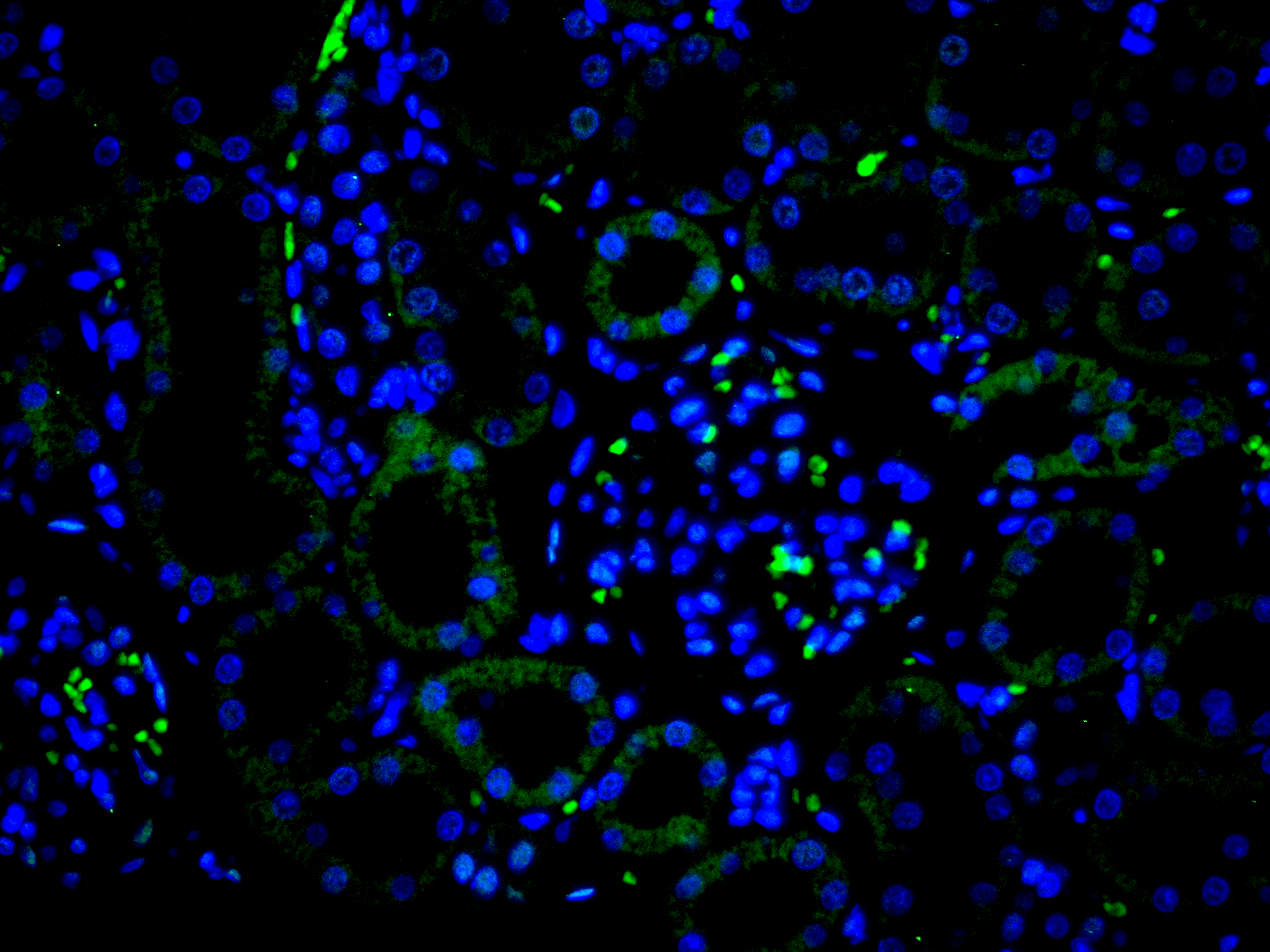

Supplement: Figure S3 — 32 photos comprising Figure 3. [file peerj-08-8254-s003.zip › Fig 3s.png]

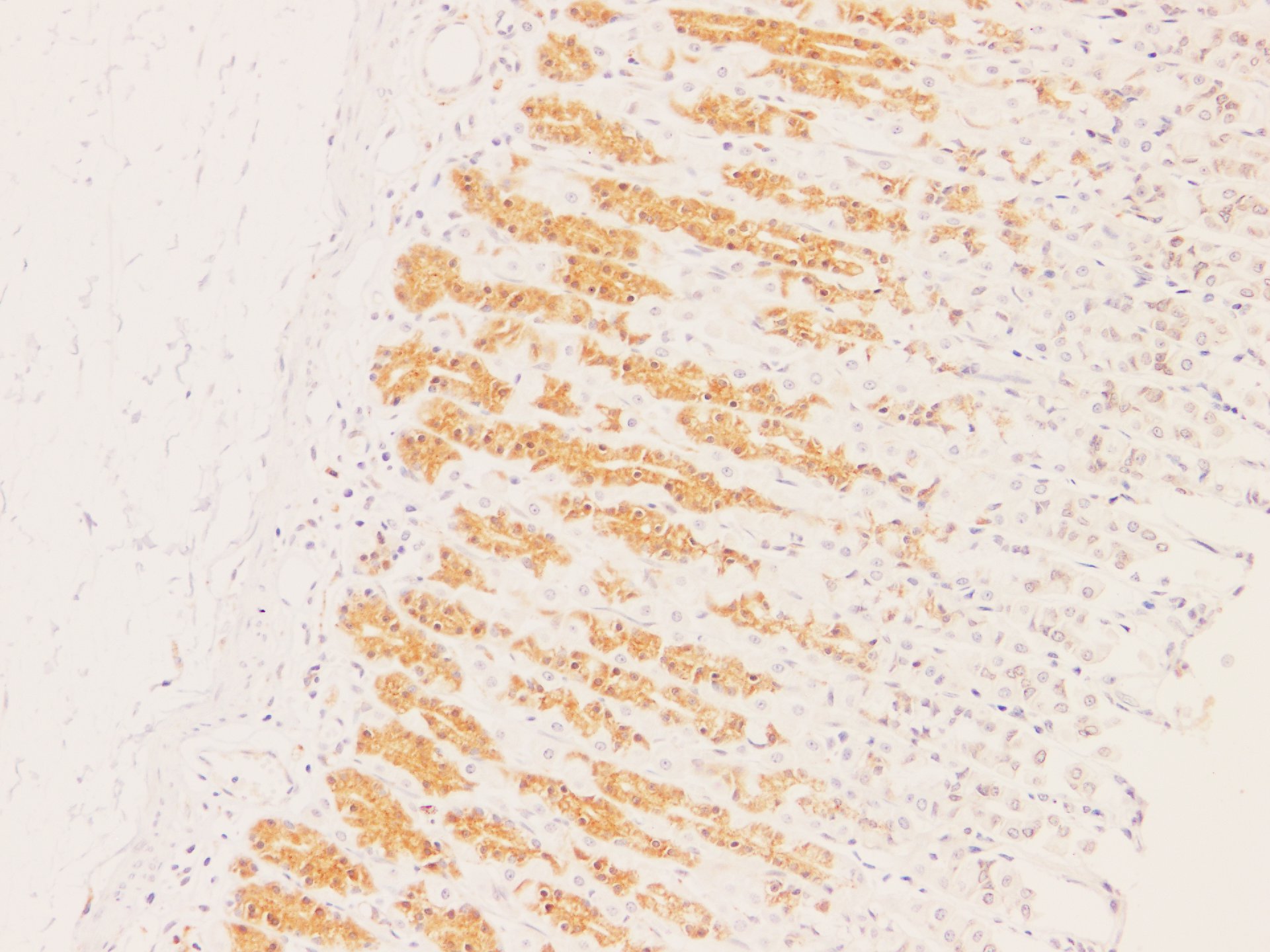

Supplement: Figure S3 — 32 photos comprising Figure 3. [file peerj-08-8254-s003.zip › Fig 3x.jpg]

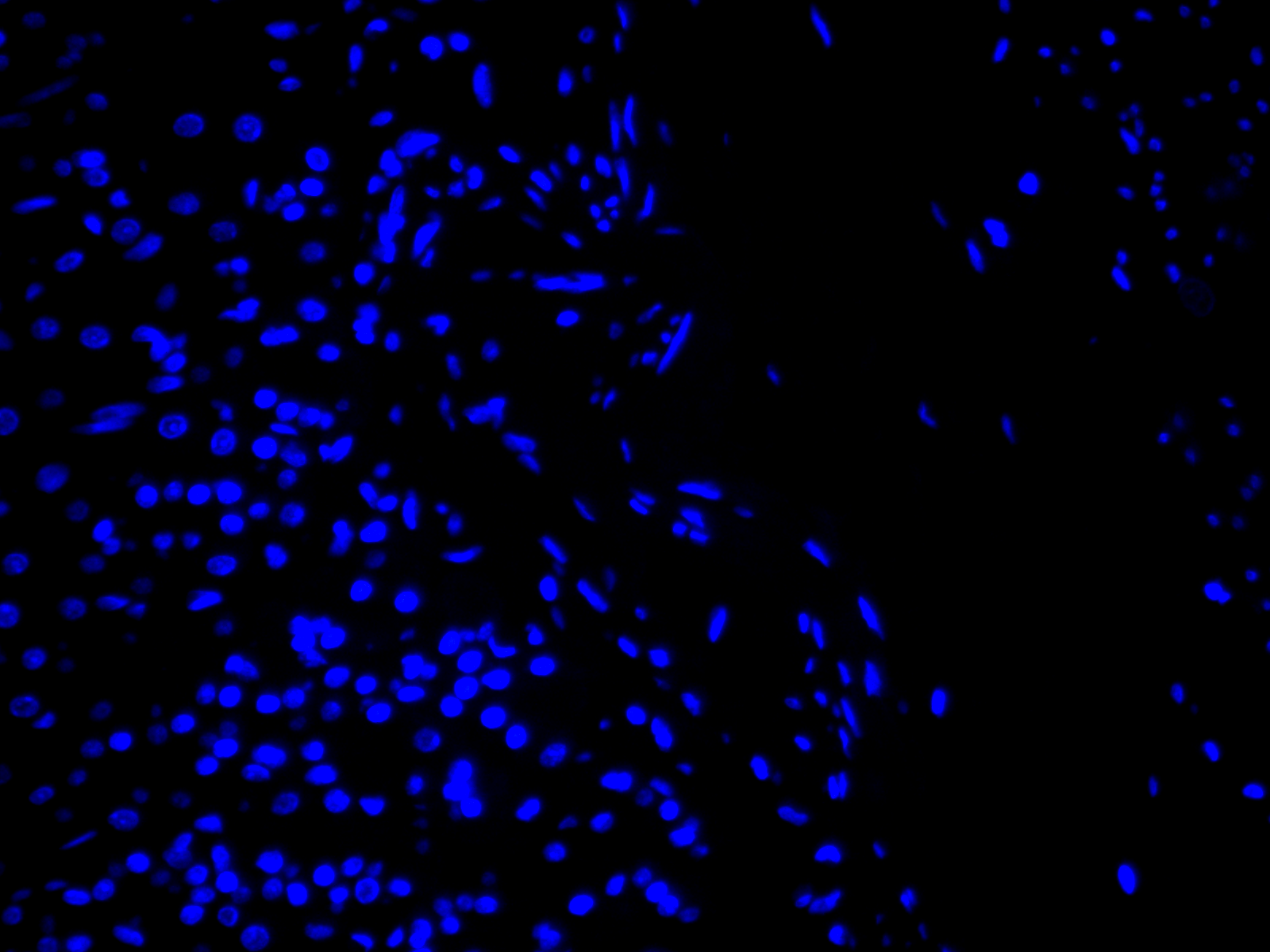

Supplement: Figure S3 — 32 photos comprising Figure 3. [file peerj-08-8254-s003.zip › Fig 3v.png]

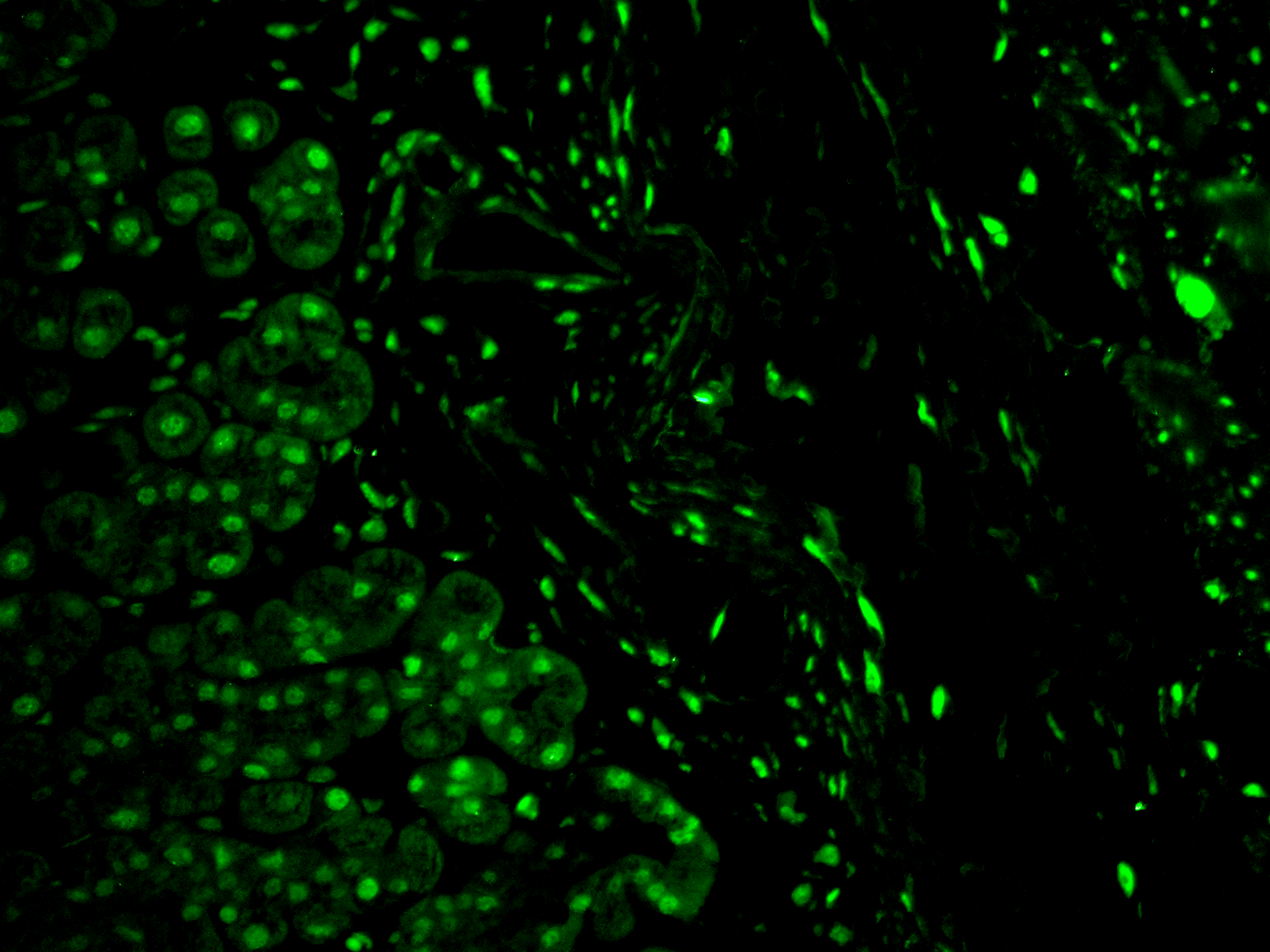

Supplement: Figure S3 — 32 photos comprising Figure 3. [file peerj-08-8254-s003.zip › Fig 3u.png]

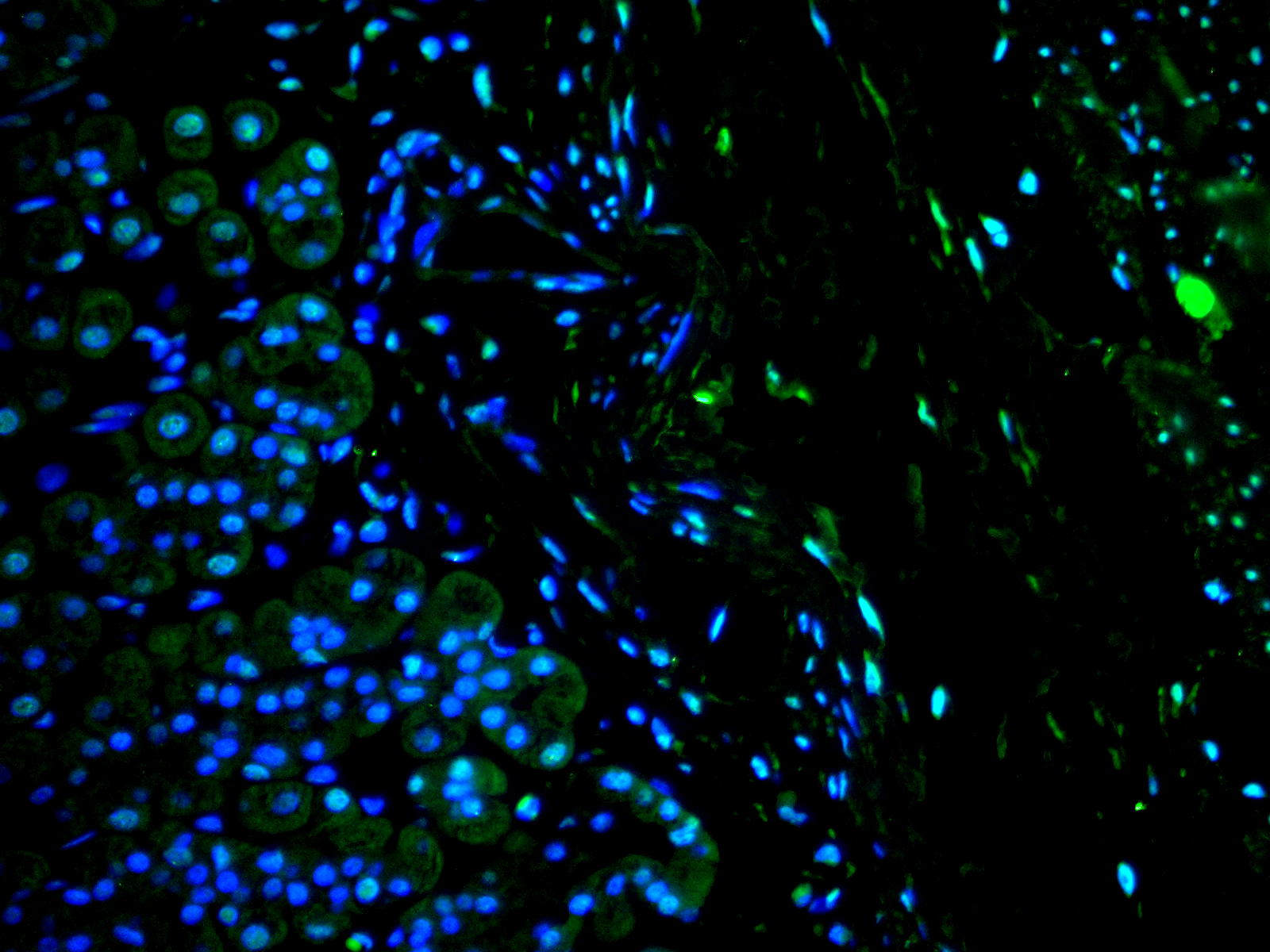

Supplement: Figure S3 — 32 photos comprising Figure 3. [file peerj-08-8254-s003.zip › Fig 3w.png]

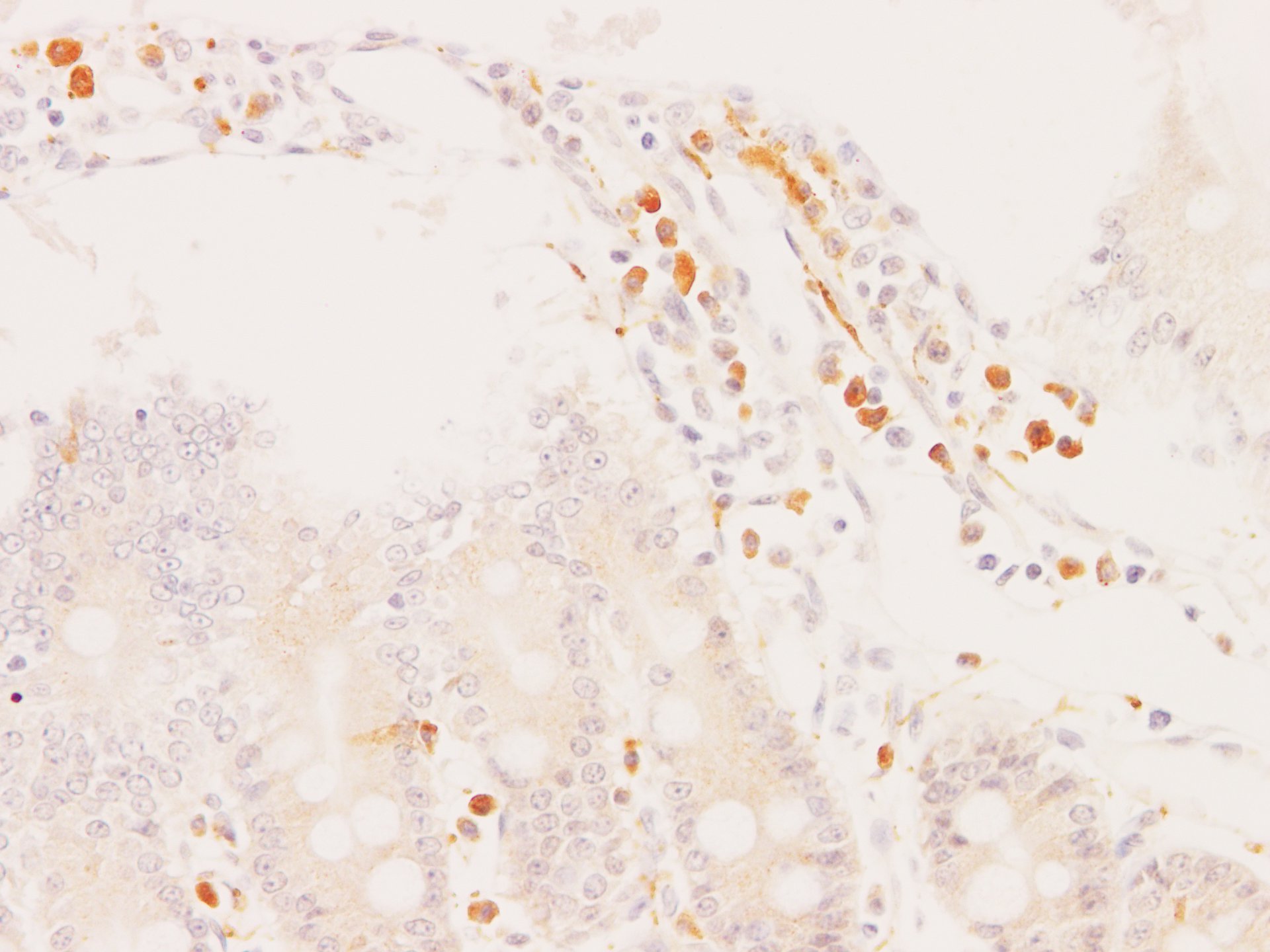

Supplement: Figure S3 — 32 photos comprising Figure 3. [file peerj-08-8254-s003.zip › Fig 3bb.jpg]

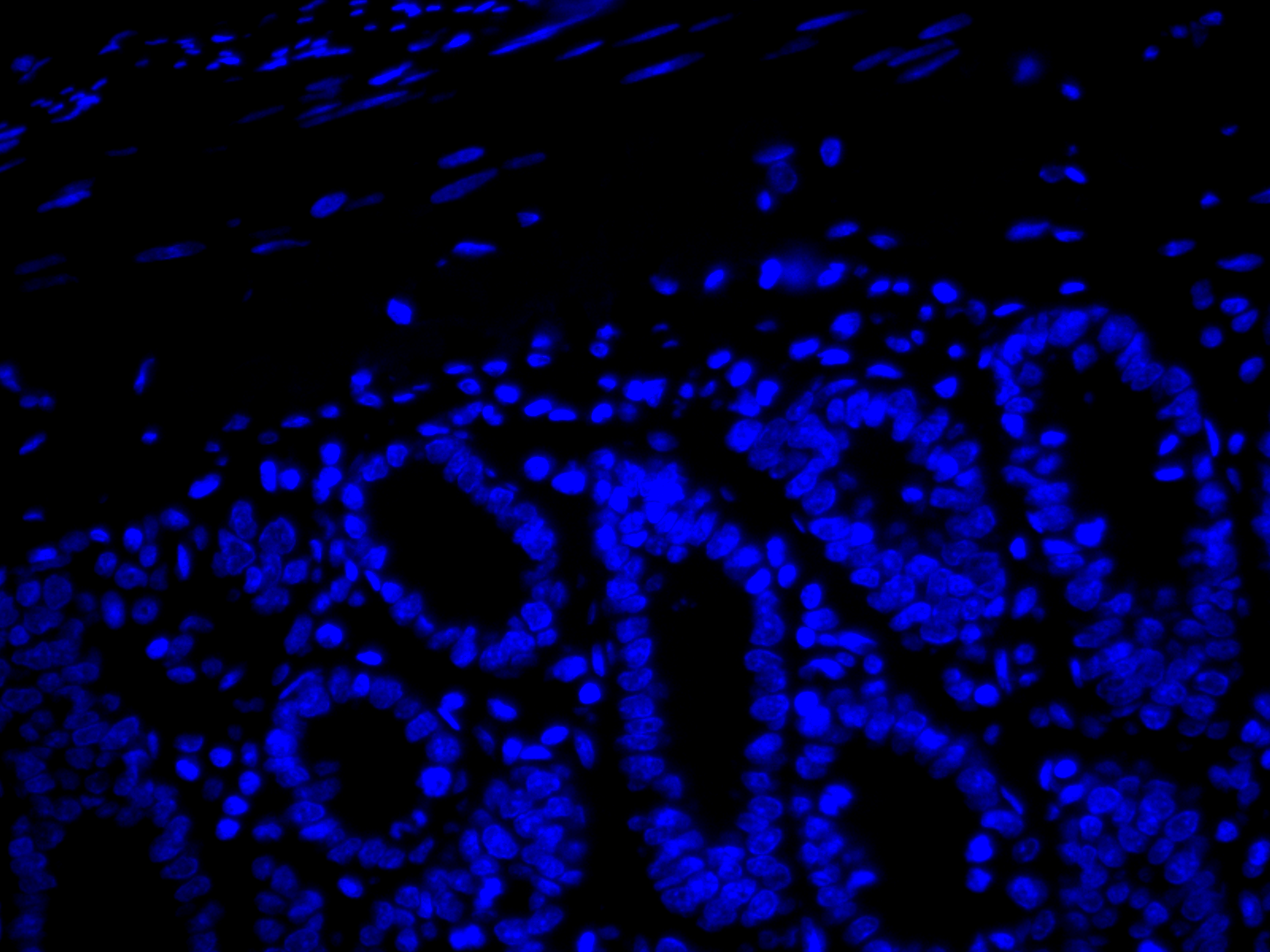

Supplement: Figure S3 — 32 photos comprising Figure 3. [file peerj-08-8254-s003.zip › Fig 3z.png]

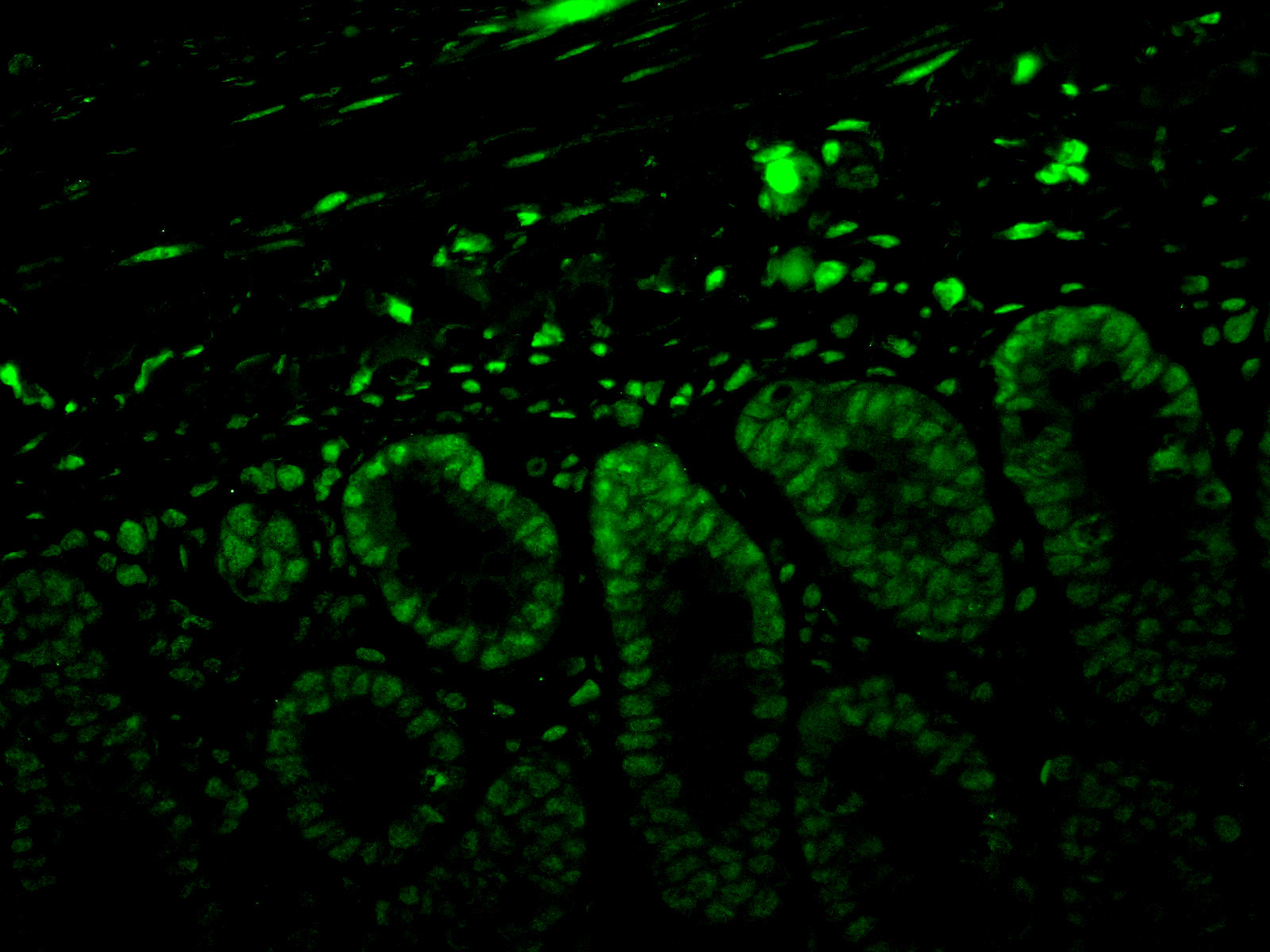

Supplement: Figure S3 — 32 photos comprising Figure 3. [file peerj-08-8254-s003.zip › Fig 3y.png]

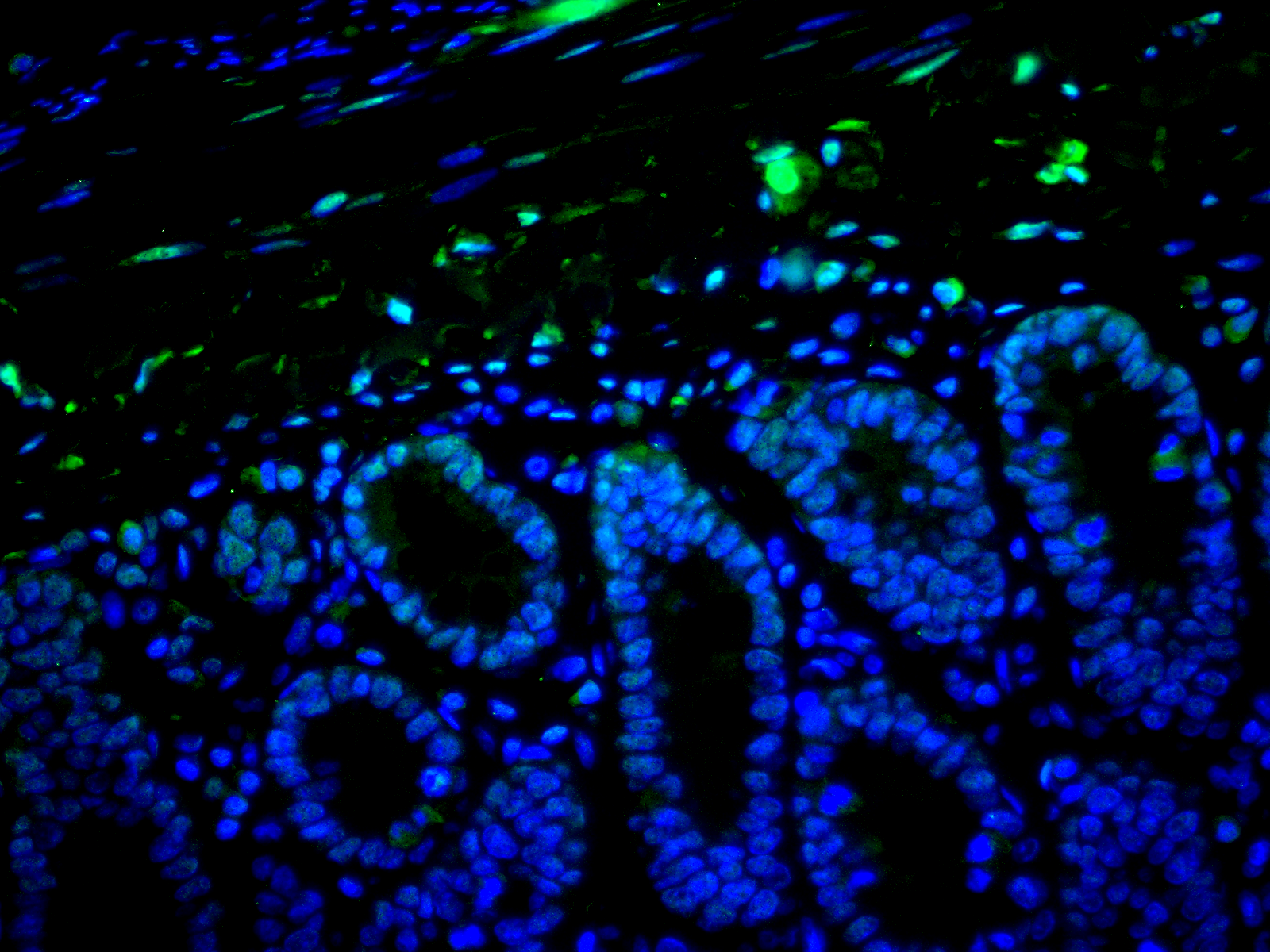

Supplement: Figure S3 — 32 photos comprising Figure 3. [file peerj-08-8254-s003.zip › Fig 3aa.png]

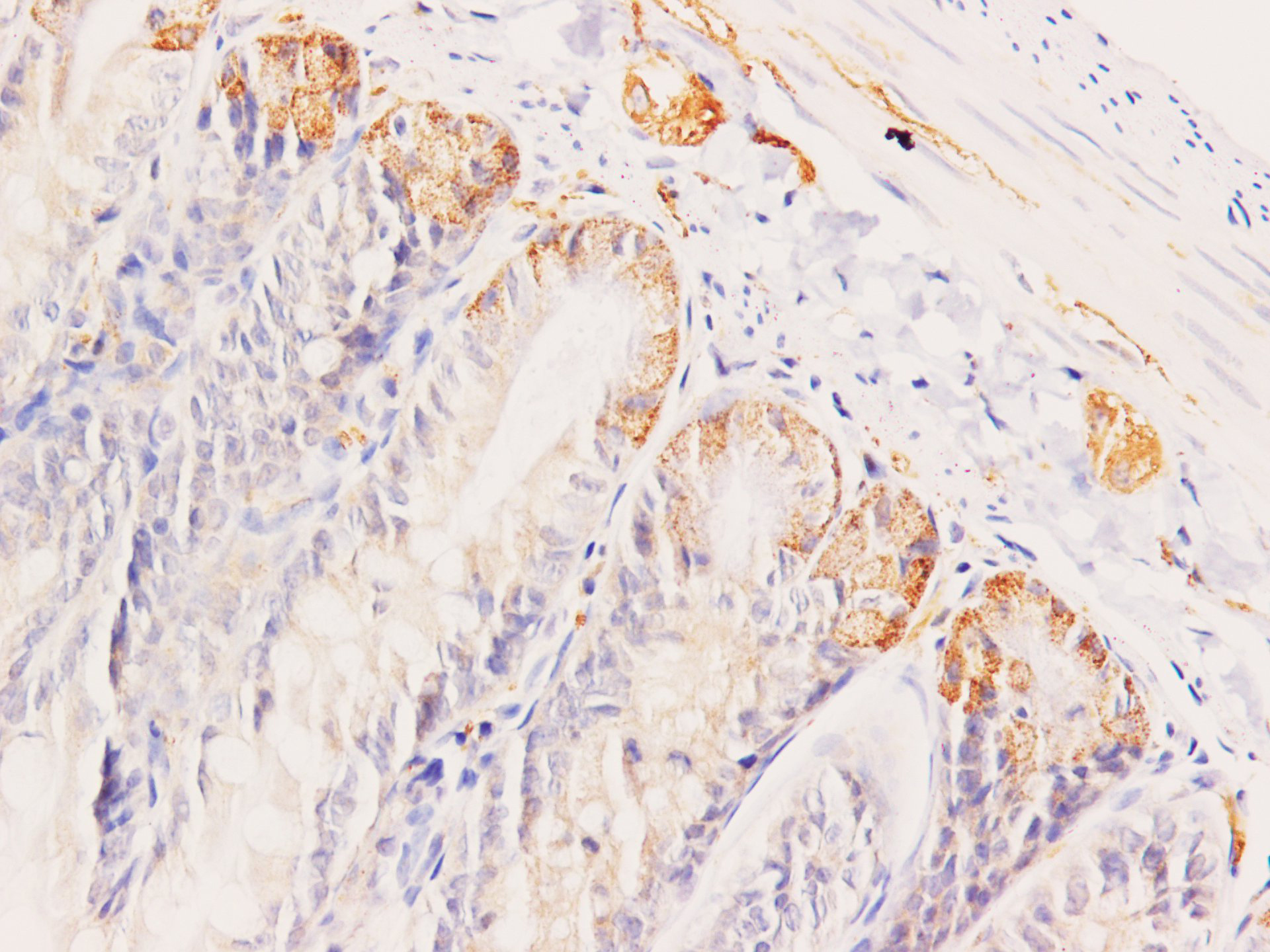

Supplement: Figure S3 — 32 photos comprising Figure 3. [file peerj-08-8254-s003.zip › Fig 3ff.jpg]

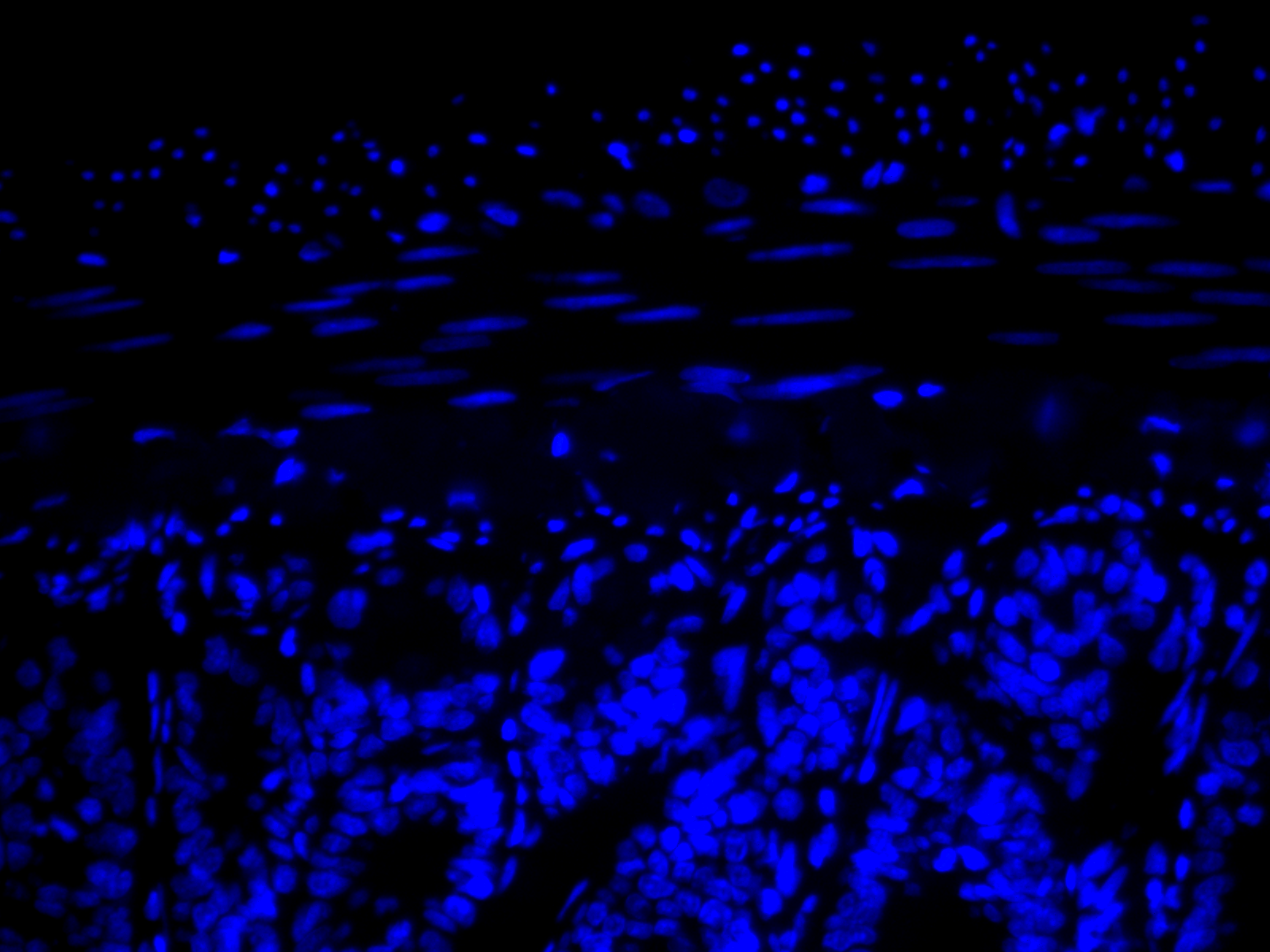

Supplement: Figure S3 — 32 photos comprising Figure 3. [file peerj-08-8254-s003.zip › Fig 3dd.png]

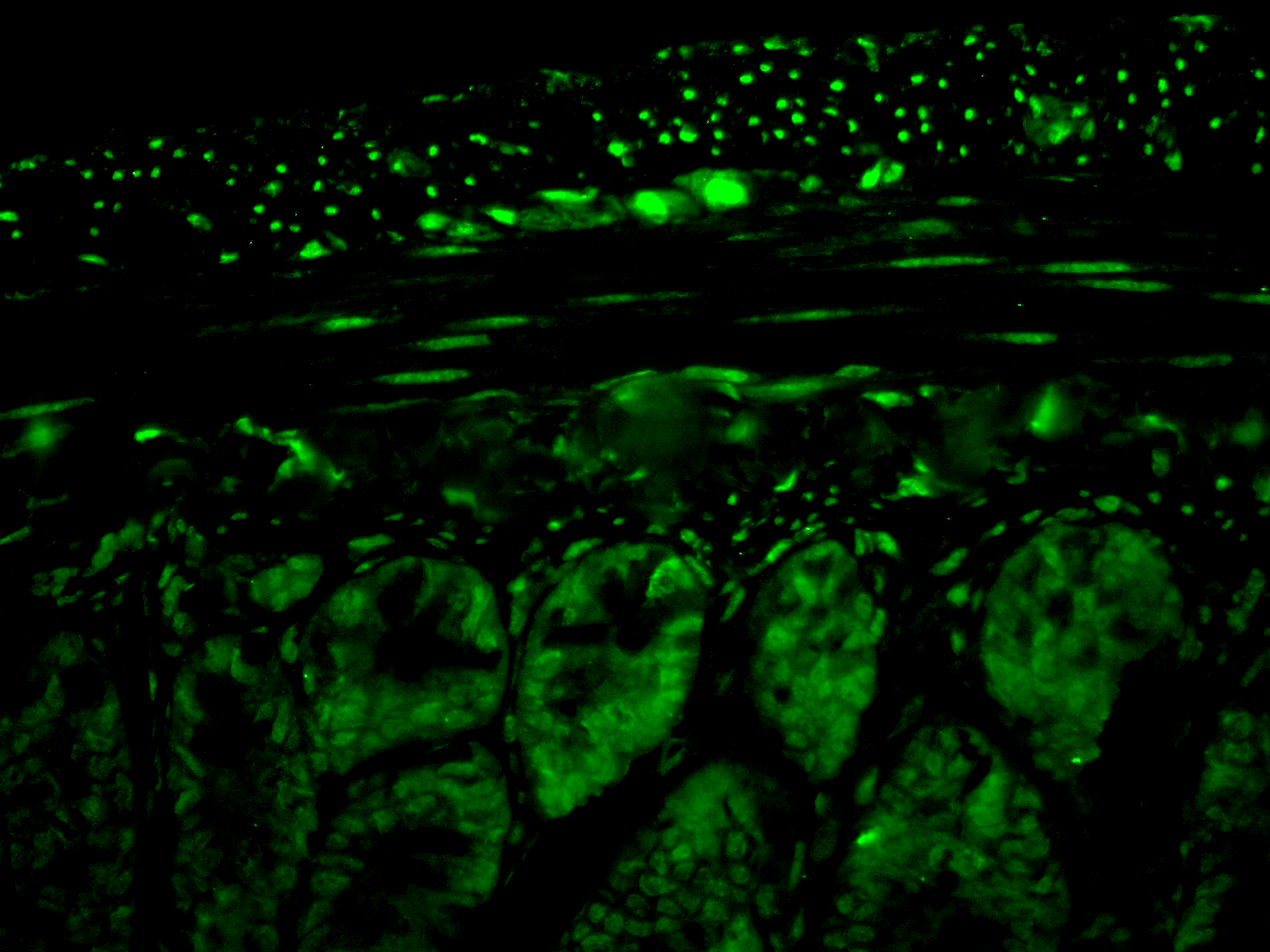

Supplement: Figure S3 — 32 photos comprising Figure 3. [file peerj-08-8254-s003.zip › Fig 3cc.png]

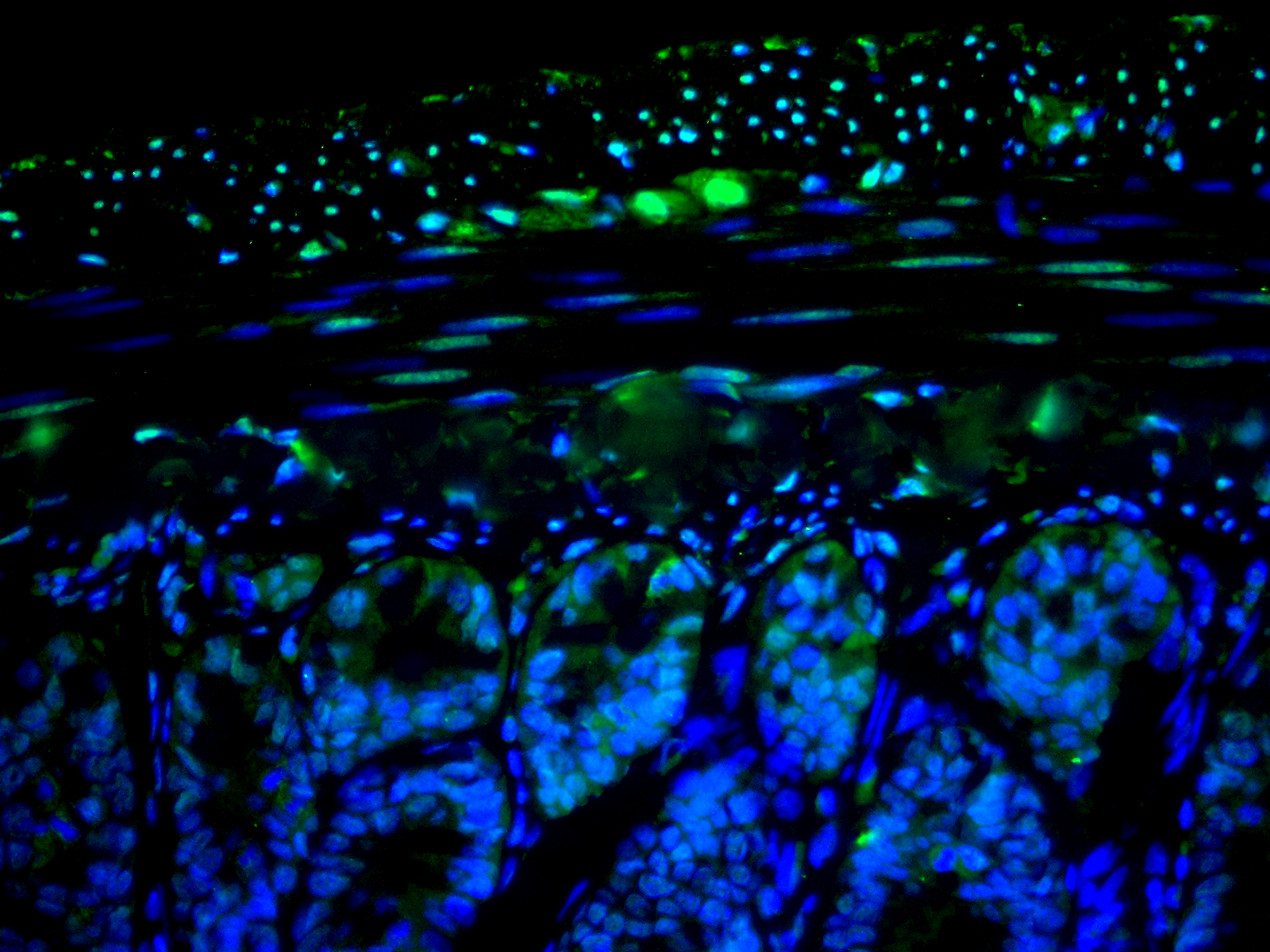

Supplement: Figure S3 — 32 photos comprising Figure 3. [file peerj-08-8254-s003.zip › Fig 3ee.png]

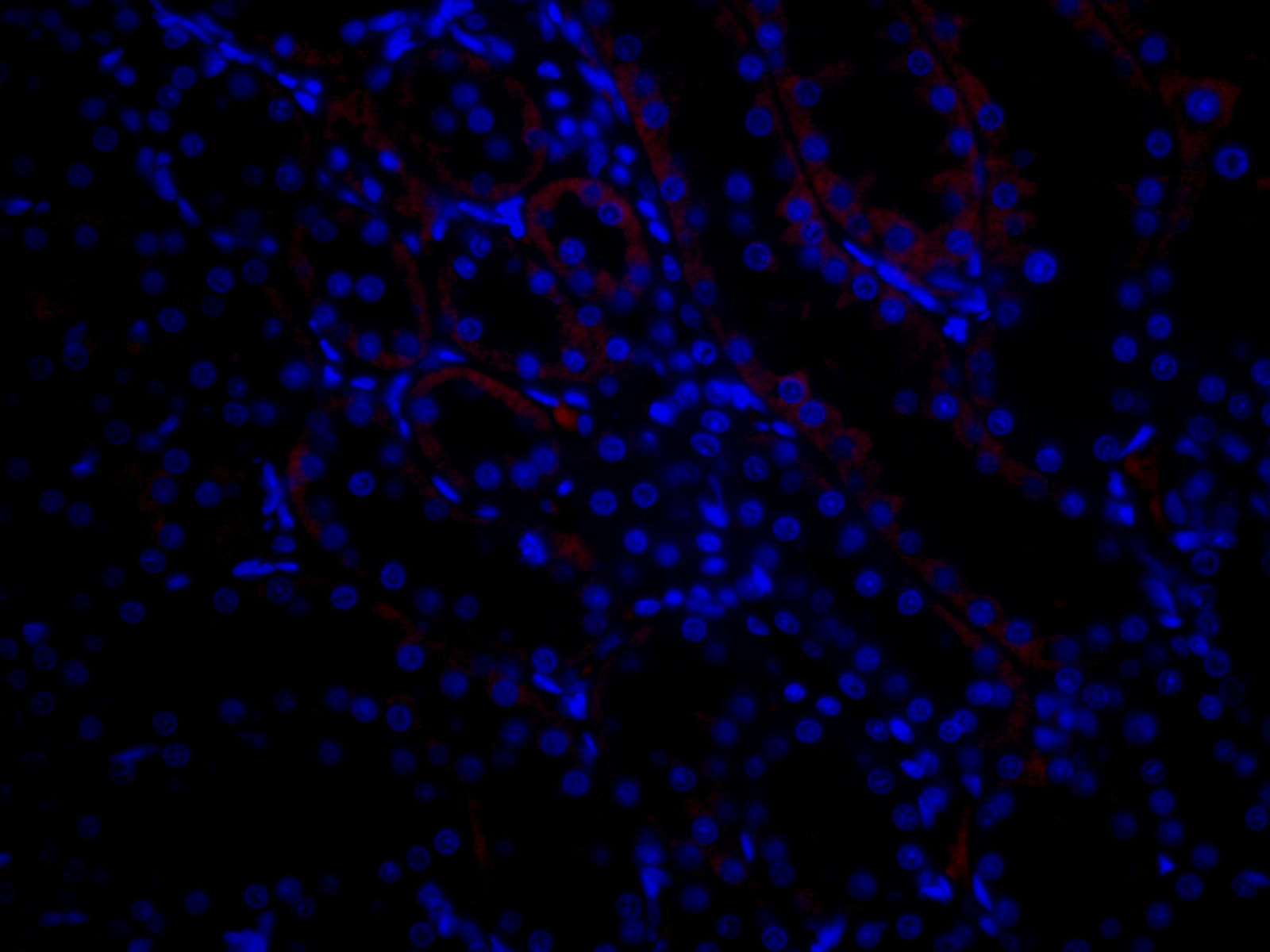

Supplement: Figure S4 — 15 photos comprising Figure 4. [file peerj-08-8254-s004.zip › FIg 4O.tif]

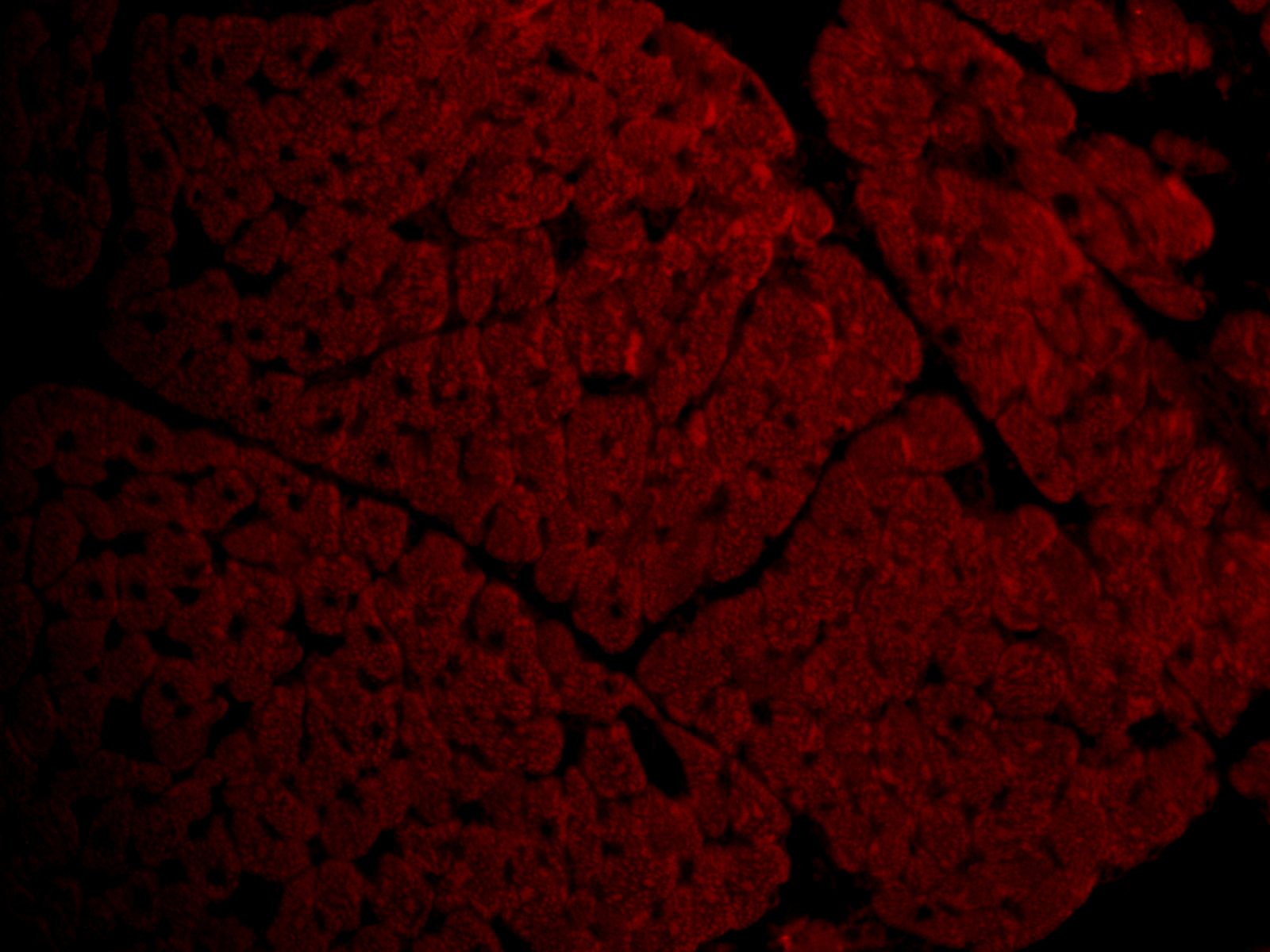

Supplement: Figure S4 — 15 photos comprising Figure 4. [file peerj-08-8254-s004.zip › Fig 4A.tif]

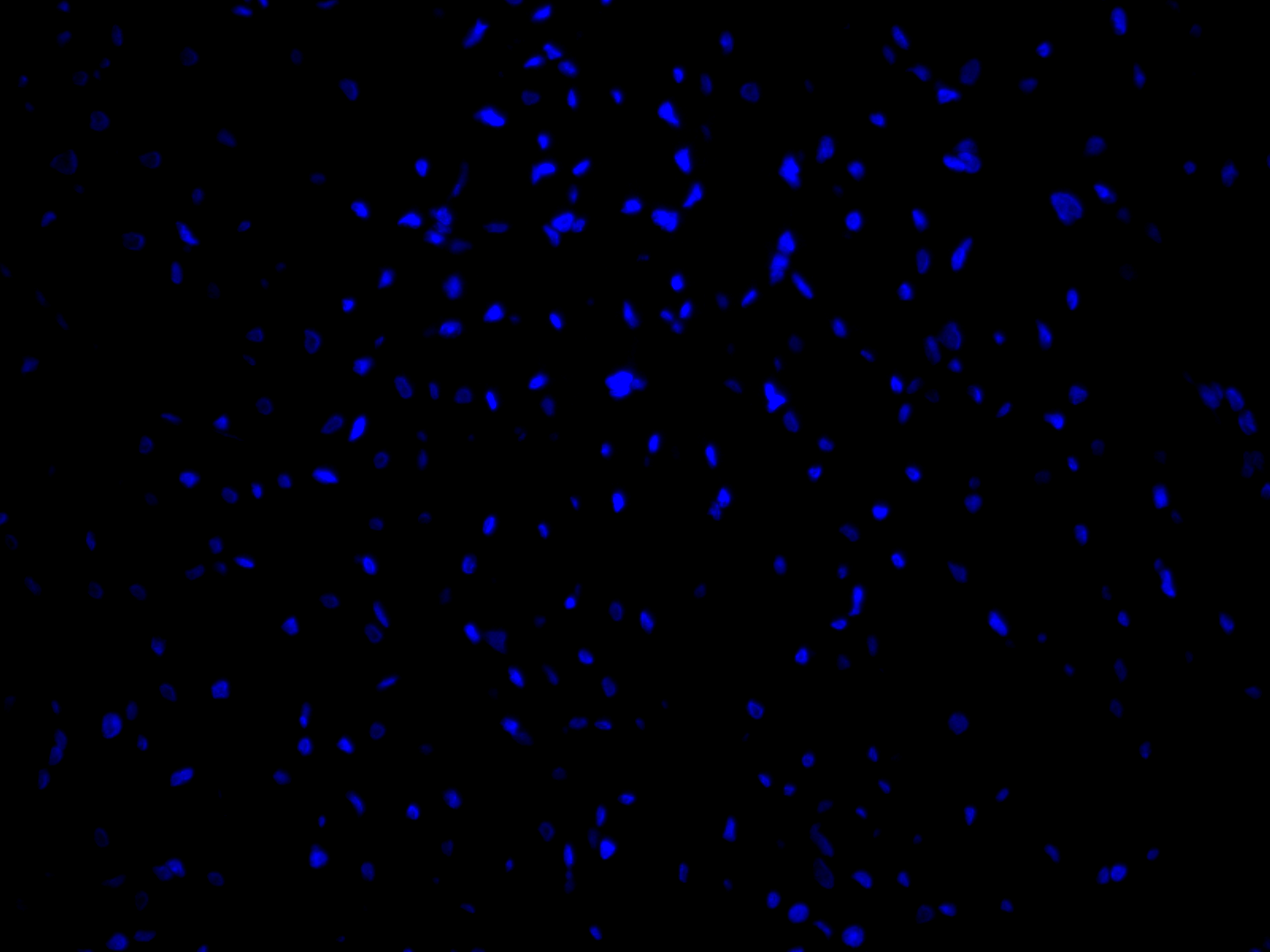

Supplement: Figure S4 — 15 photos comprising Figure 4. [file peerj-08-8254-s004.zip › Fig 4B.tif]

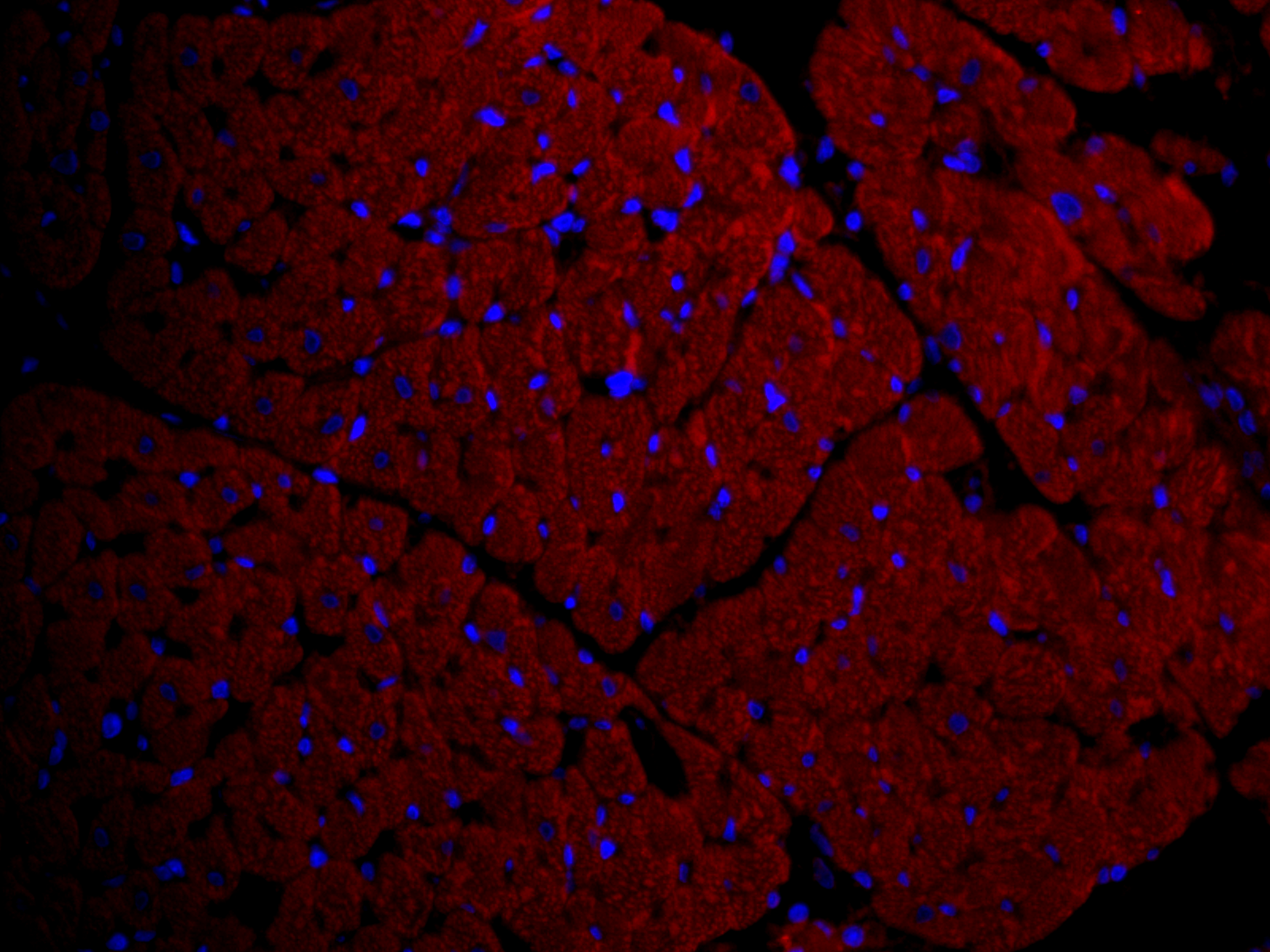

Supplement: Figure S4 — 15 photos comprising Figure 4. [file peerj-08-8254-s004.zip › Fig 4C.tif]

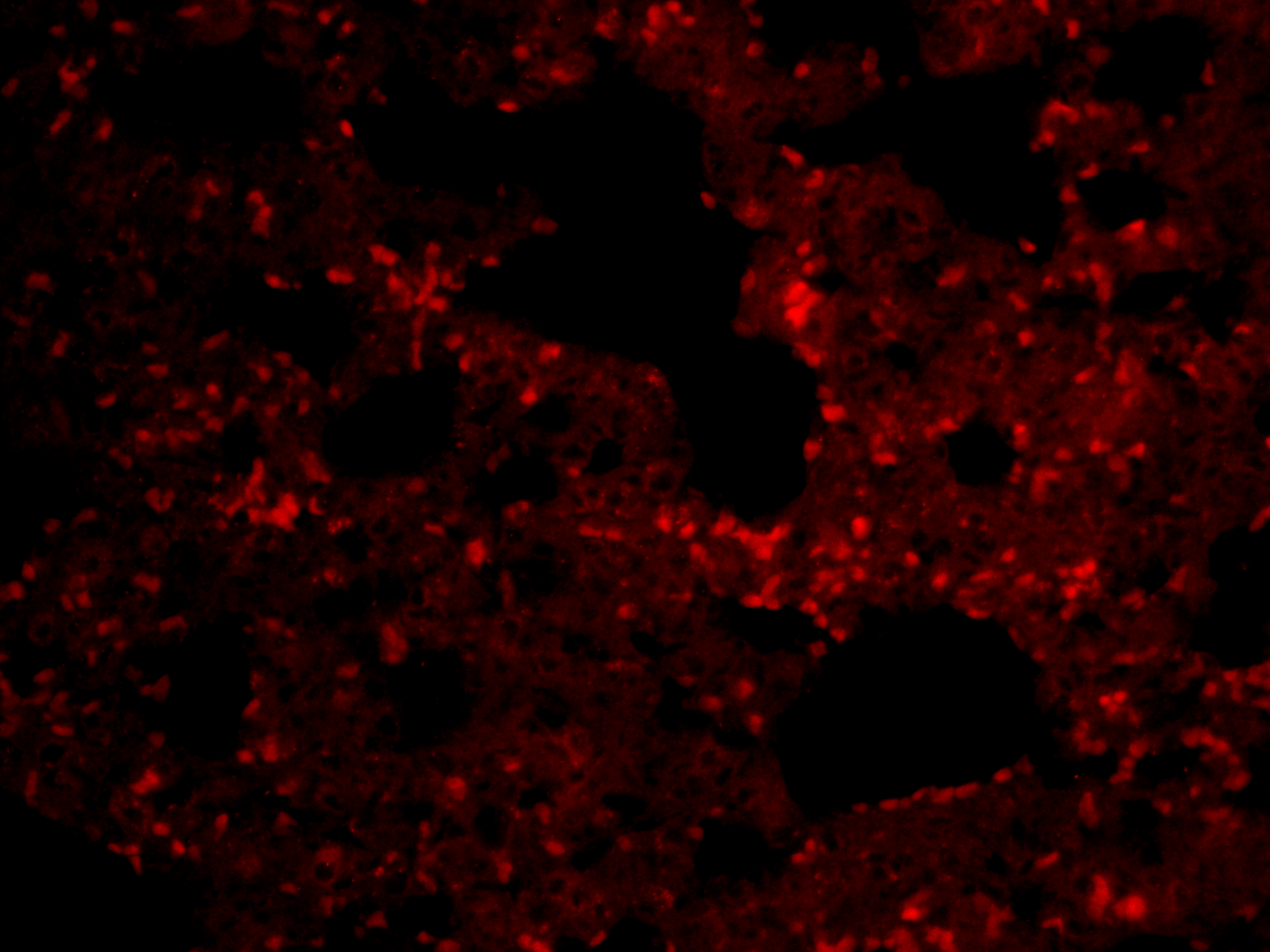

Supplement: Figure S4 — 15 photos comprising Figure 4. [file peerj-08-8254-s004.zip › Fig 4D.tif]

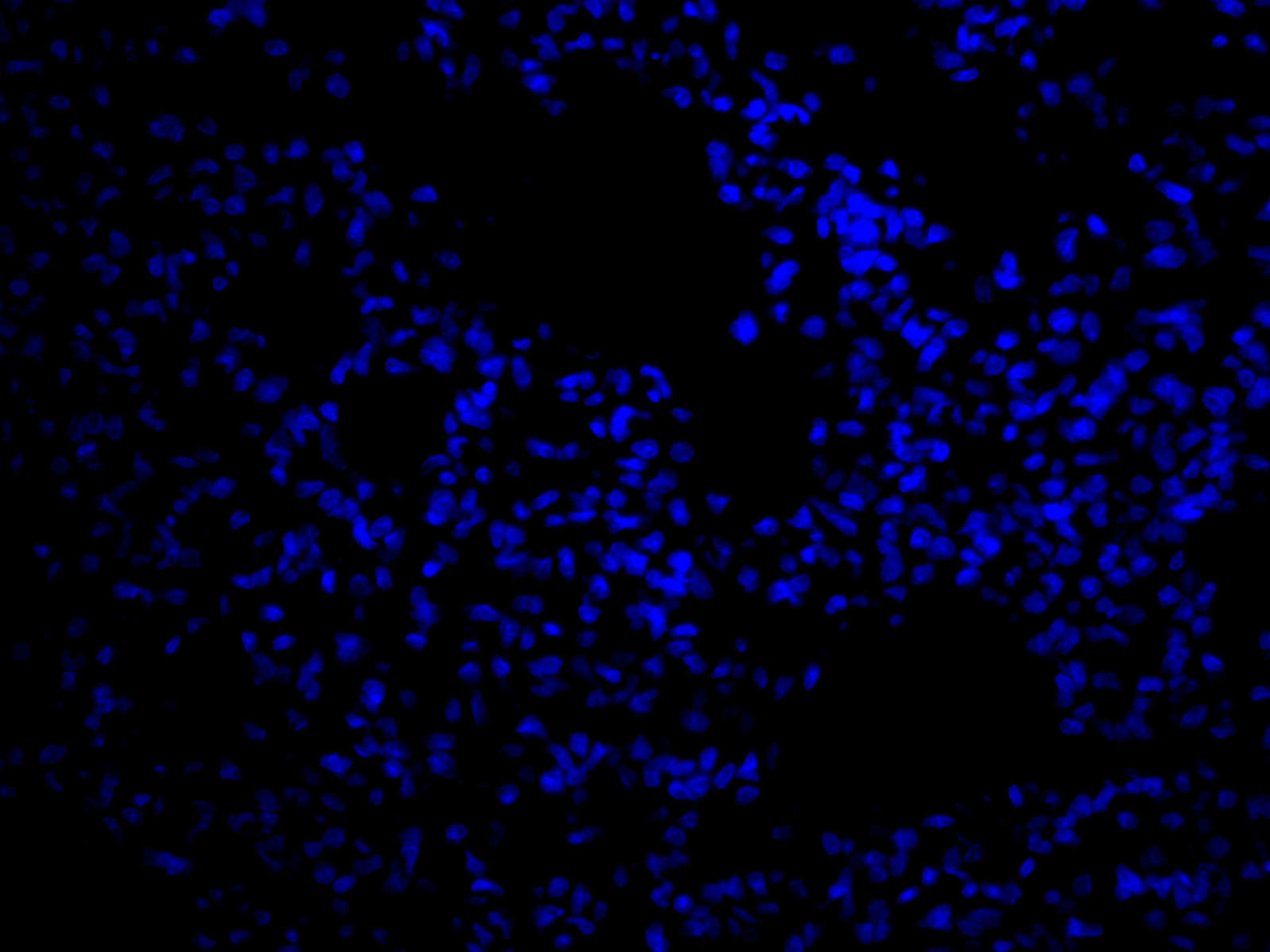

Supplement: Figure S4 — 15 photos comprising Figure 4. [file peerj-08-8254-s004.zip › Fig 4E.tif]

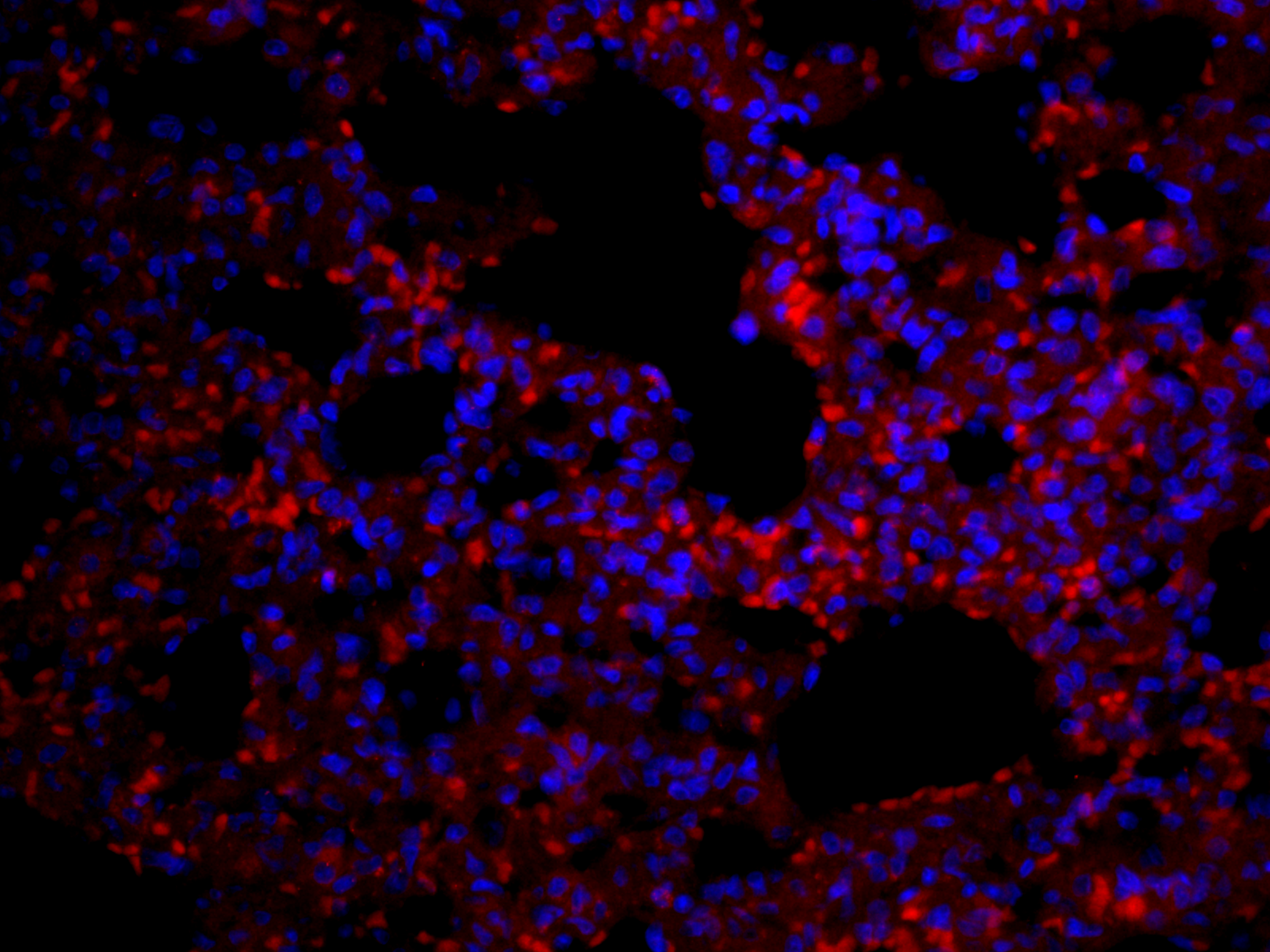

Supplement: Figure S4 — 15 photos comprising Figure 4. [file peerj-08-8254-s004.zip › Fig 4F.tif]

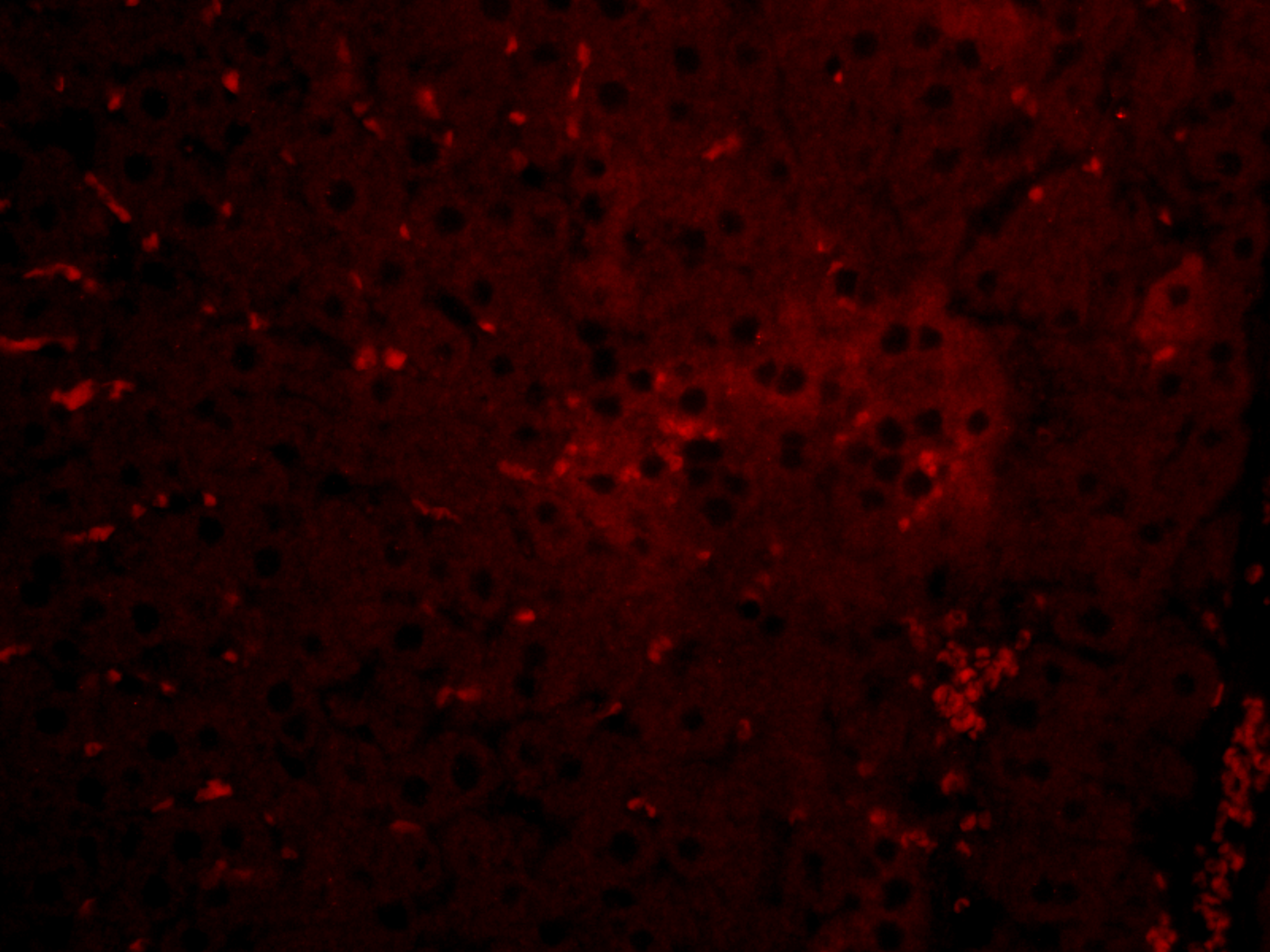

Supplement: Figure S4 — 15 photos comprising Figure 4. [file peerj-08-8254-s004.zip › Fig 4G.tif]

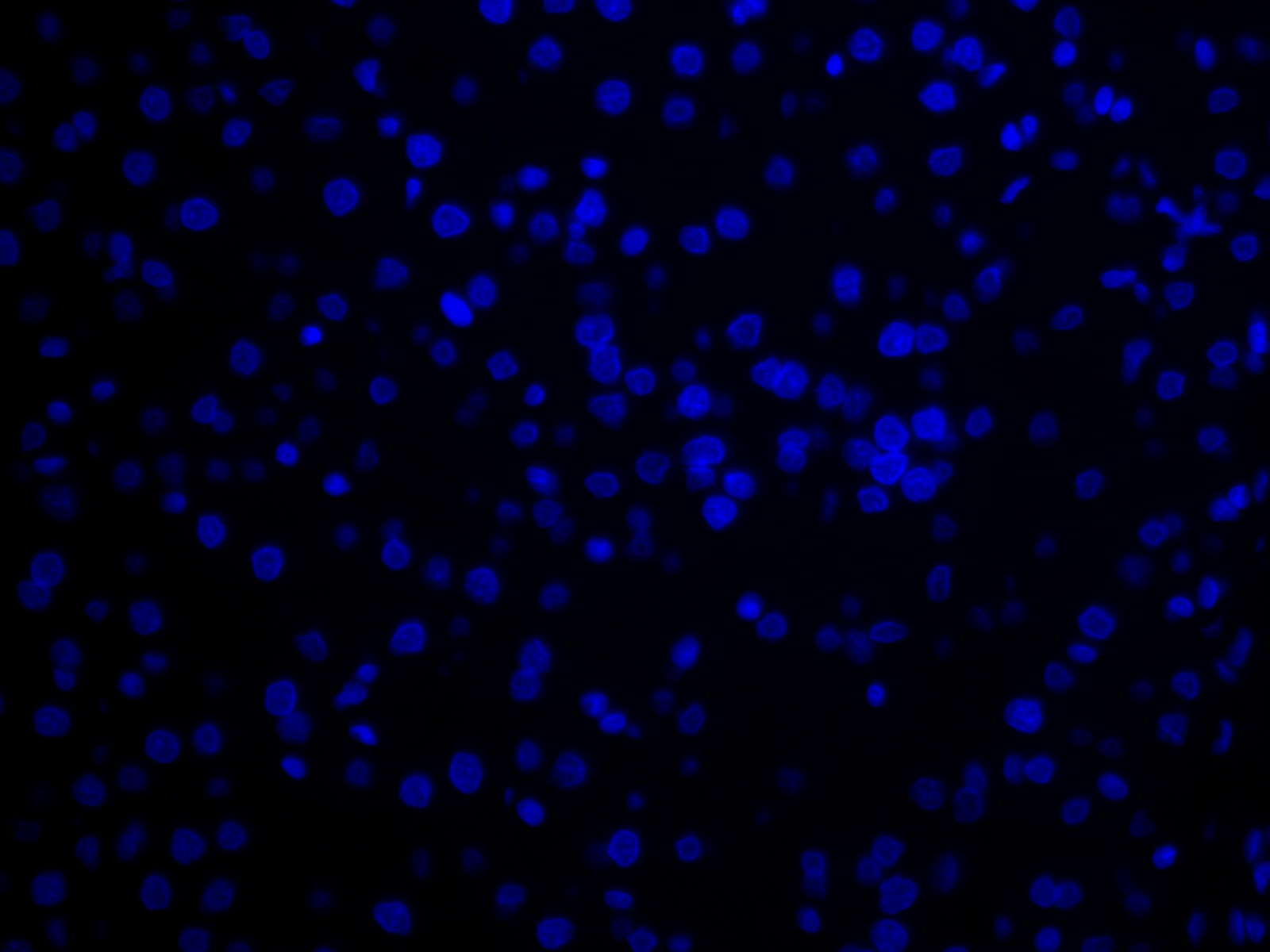

Supplement: Figure S4 — 15 photos comprising Figure 4. [file peerj-08-8254-s004.zip › Fig 4H.tif]

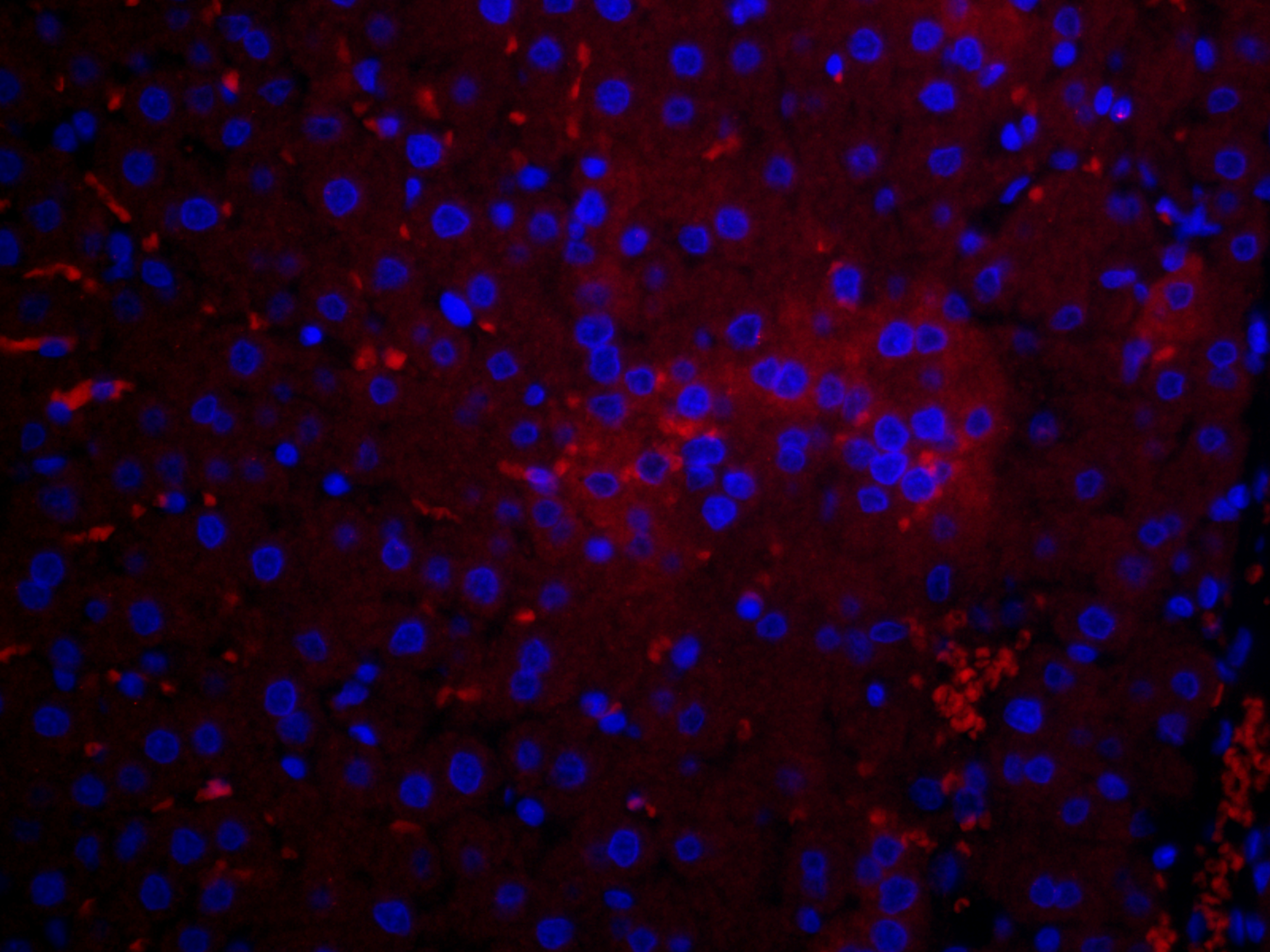

Supplement: Figure S4 — 15 photos comprising Figure 4. [file peerj-08-8254-s004.zip › Fig 4I.tif]

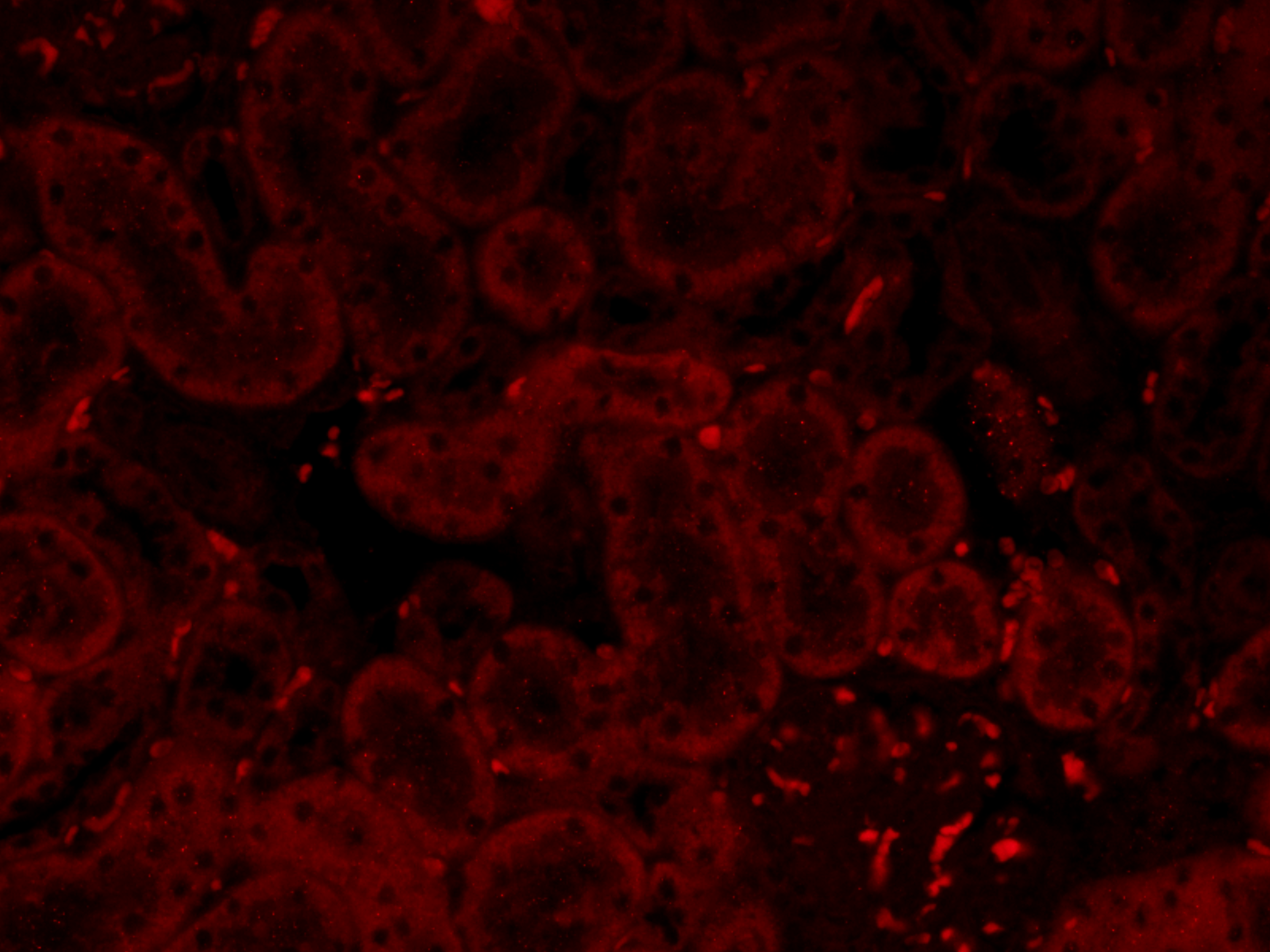

Supplement: Figure S4 — 15 photos comprising Figure 4. [file peerj-08-8254-s004.zip › Fig 4J.tif]

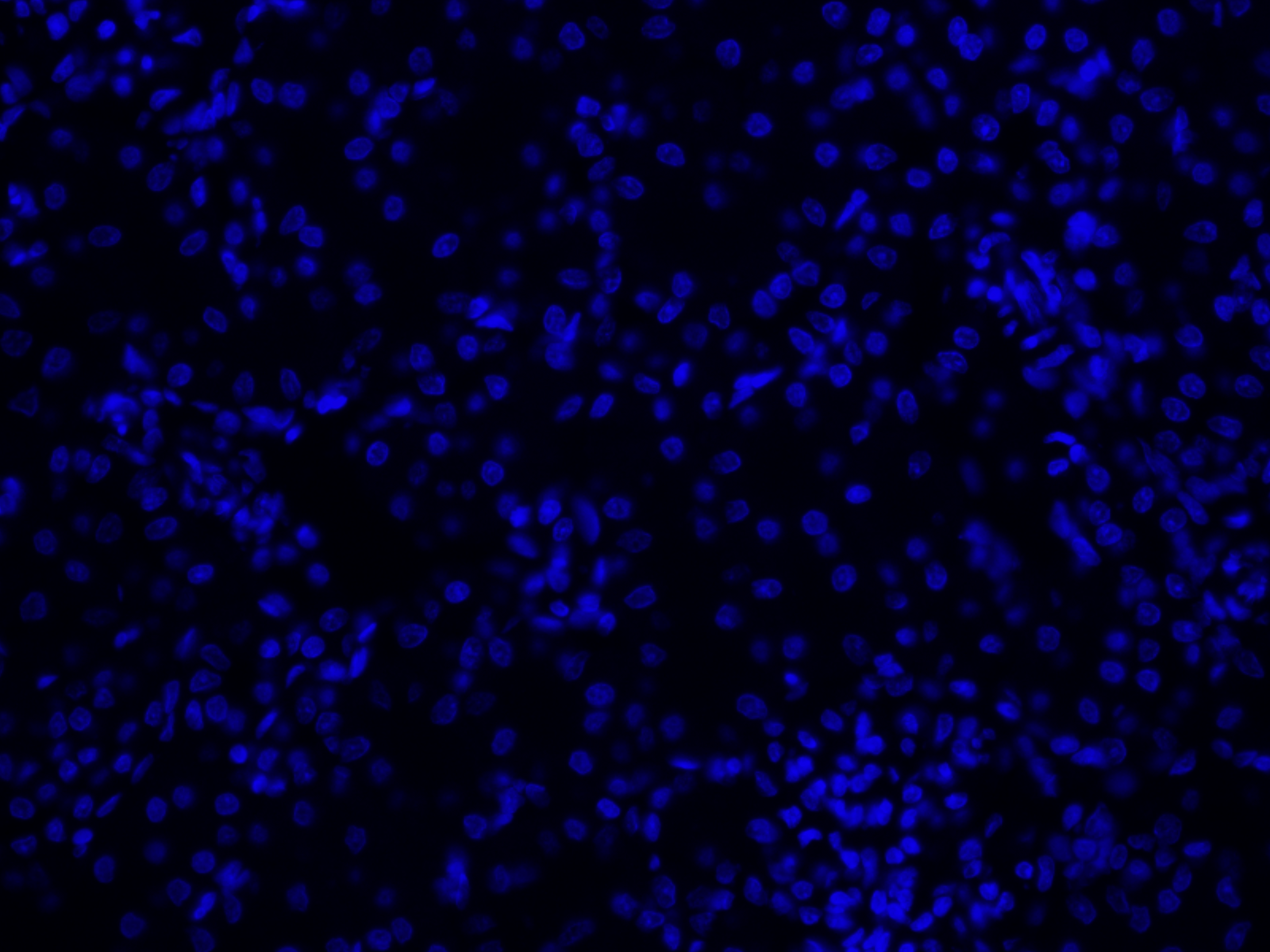

Supplement: Figure S4 — 15 photos comprising Figure 4. [file peerj-08-8254-s004.zip › Fig 4K.tif]

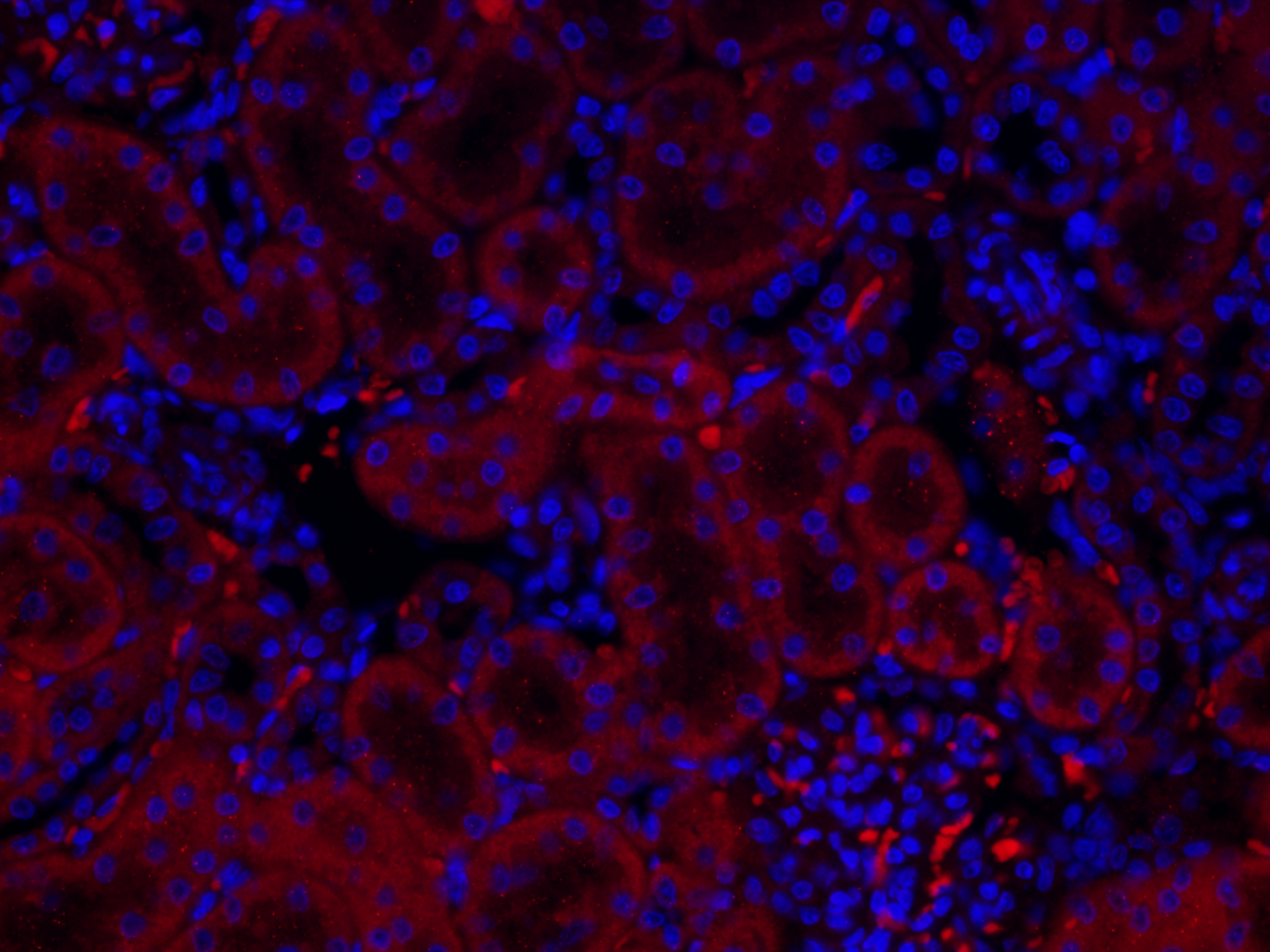

Supplement: Figure S4 — 15 photos comprising Figure 4. [file peerj-08-8254-s004.zip › Fig 4L.tif]

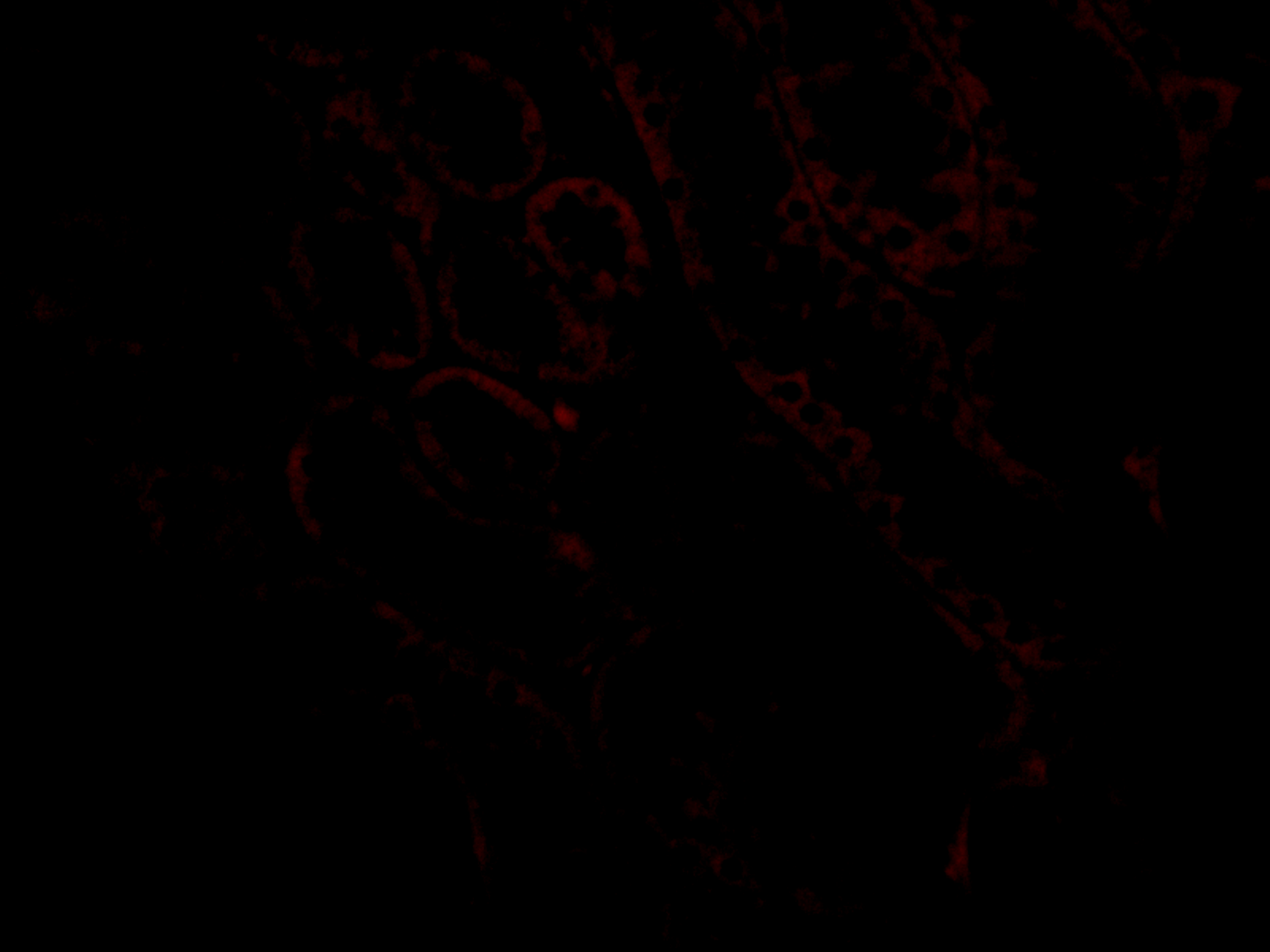

Supplement: Figure S4 — 15 photos comprising Figure 4. [file peerj-08-8254-s004.zip › Fig 4M.tif]

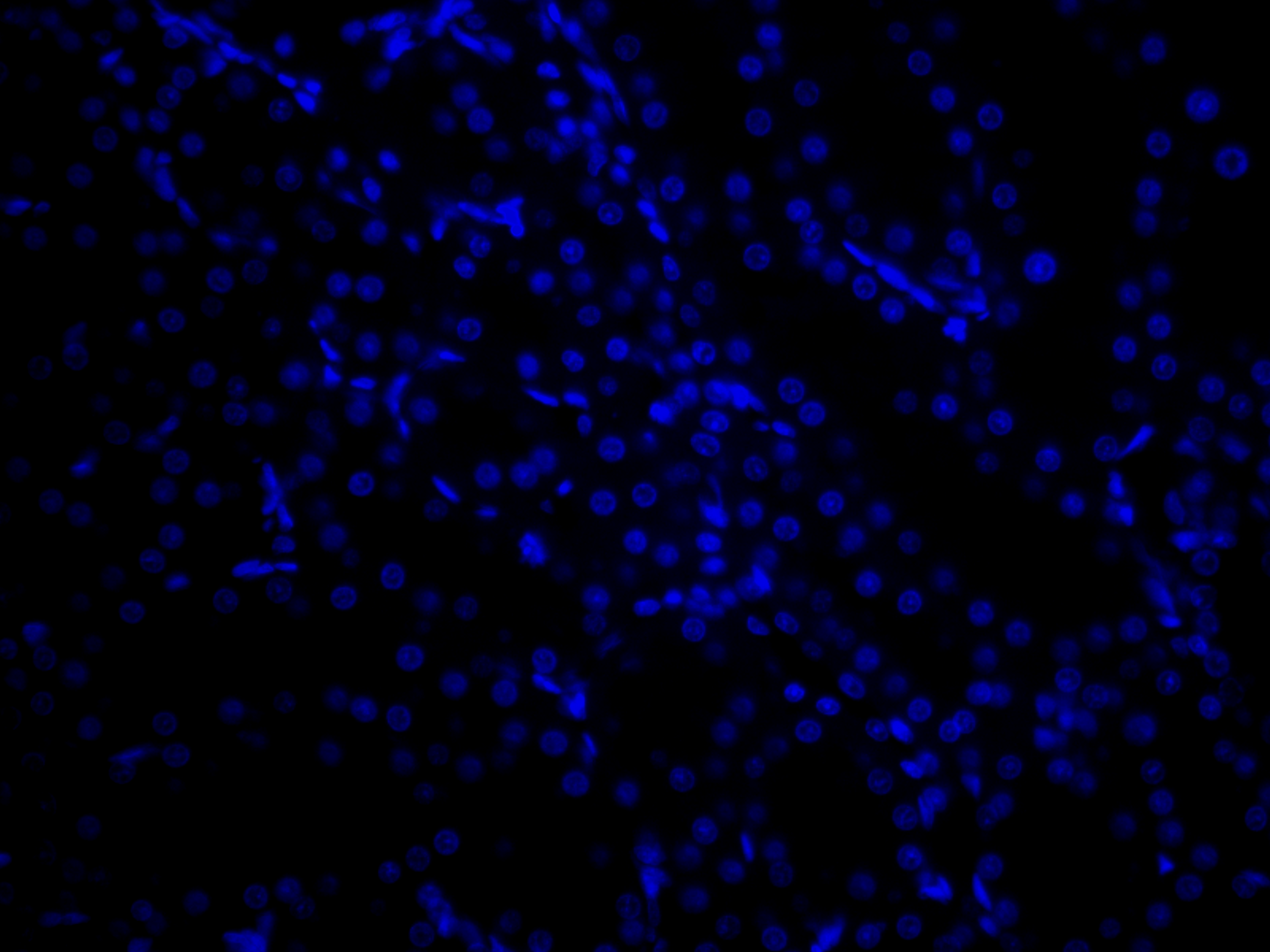

Supplement: Figure S4 — 15 photos comprising Figure 4. [file peerj-08-8254-s004.zip › Fig 4N.tif]

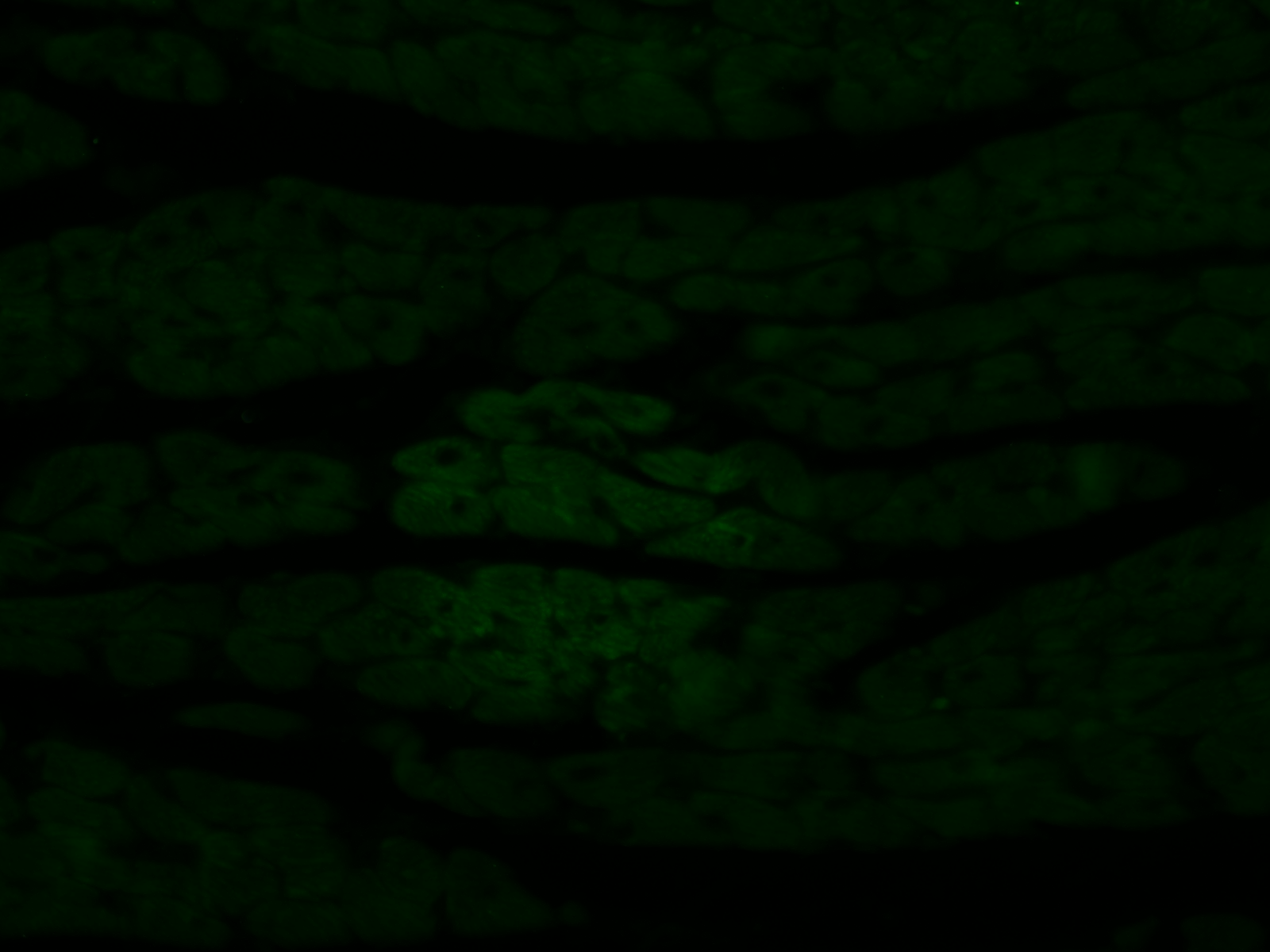

Supplement: Figure S5 — 8 photos comprising Figure 5. [file peerj-08-8254-s005.zip › Figure 5B.tif]

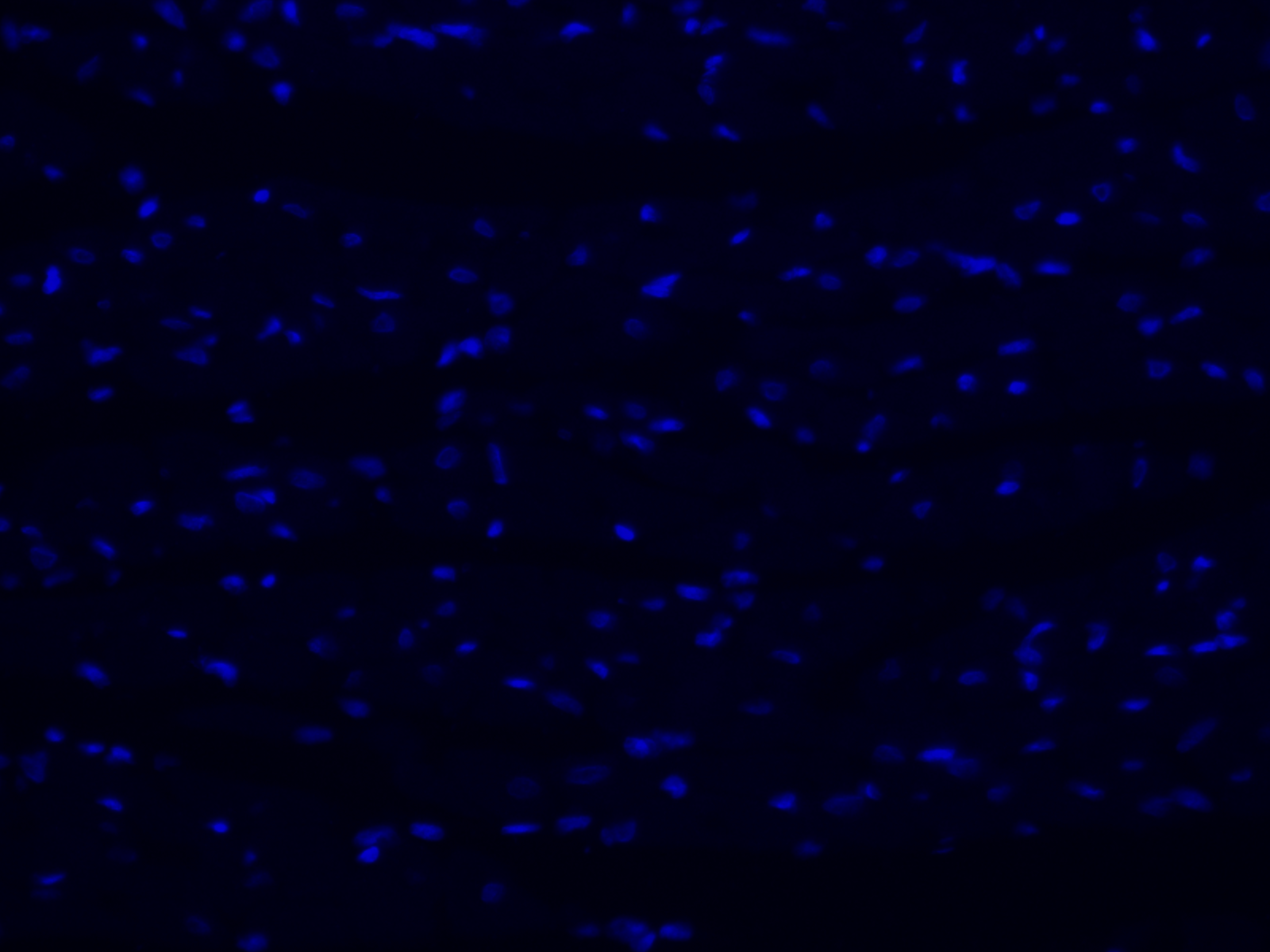

Supplement: Figure S5 — 8 photos comprising Figure 5. [file peerj-08-8254-s005.zip › Figure 5C.tif]

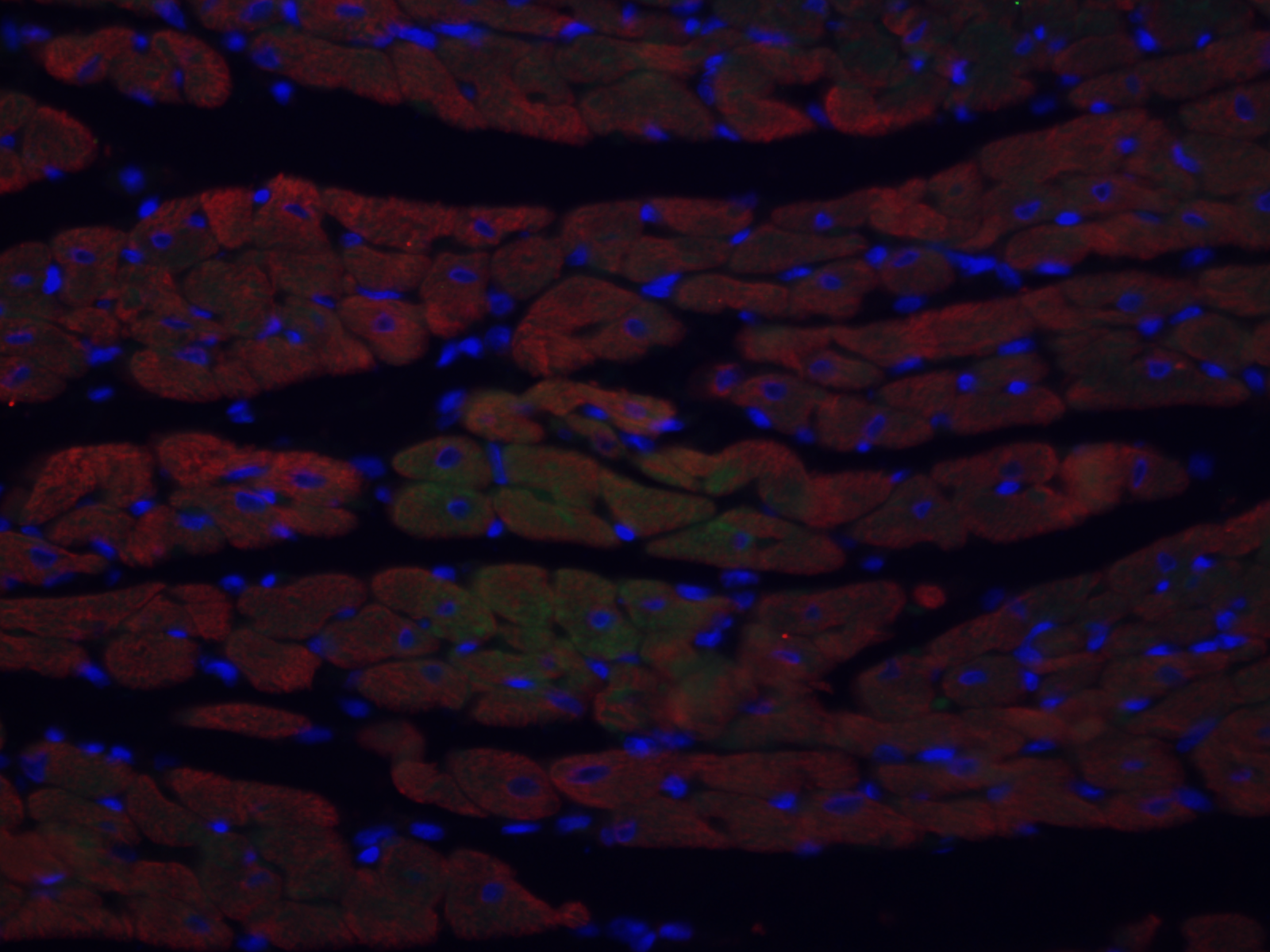

Supplement: Figure S5 — 8 photos comprising Figure 5. [file peerj-08-8254-s005.zip › Figure 5D.tif]

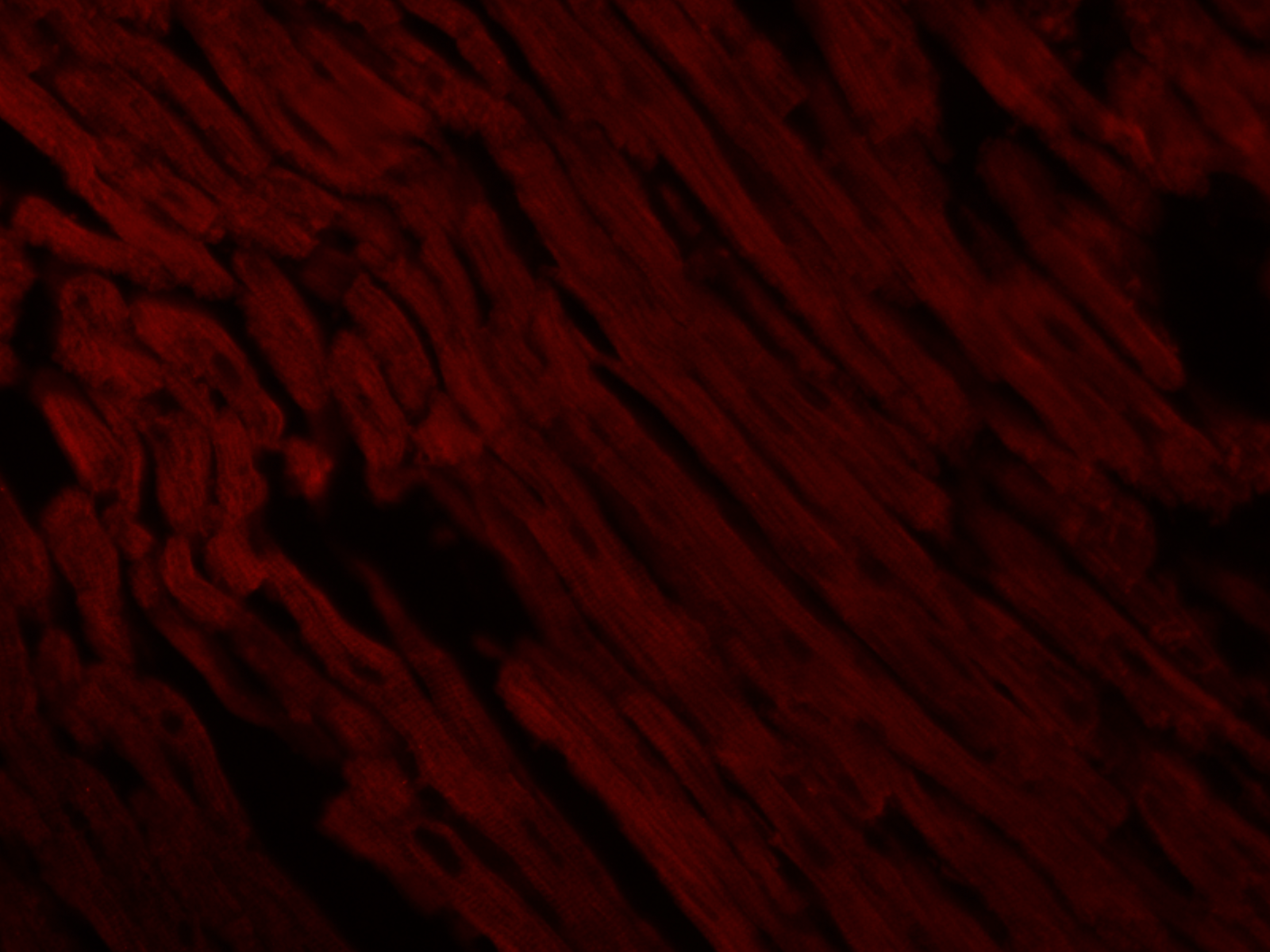

Supplement: Figure S5 — 8 photos comprising Figure 5. [file peerj-08-8254-s005.zip › Figure 5E.tif]

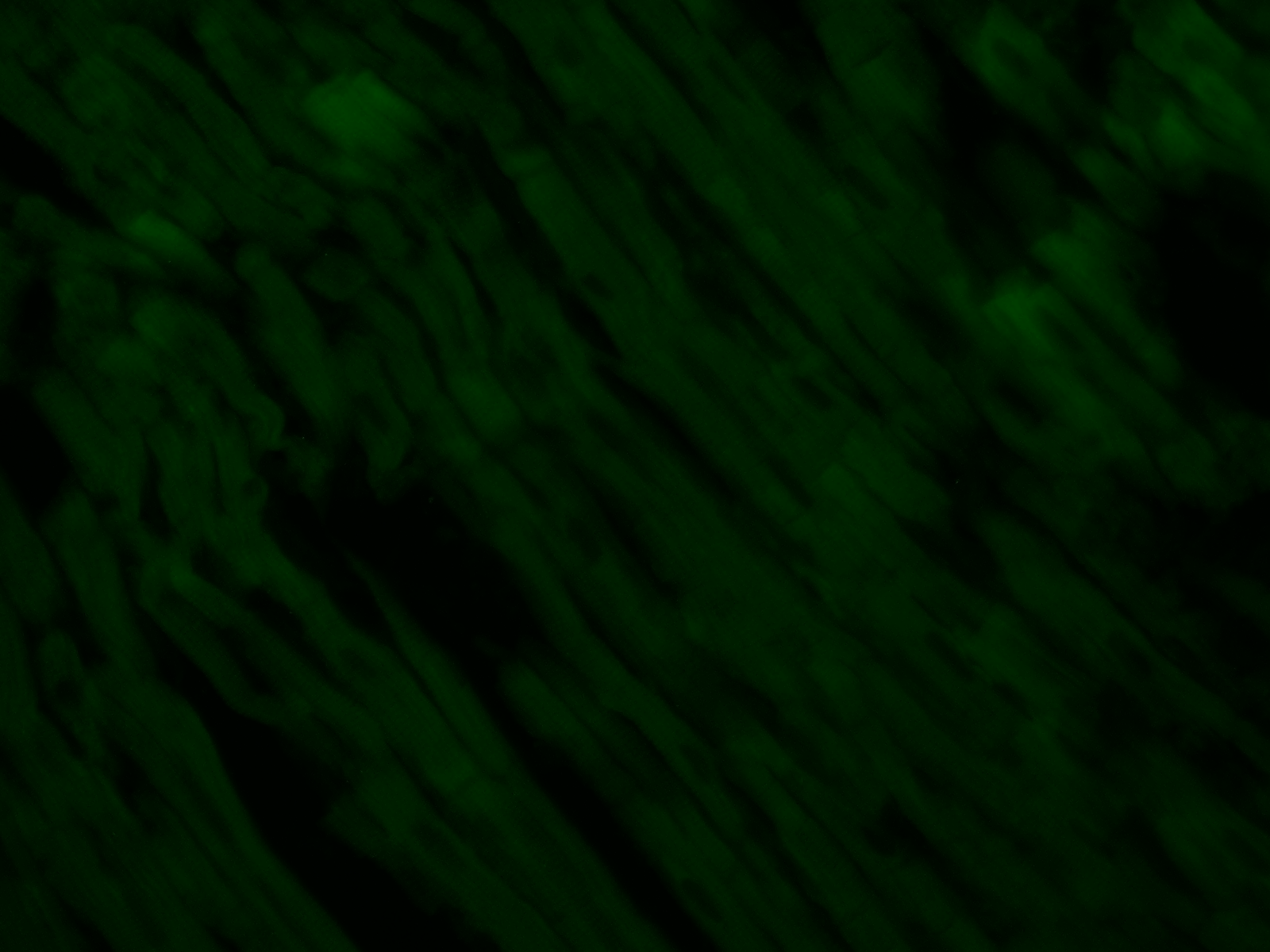

Supplement: Figure S5 — 8 photos comprising Figure 5. [file peerj-08-8254-s005.zip › Figure 5F.tif]

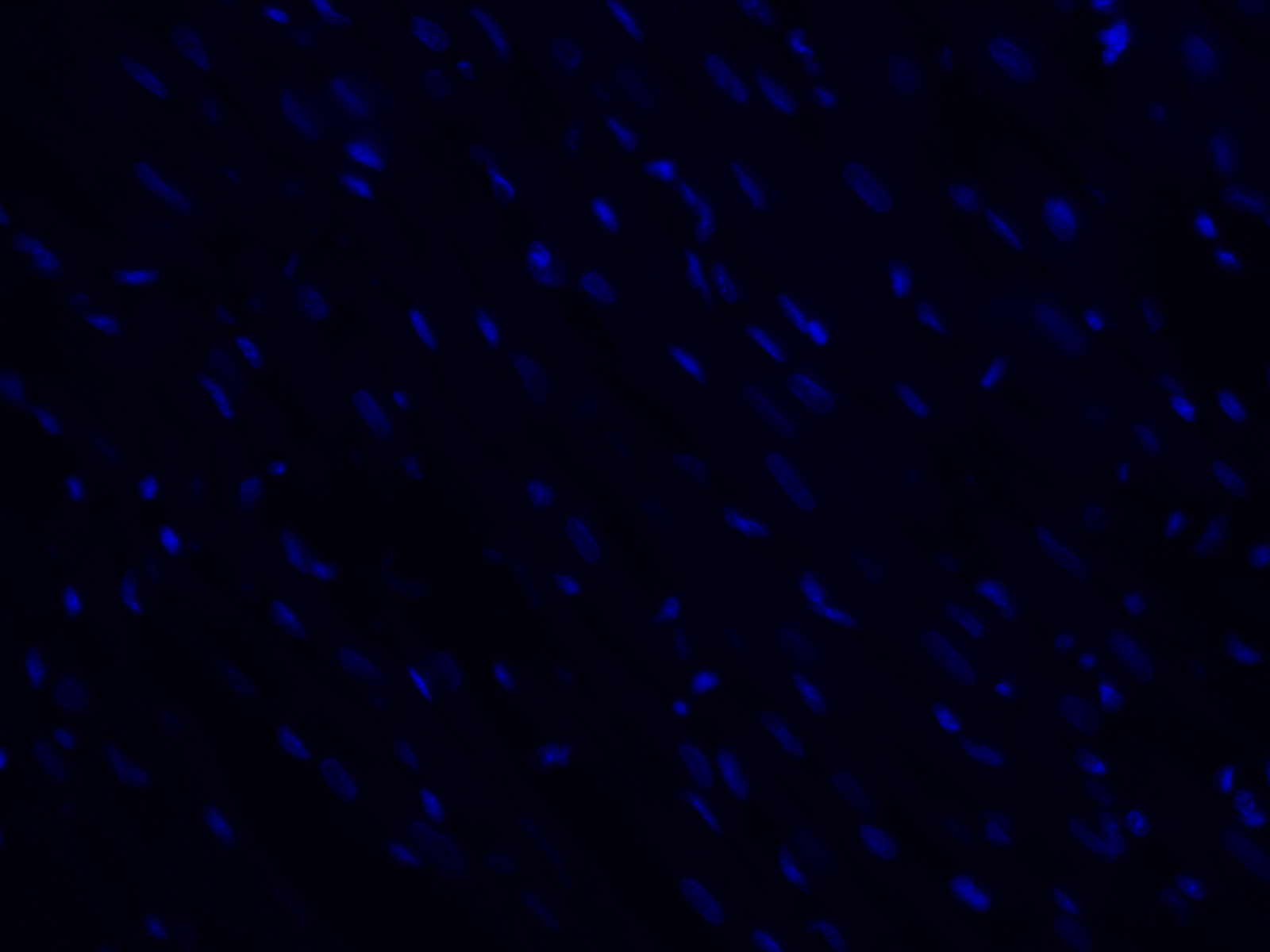

Supplement: Figure S5 — 8 photos comprising Figure 5. [file peerj-08-8254-s005.zip › G.tif]

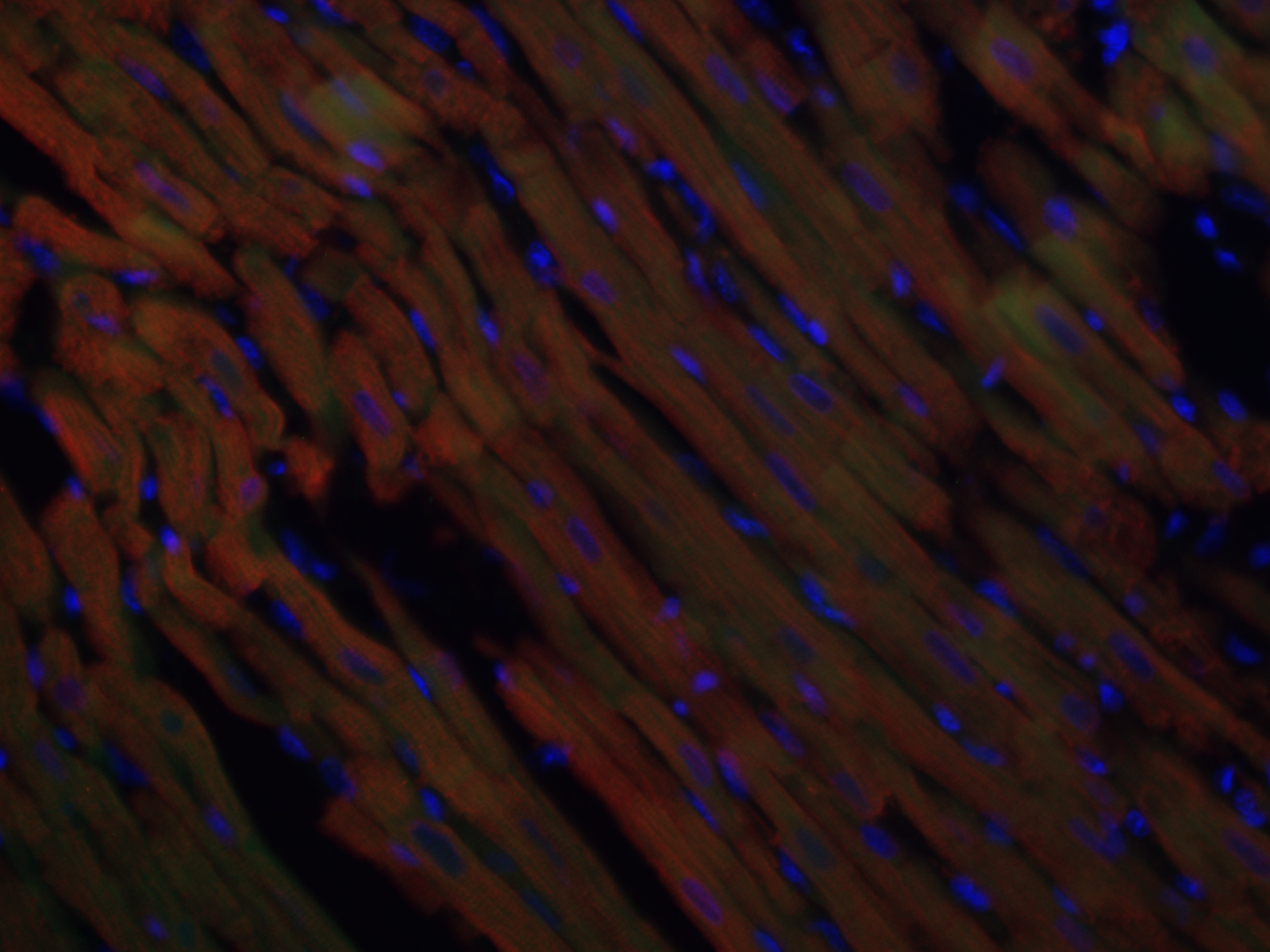

Supplement: Figure S5 — 8 photos comprising Figure 5. [file peerj-08-8254-s005.zip › H.tif]

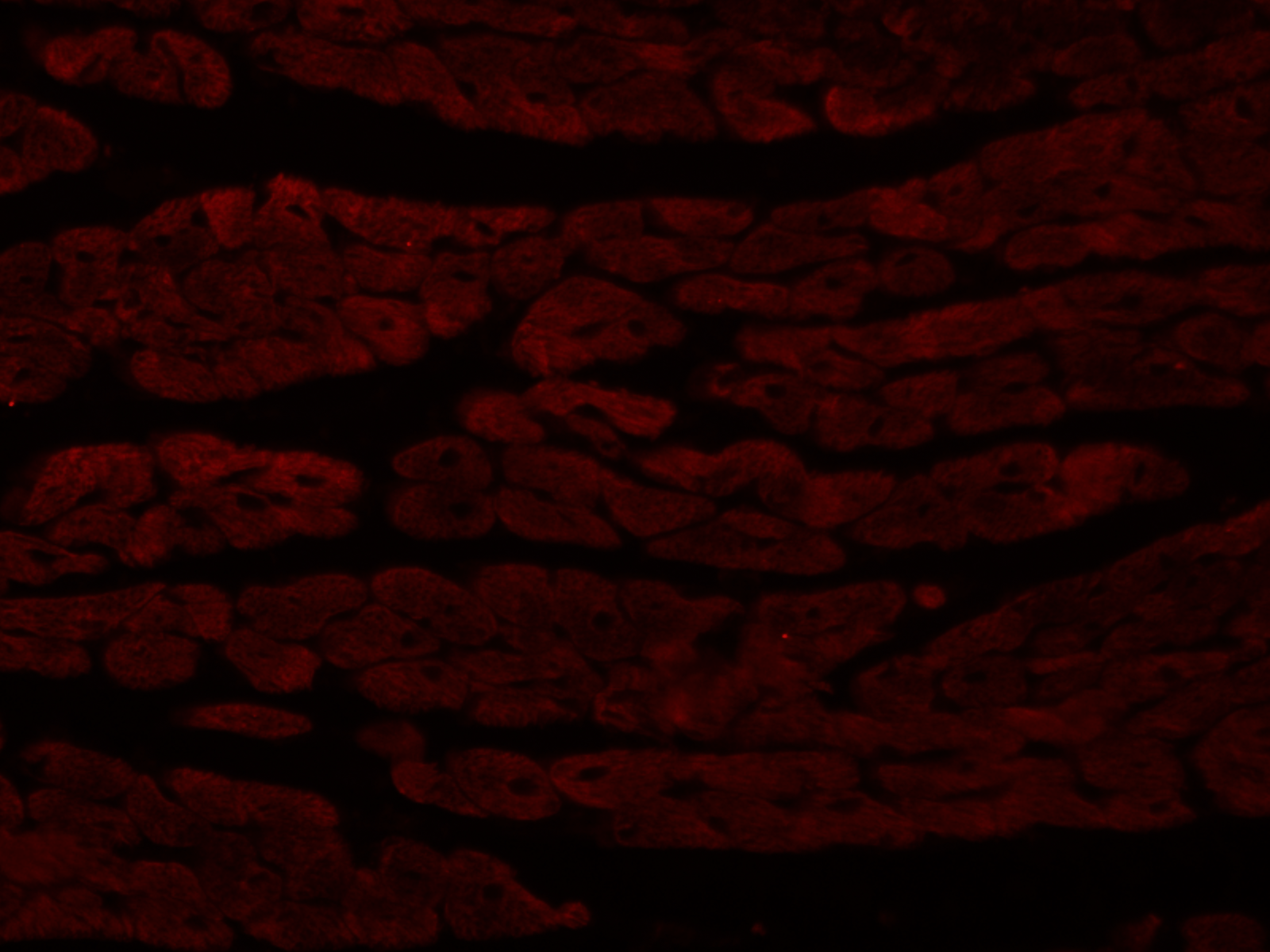

Supplement: Figure S5 — 8 photos comprising Figure 5. [file peerj-08-8254-s005.zip › Figure 5A.tif]

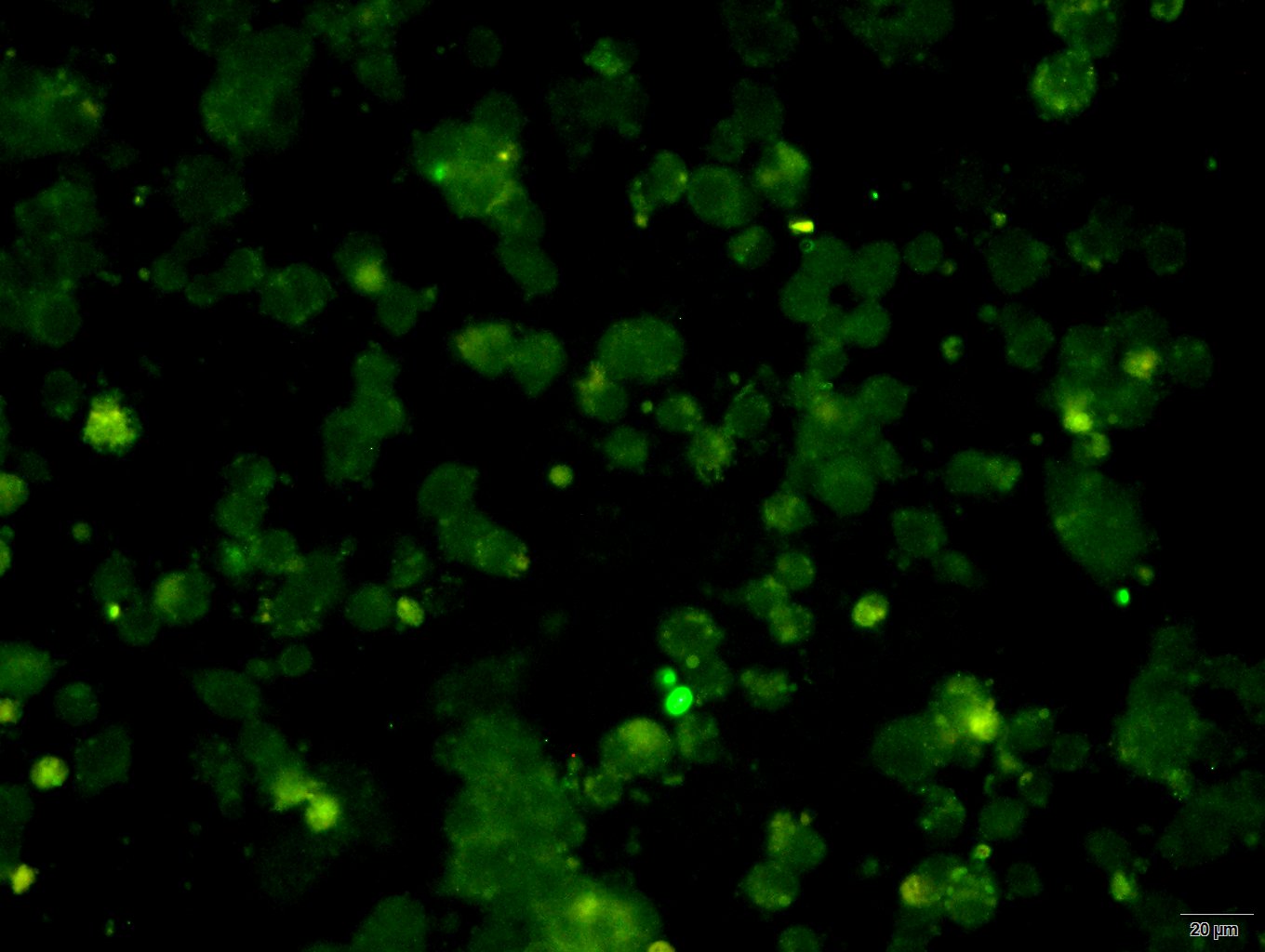

Supplement: Figure S7 — 21 photos comprising Figure 7. [file peerj-08-8254-s007.zip › Fig 7p.jpg]

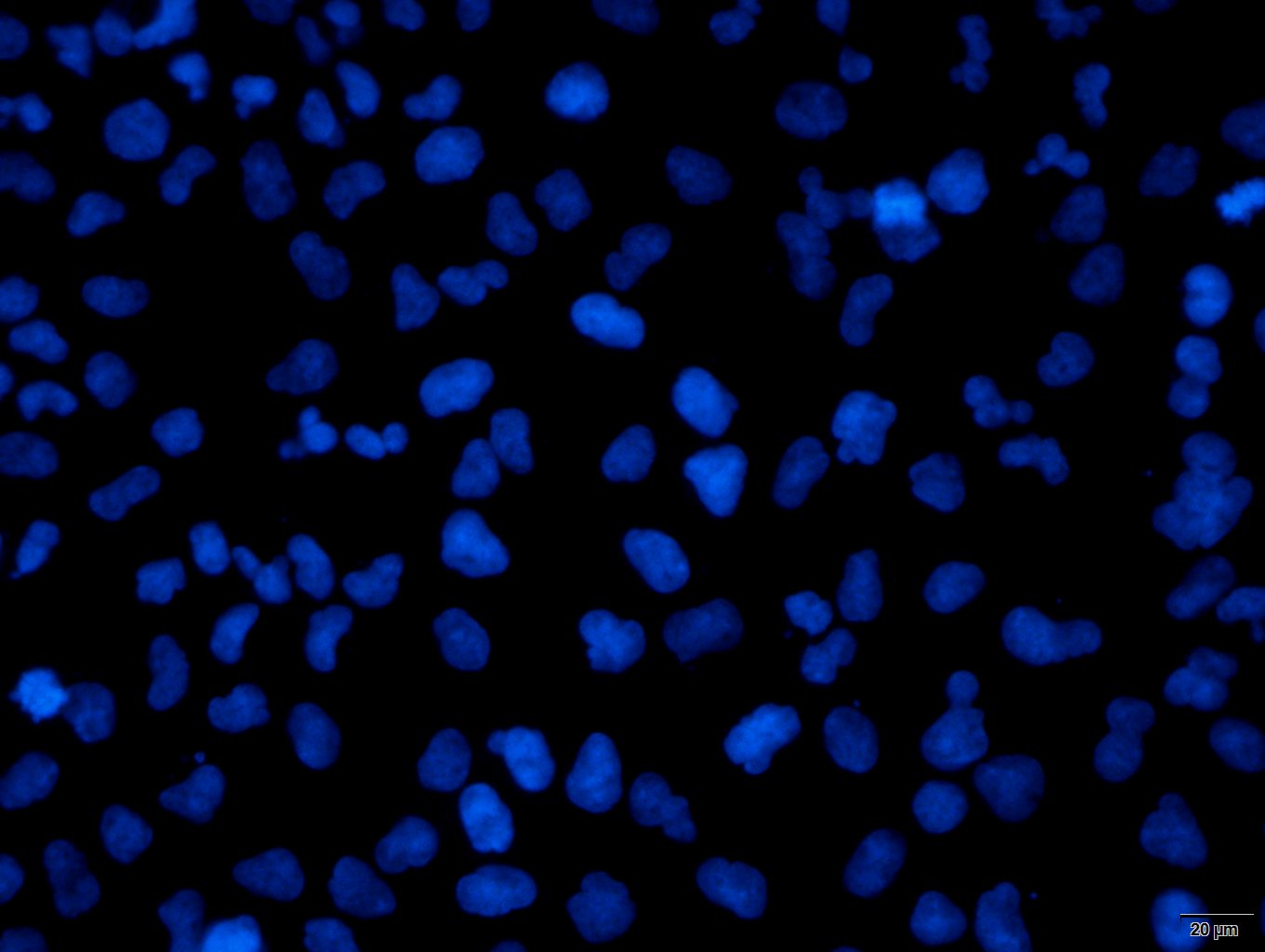

Supplement: Figure S7 — 21 photos comprising Figure 7. [file peerj-08-8254-s007.zip › Fig 7b.jpg]

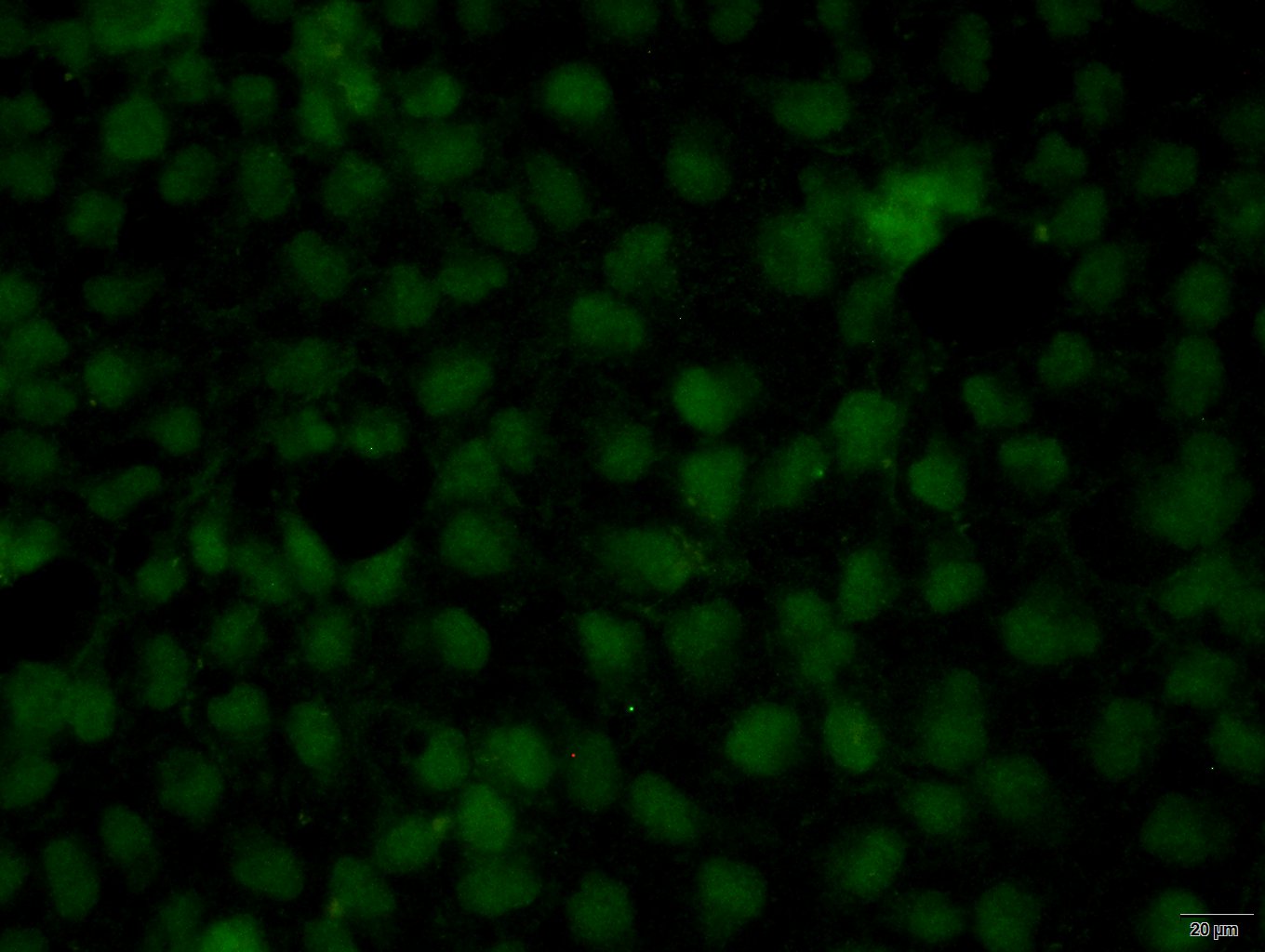

Supplement: Figure S7 — 21 photos comprising Figure 7. [file peerj-08-8254-s007.zip › Fig 7a.jpg]

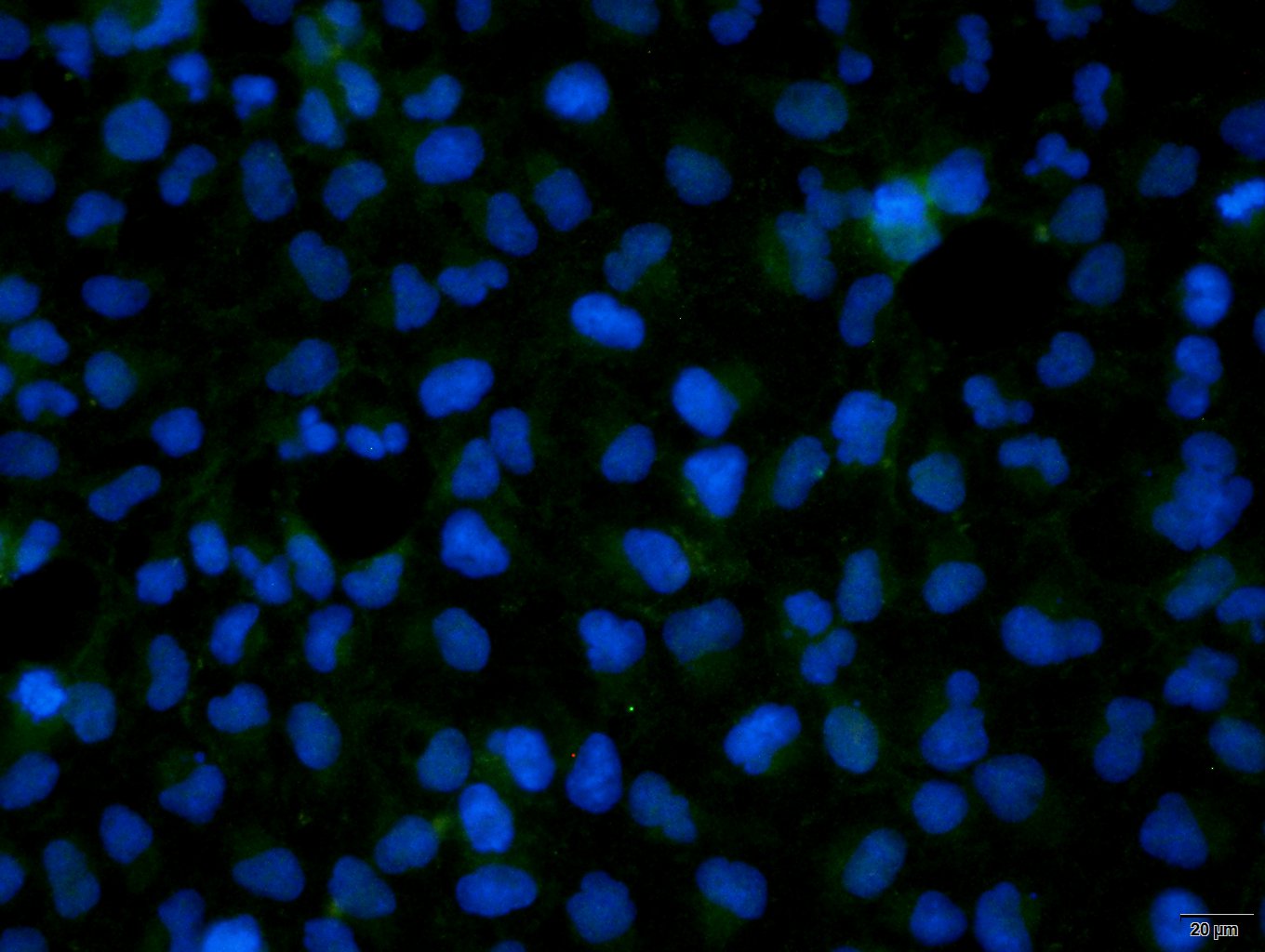

Supplement: Figure S7 — 21 photos comprising Figure 7. [file peerj-08-8254-s007.zip › Fig 7c.jpg]

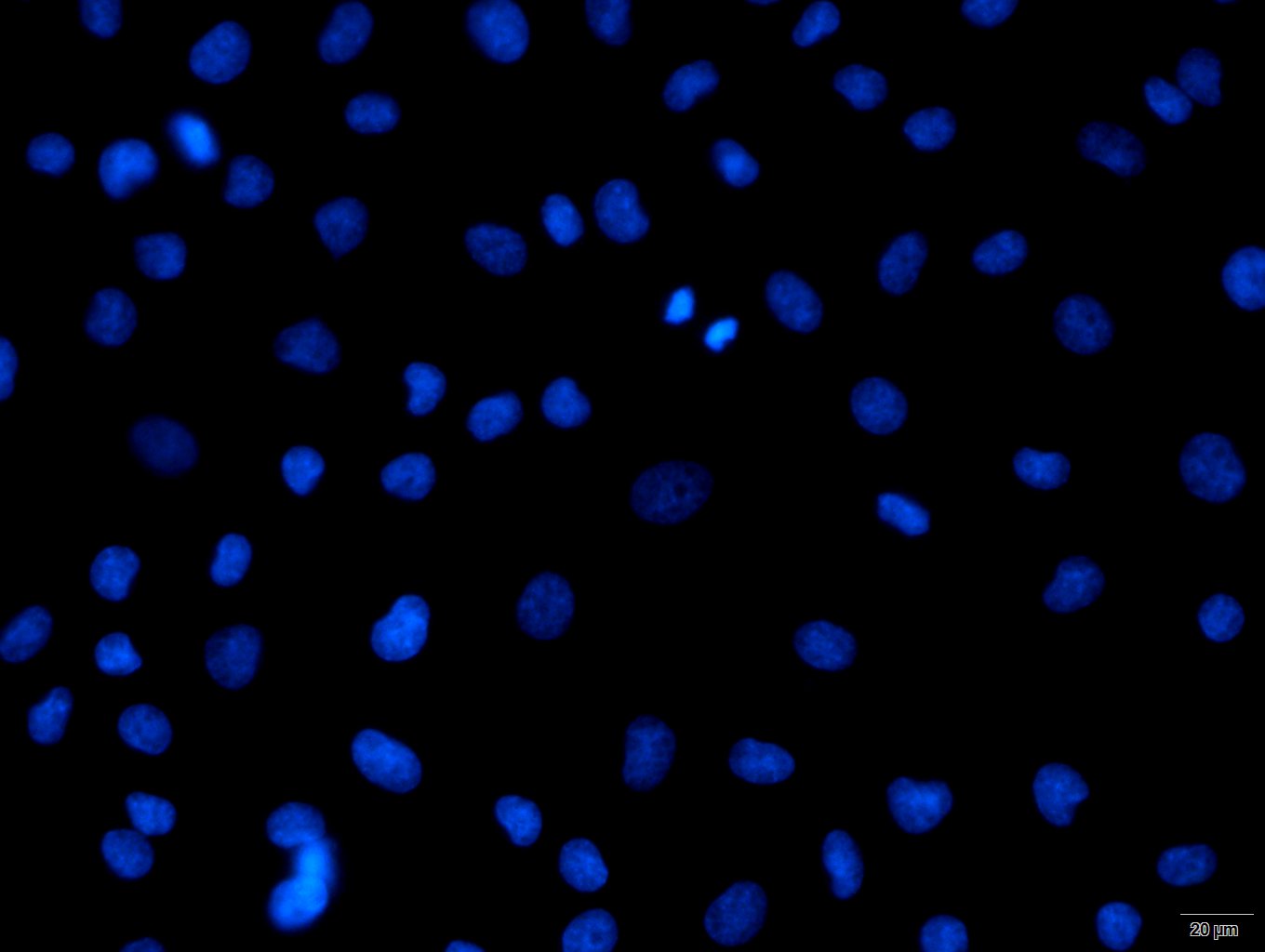

Supplement: Figure S7 — 21 photos comprising Figure 7. [file peerj-08-8254-s007.zip › Fig 7e.jpg]

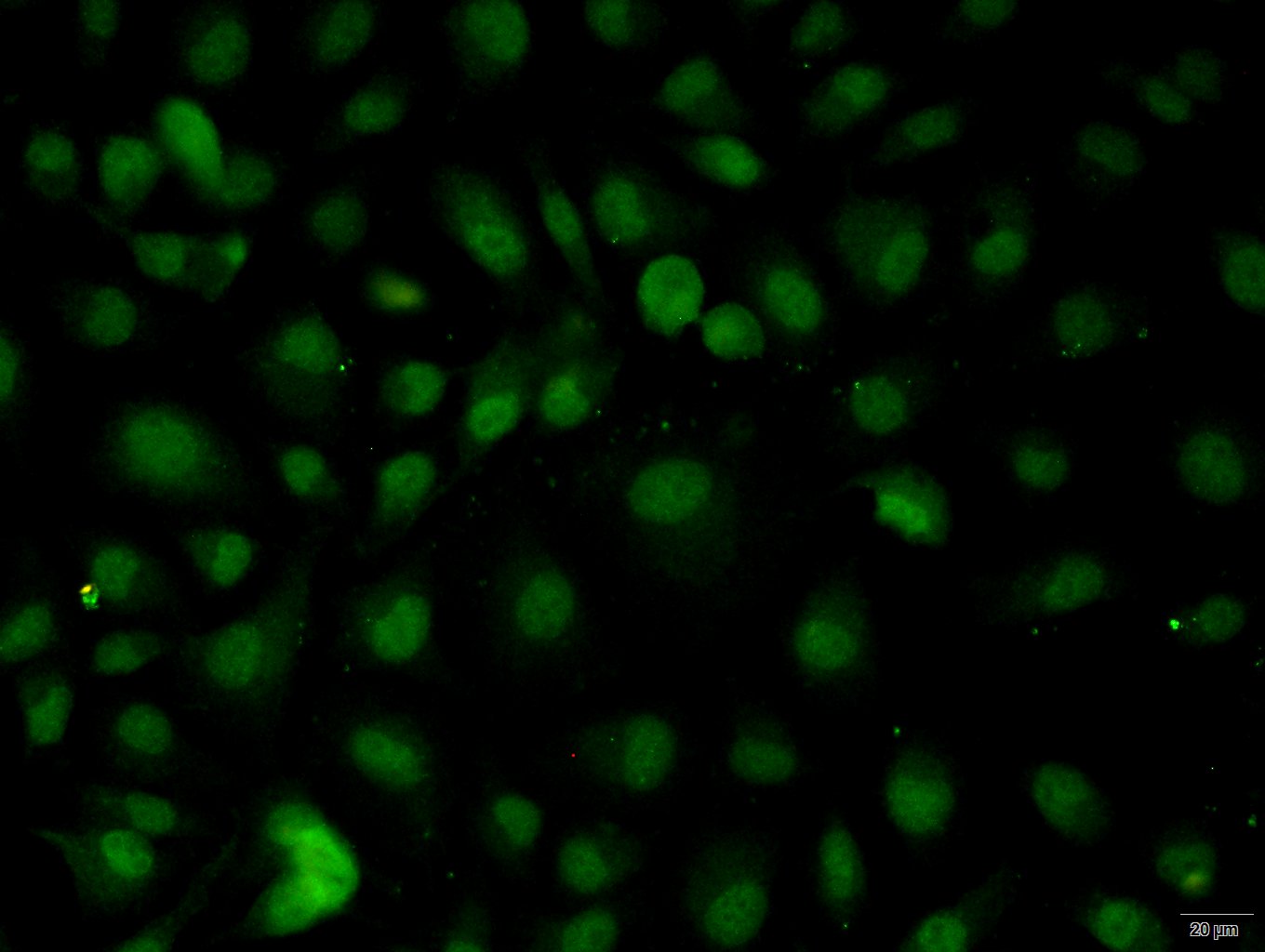

Supplement: Figure S7 — 21 photos comprising Figure 7. [file peerj-08-8254-s007.zip › Fig 7d.jpg]

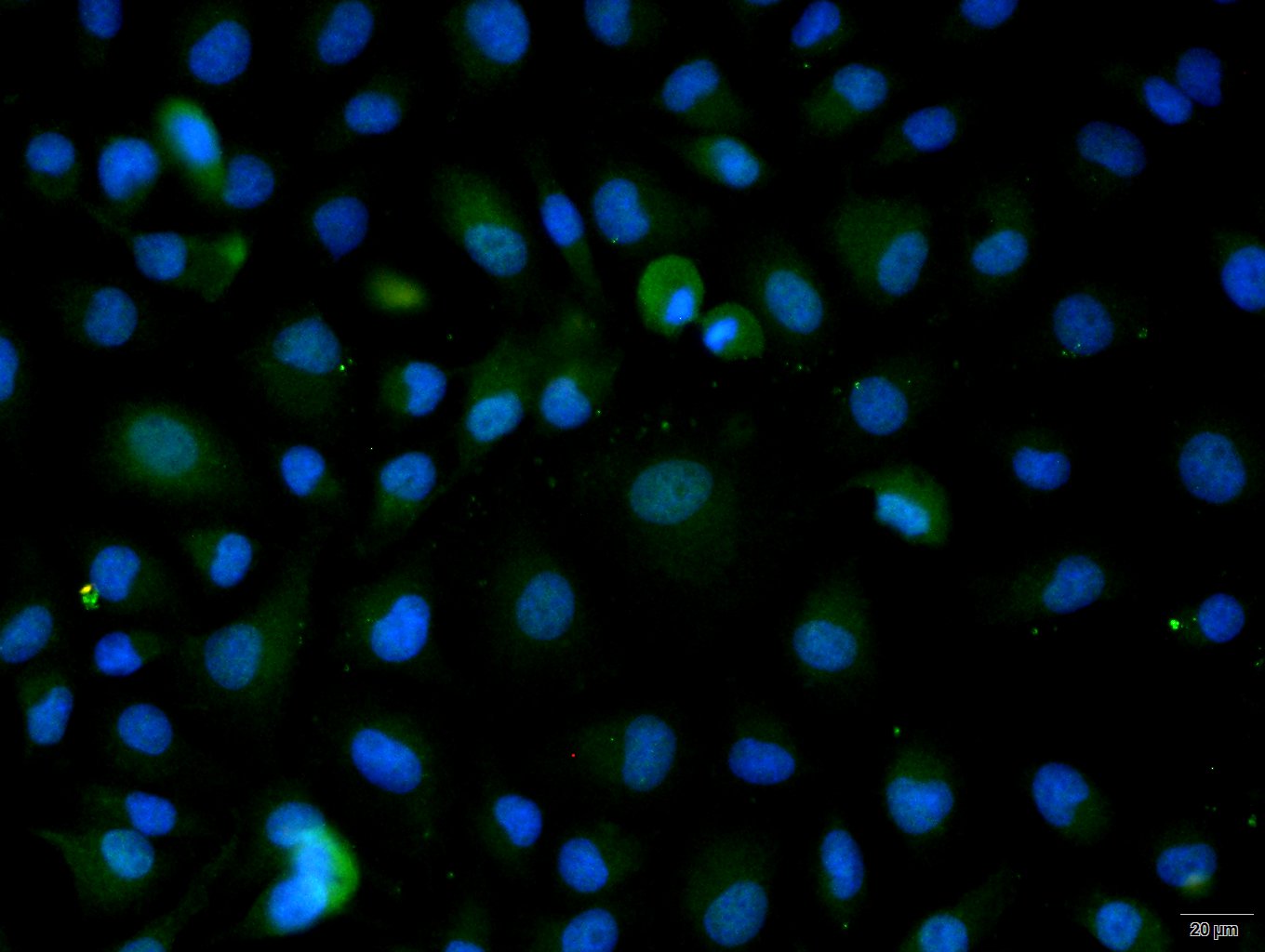

Supplement: Figure S7 — 21 photos comprising Figure 7. [file peerj-08-8254-s007.zip › Fig 7f.jpg]

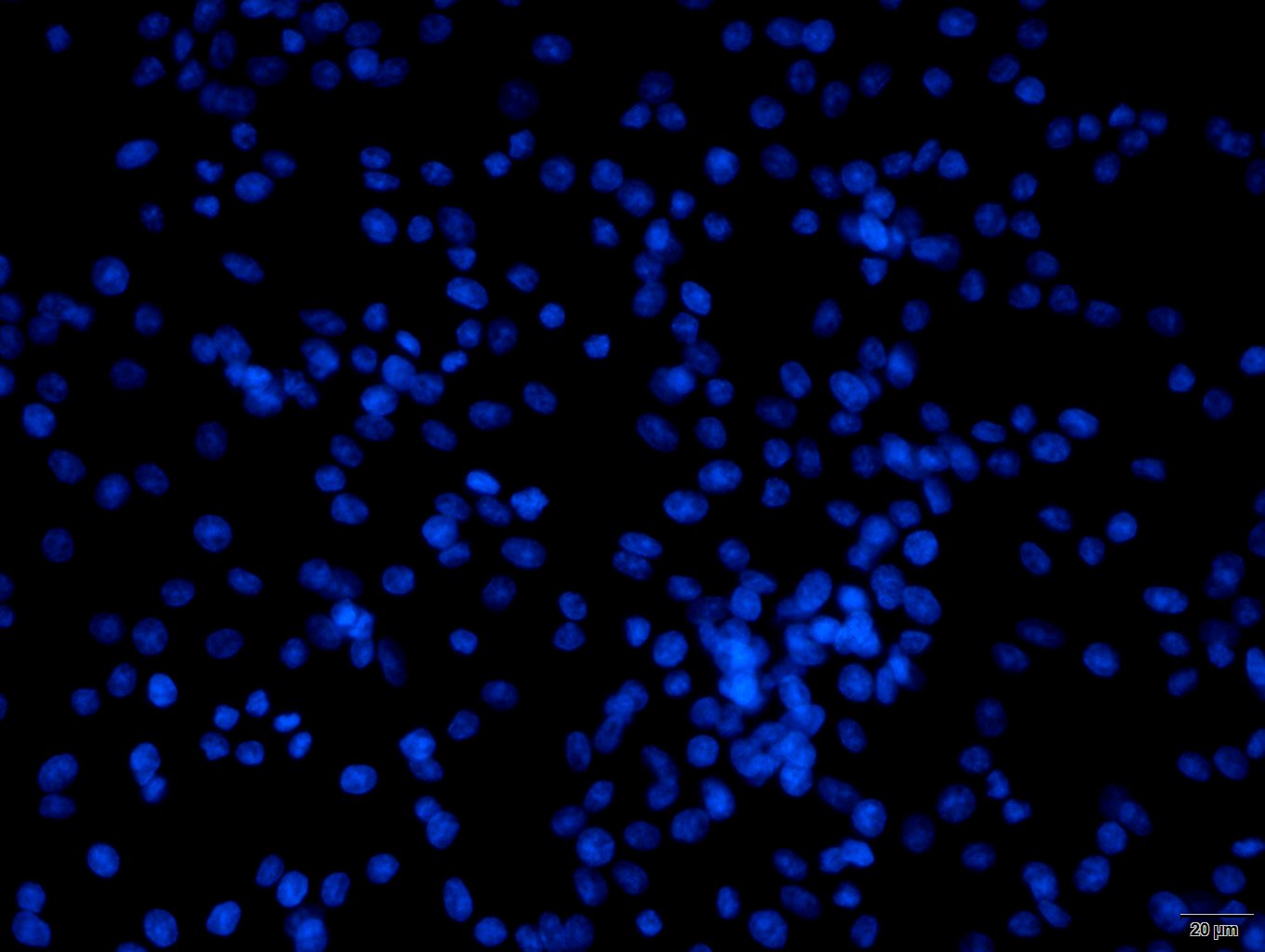

Supplement: Figure S7 — 21 photos comprising Figure 7. [file peerj-08-8254-s007.zip › Fig 7h.jpg]

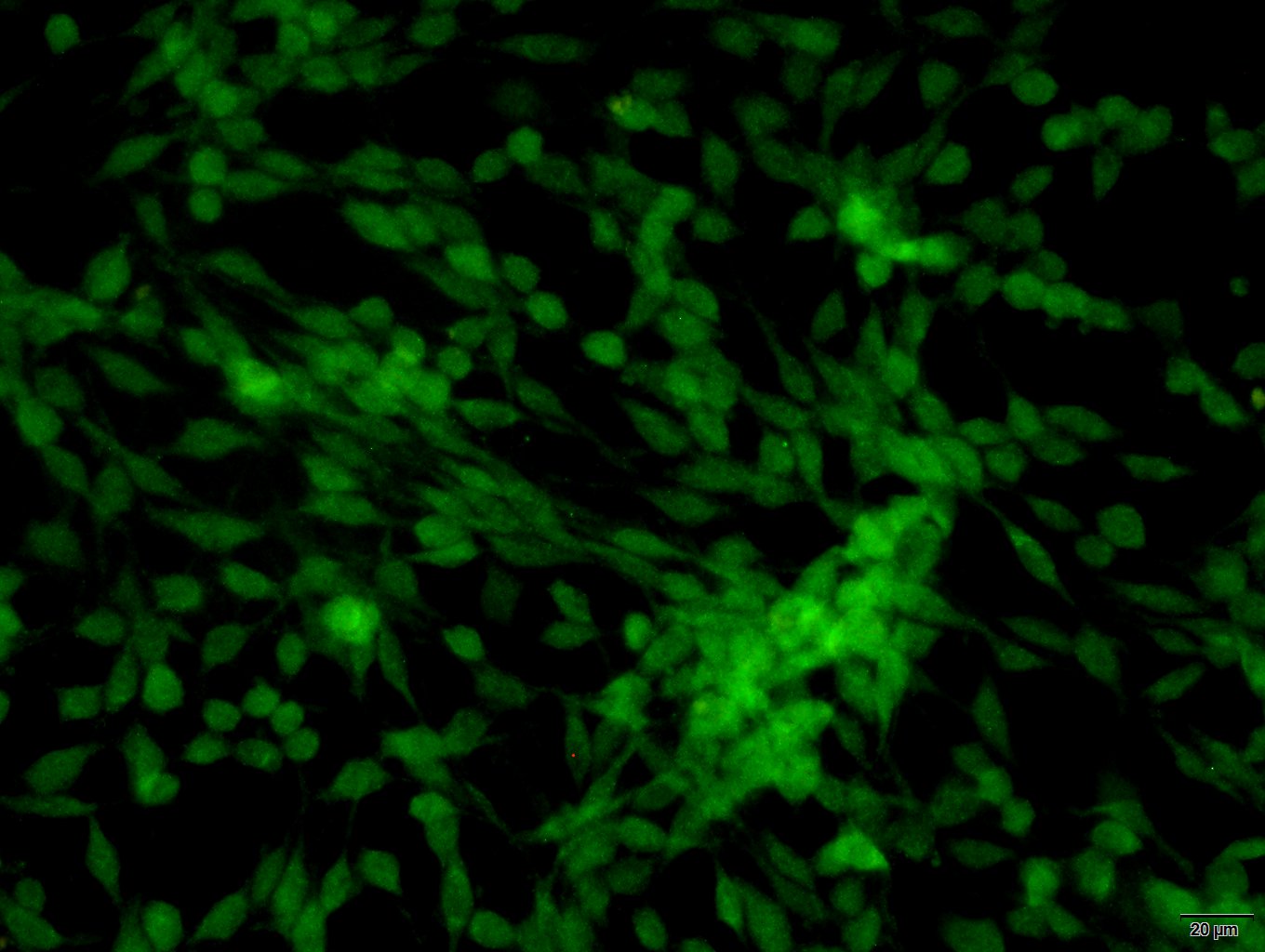

Supplement: Figure S7 — 21 photos comprising Figure 7. [file peerj-08-8254-s007.zip › Fig 7g.jpg]

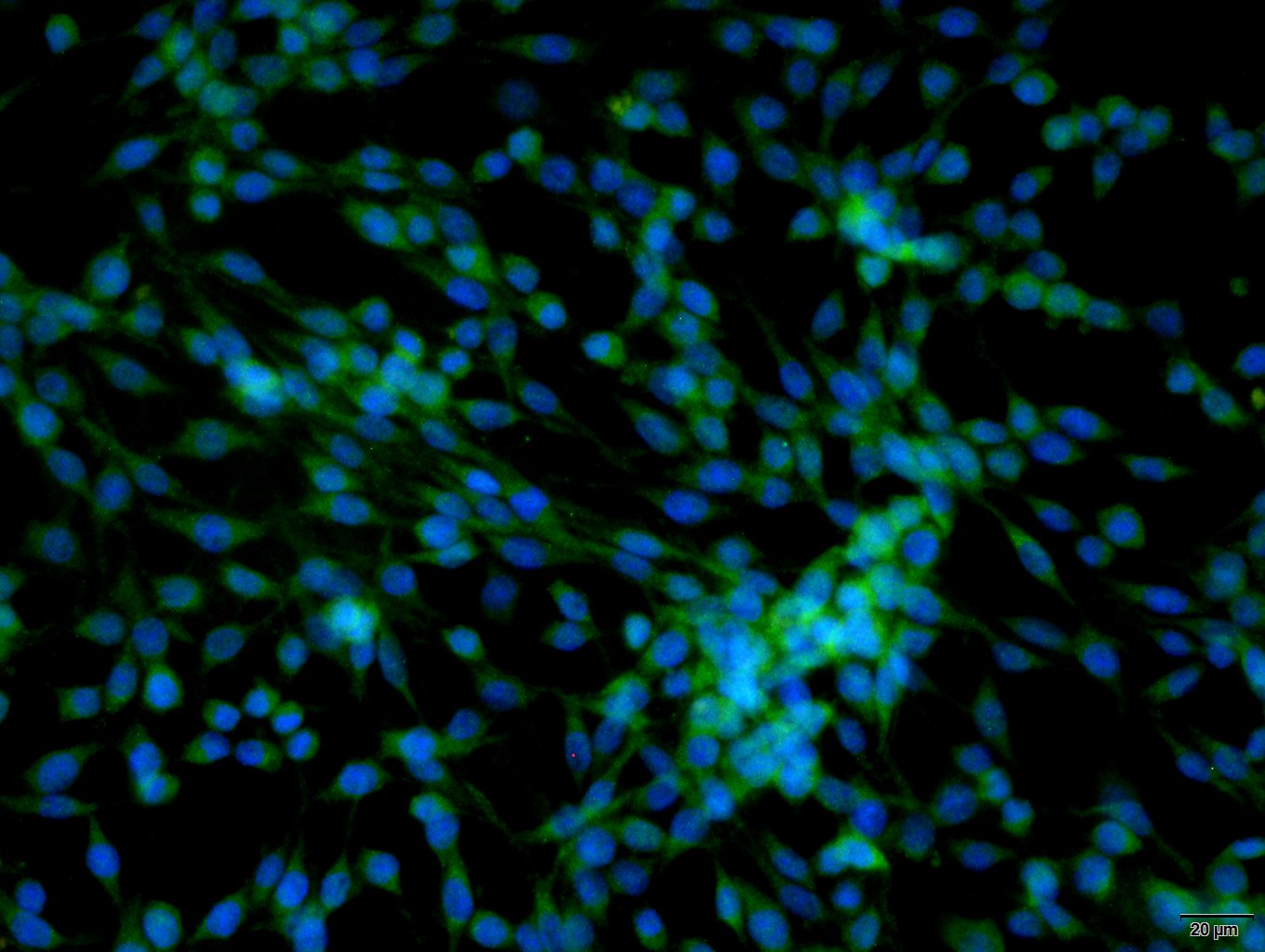

Supplement: Figure S7 — 21 photos comprising Figure 7. [file peerj-08-8254-s007.zip › Fig 7i.jpg]

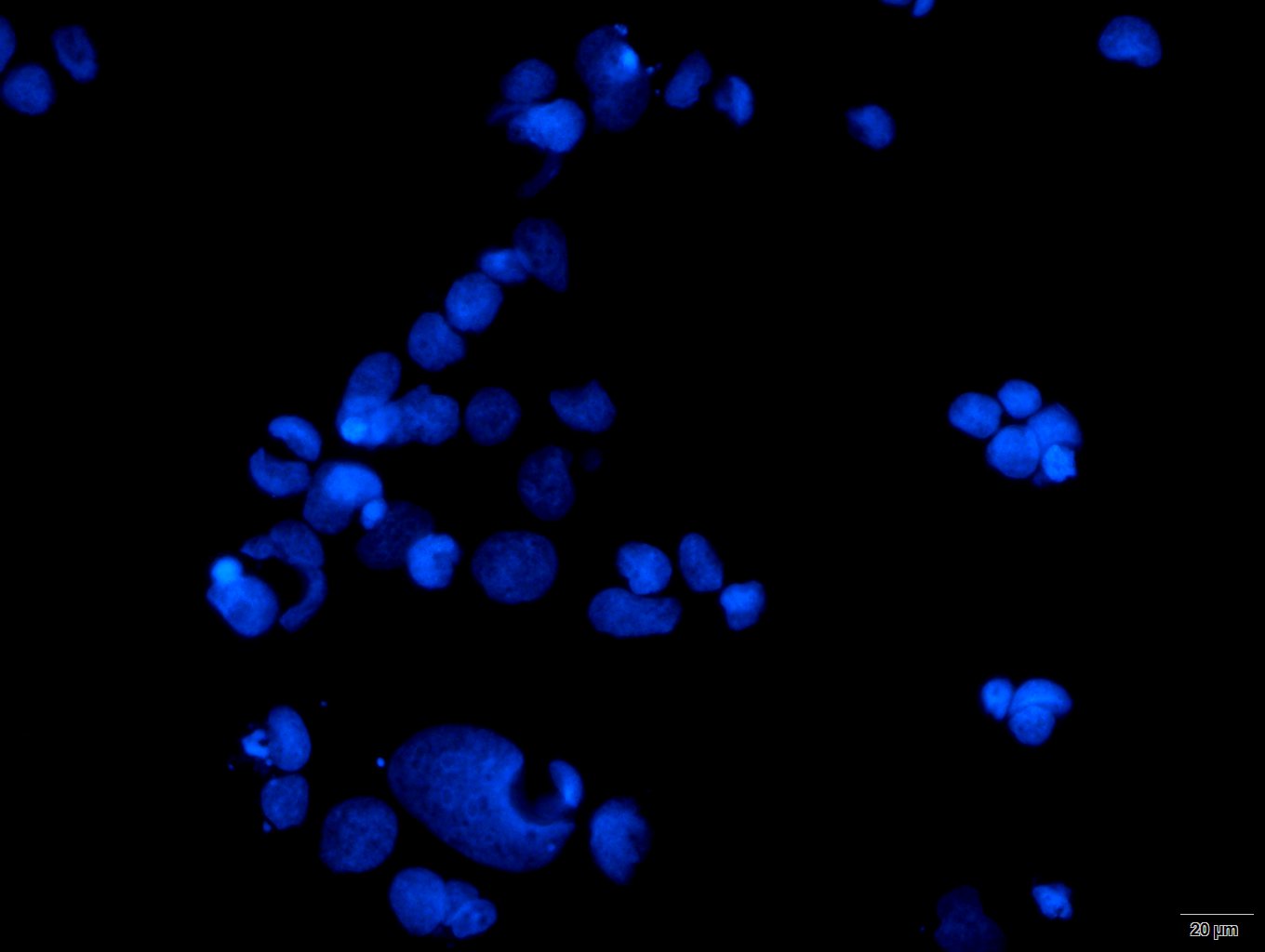

Supplement: Figure S7 — 21 photos comprising Figure 7. [file peerj-08-8254-s007.zip › Fig 7k.jpg]

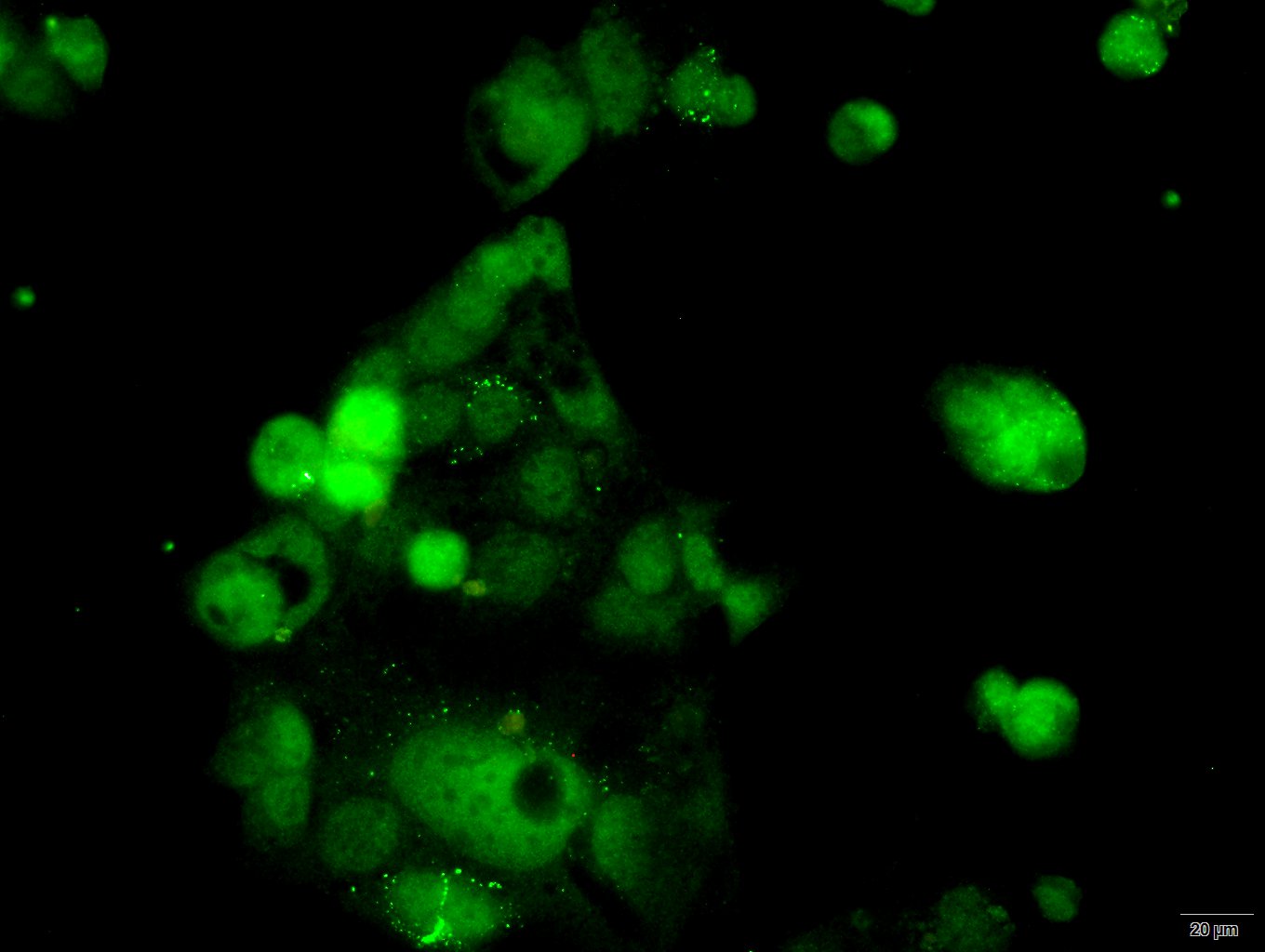

Supplement: Figure S7 — 21 photos comprising Figure 7. [file peerj-08-8254-s007.zip › Fig 7j.jpg]

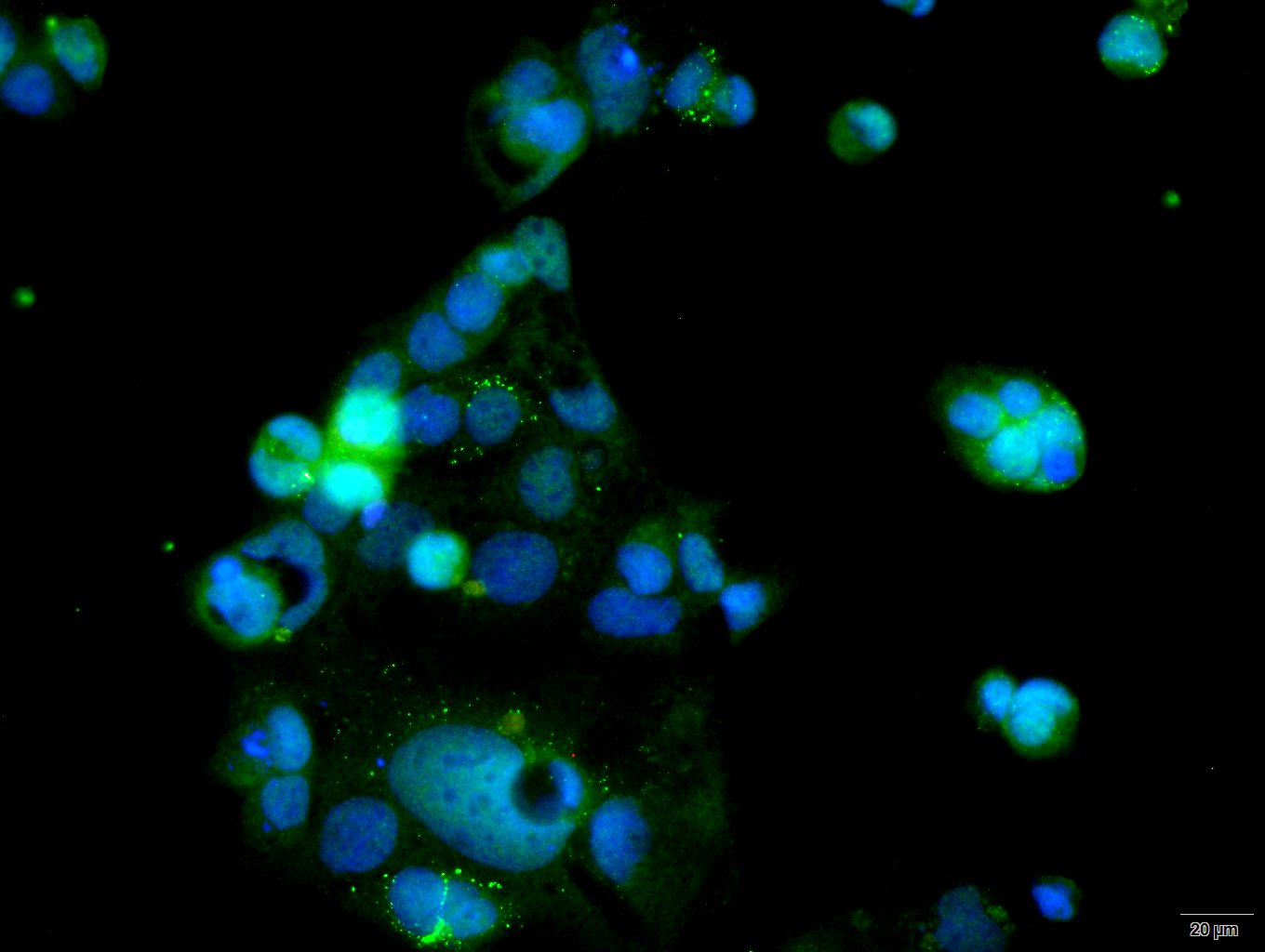

Supplement: Figure S7 — 21 photos comprising Figure 7. [file peerj-08-8254-s007.zip › Fig 7l.jpg]

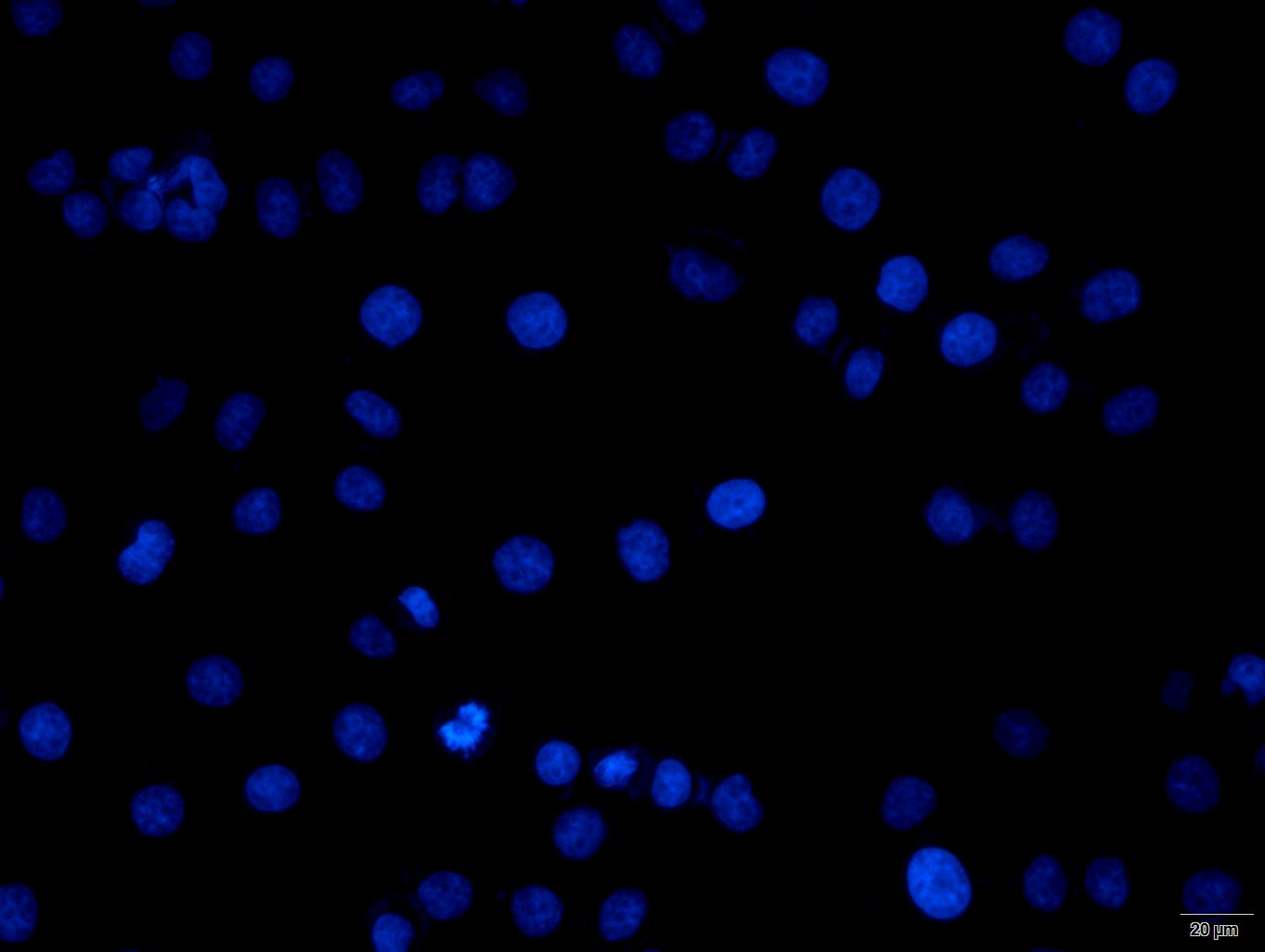

Supplement: Figure S7 — 21 photos comprising Figure 7. [file peerj-08-8254-s007.zip › Fig 7n.jpg]

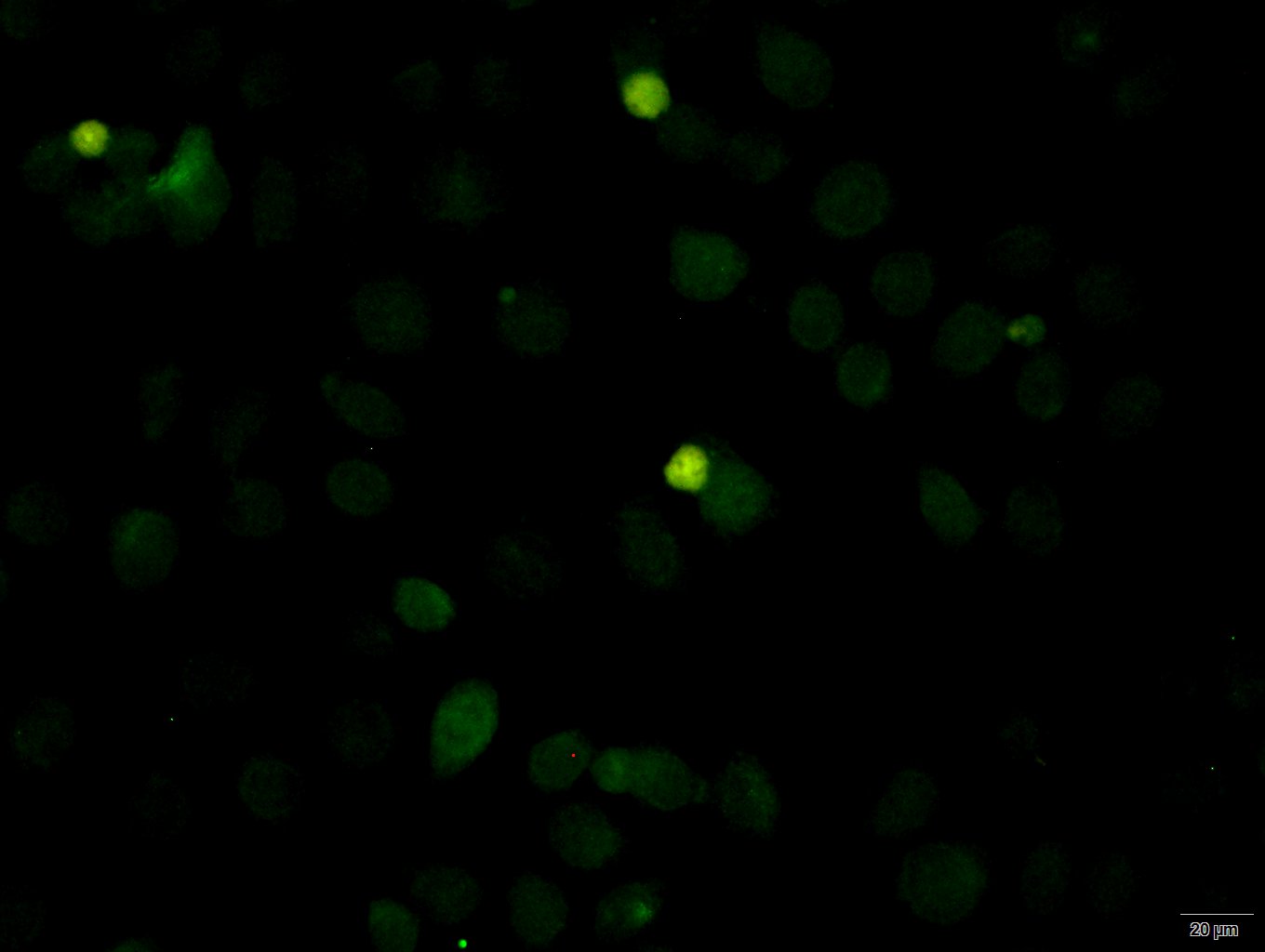

Supplement: Figure S7 — 21 photos comprising Figure 7. [file peerj-08-8254-s007.zip › Fig 7m.jpg]

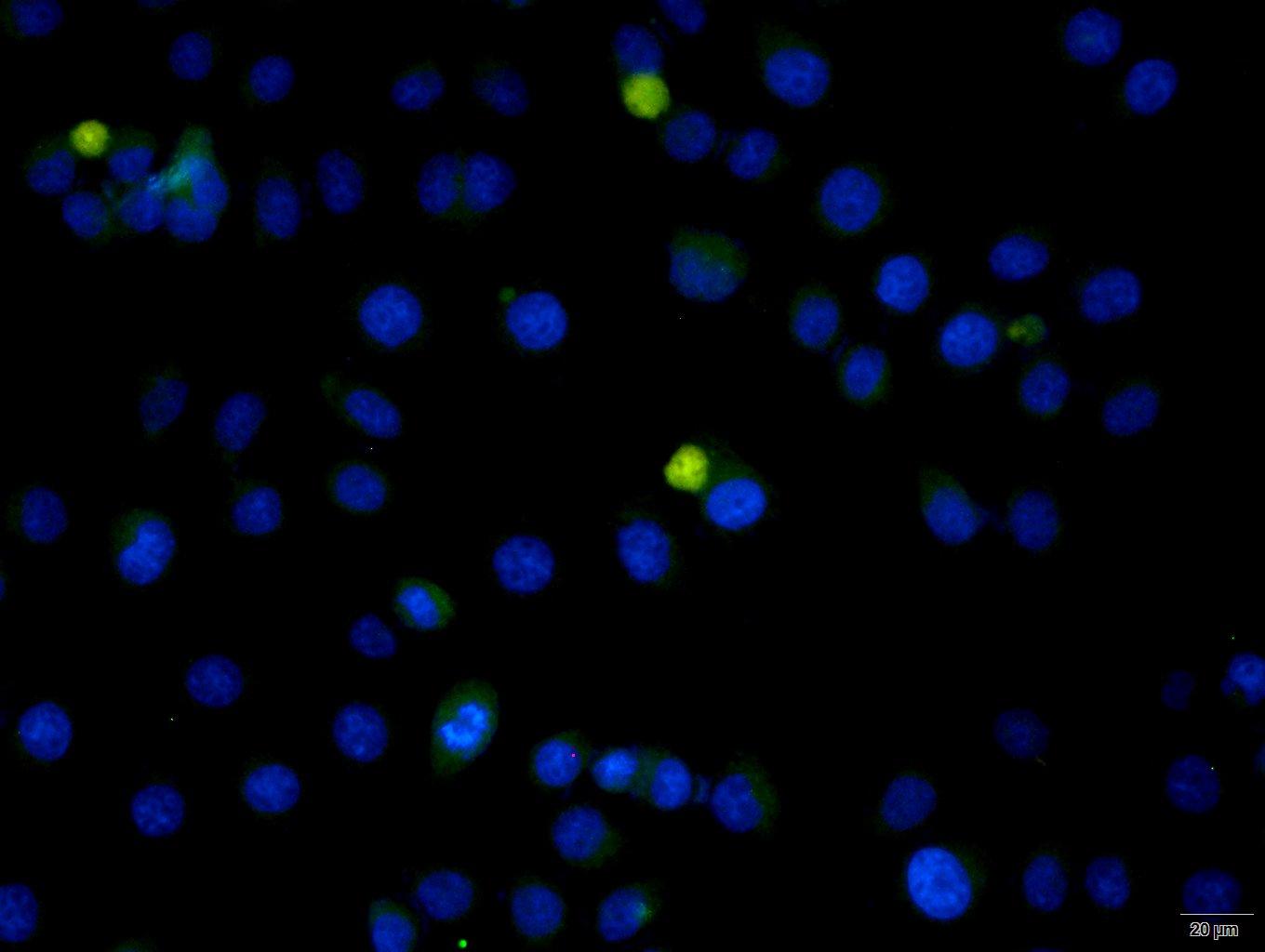

Supplement: Figure S7 — 21 photos comprising Figure 7. [file peerj-08-8254-s007.zip › Fig 7o.jpg]

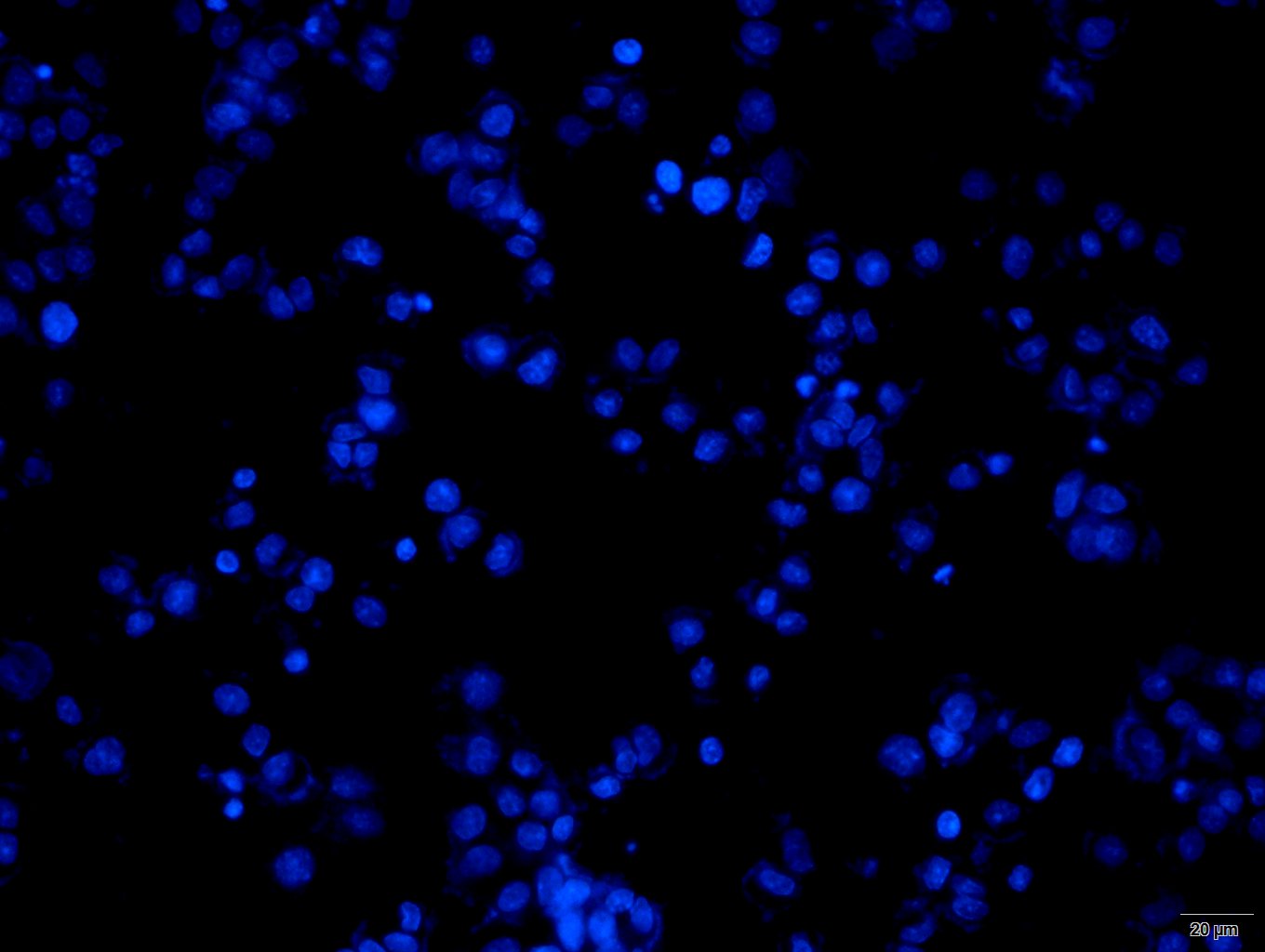

Supplement: Figure S7 — 21 photos comprising Figure 7. [file peerj-08-8254-s007.zip › Fig 7q.jpg]

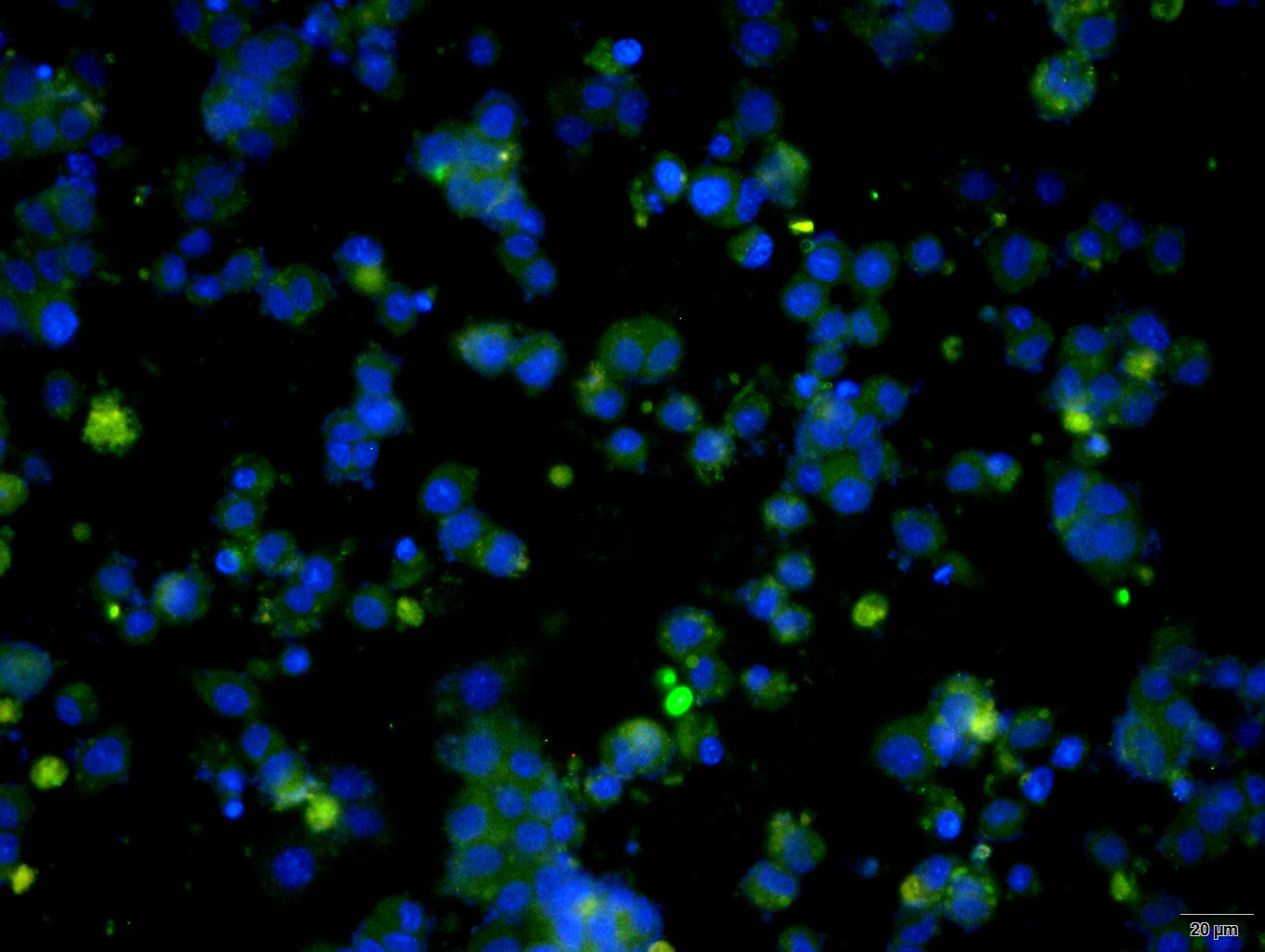

Supplement: Figure S7 — 21 photos comprising Figure 7. [file peerj-08-8254-s007.zip › Fig 7r.jpg]

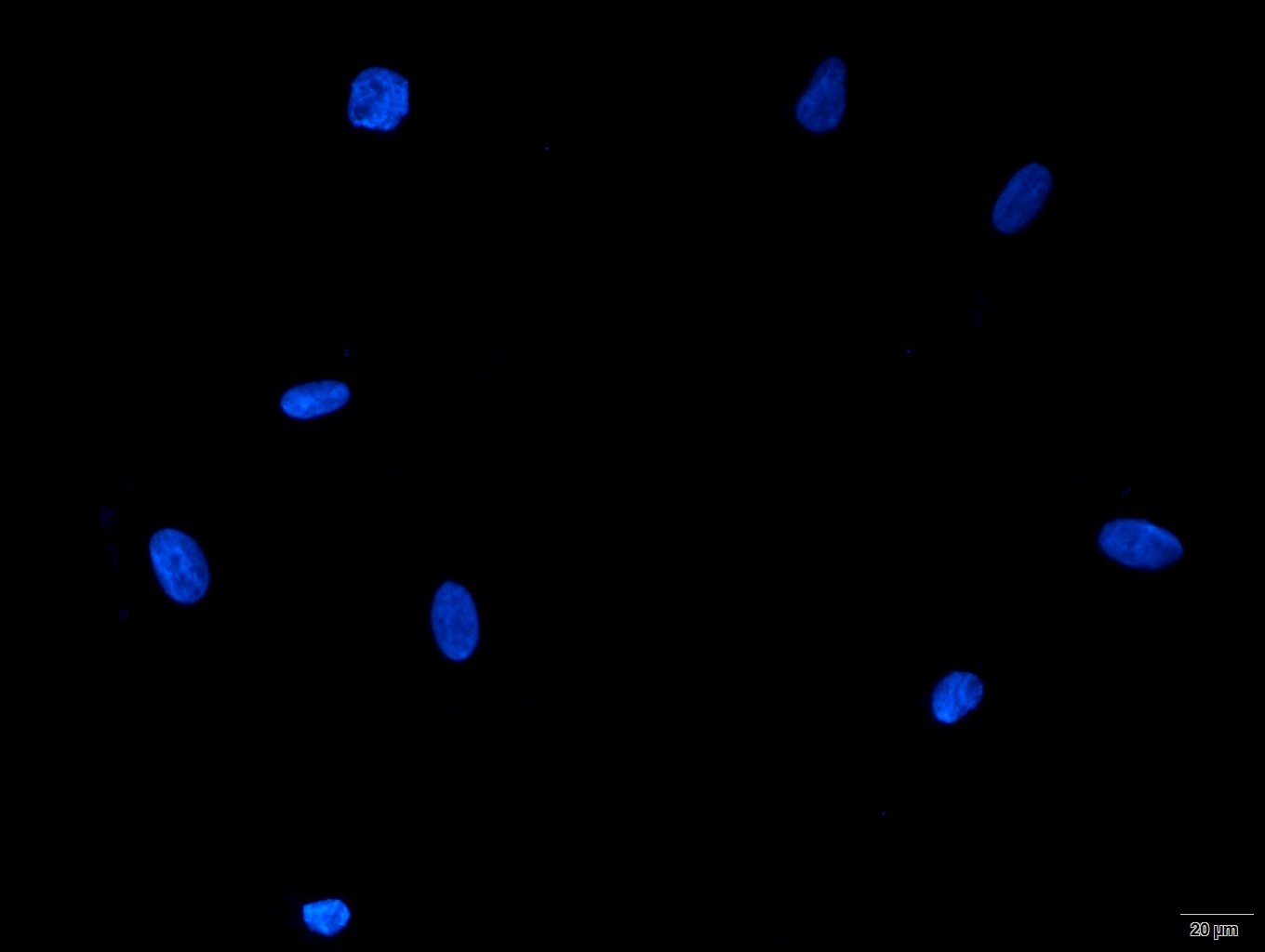

Supplement: Figure S7 — 21 photos comprising Figure 7. [file peerj-08-8254-s007.zip › Fig 7t.jpg]

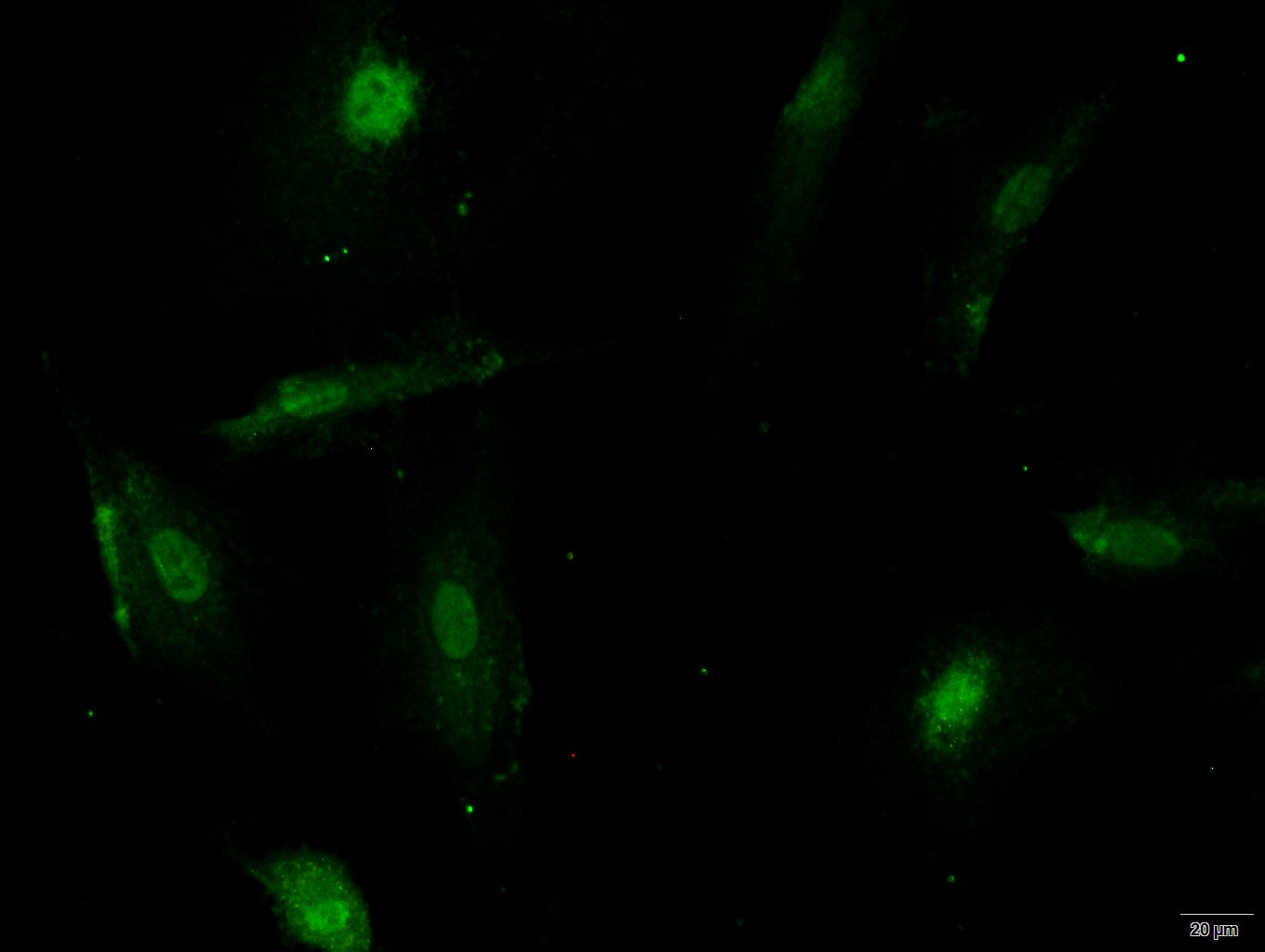

Supplement: Figure S7 — 21 photos comprising Figure 7. [file peerj-08-8254-s007.zip › Fig 7s.jpg]

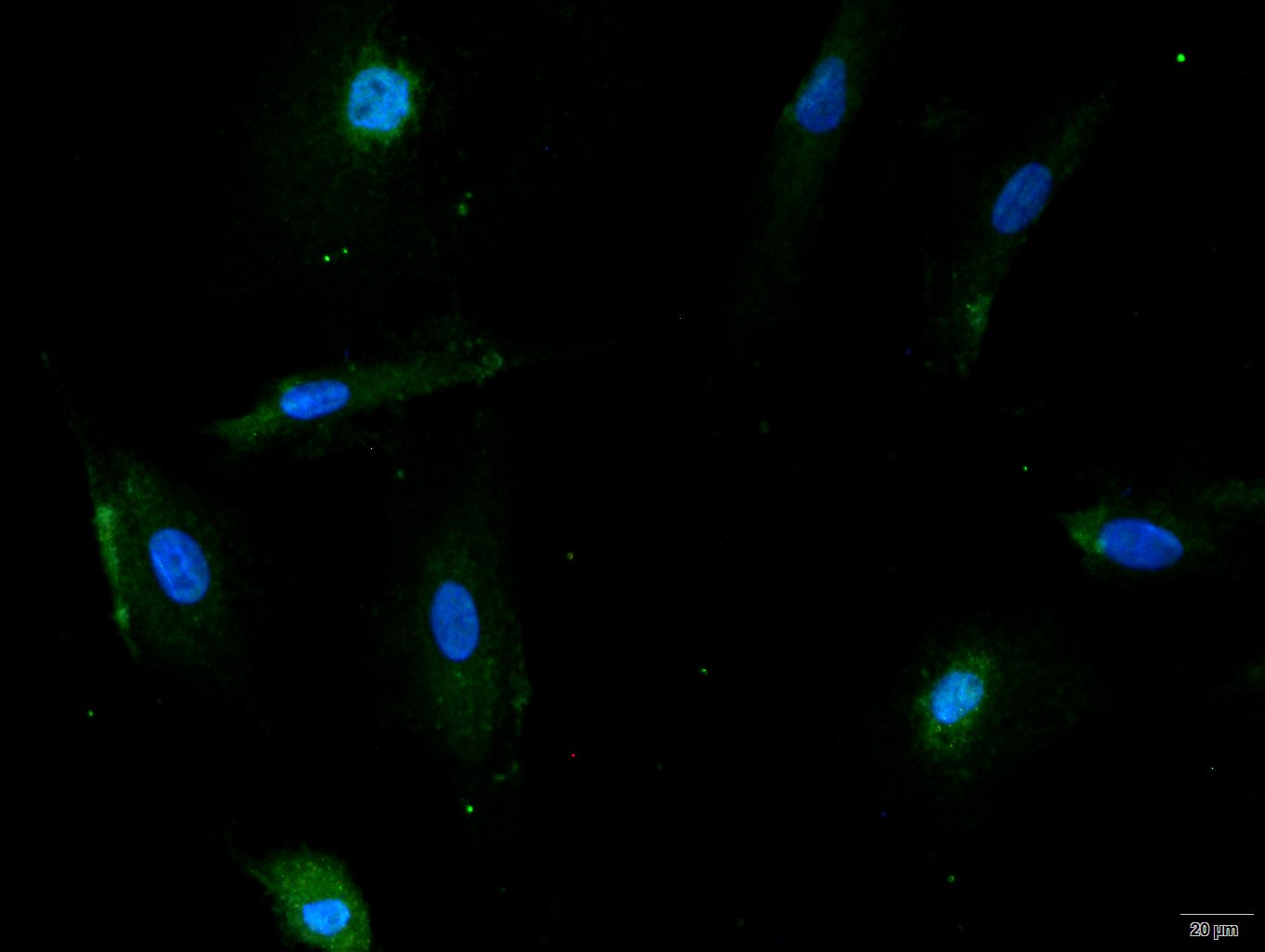

Supplement: Figure S7 — 21 photos comprising Figure 7. [file peerj-08-8254-s007.zip › Fig 7u.jpg]
