# Supplementary material for: A pilot study on searching for peri-nuclear NeuN-positive cells
Source: PeerJ. 2020 Jan 7;8:e8254. doi: 10.7717/peerj.8254 (PMC6953339; doi:10.7717/peerj.8254)
Supplement: Supplemental Information 8 — The genes’ original FPKM in eight organs, means of every gene in organs, SD of every gene in organs, P-values vs brain from analysis results and the documentation of the full steps of analysis. [file peerj-08-8254-s008.zip › Table 2 analysis.doc]

Analysis of Table 2 (List)

Heart vs Brain	1
Liver vs Brain	8
Lung vs Brain	15
Kidney vs Brain	22
Stomach vs Brain	27
Duodenum vs Brain	32
Ileum vs Brain	37


Heart vs Brain

NPAR TESTS
  /M-W= Eno2 Rbfox3 Map2 Tubb3 Dcx Chat Th Ncam1 Ncam2 Neurod1 Mapt Calb1 Calb2 Nefh Nefl Nefm Gfap S100b Vim Cnp BY group(1 2)
  /MISSING ANALYSIS.

NPar 检验 (NPar Test)

ªþª`(Note)	
创«Øªº输¥X(Output)	14-¤T¤ë-2019 08时07¤À21¬í	
ª`释(Note)	(14 Mar., 2019  08:07:21)	
输¤J(Input)	数Õu(Data)	F:\¤¤医学°|\NSFC\Uricase\转录组测§Ç\´²¸¨ªº¯«经¤¸\论¤å\Cell Bio Int\Raw data for uploading\analysis.sav	
	¬¡动ªº数Õu¶°(Active data set)	数Õu¶°1(Data set 1)	
	过滤¾¹(Filter)	<none>	
	权­«(Weight)	<none>	
	©î¤À¤å¥ó(Split files)	<none>	
	¤u§@数Õu¤å¥ó¤¤ªº N ¦æ(Lines)	24	
¯Ê¥¢­È处²z
(Missing value)	¯Ê¥¢©w义(Definition)	¥Î户©w义ªº¯Ê¥¢­È¥H¯Ê¥¢对«Ý¡C(According to the default)	
	¨Ï¥Îªº®×¨Ò (Cases)	¨C个检验ªº统计¶q³£¬O°ò¤_对¤_该检验©Ò¨Ï¥Îªº变¶q³£¨ã¦³¨ä¦³®Ä数Õuªº©Ò¦³®×¨Ò¡C(Based on all the cases)	
语ªk(Syntax)	NPAR TESTS
  /M-W= Eno2 Rbfox3 Map2 Tubb3 Dcx Chat Th Ncam1 Ncam2 Neurod1 Mapt Calb1 Calb2 Nefh Nefl Nefm Gfap S100b Vim Cnp BY group(1 2)
  /MISSING ANALYSIS.
	
资·½(Resource)	处²z¾¹时间 (Processor time)	00:00:00.016	
	¤w¥Î时间 (Elapsed time)	00:00:00.010	
	¤¹许ªº®×¨Ò数a
(Cases permissible)	30247	
a. °ò¤_¤u§@ªÅ间内¦sªº¥i¥Î©Ê¡C(Cases allowed for analysis based on the RAM)	


[数Õu¶°1] (Data set 1) F:\¤¤医学°|\NSFC\Uricase\转录组测§Ç\´²¸¨ªº¯«经¤¸\论¤å\Cell Bio Int\Raw data for uploading\analysis.sav


Mann-Whitney 检验 (Mann-Whitney Test)

¯´(Rank)	
	group	N	¯´§¡­È(Mean of rank)	¯´©M (Sum of rank)	
Eno2	dimension1	Brain	3	5.00	15.00	
		heart	3	2.00	6.00	
		总数
(Total)	6			
Rbfox3	dimension1	Brain	3	5.00	15.00	
		heart	3	2.00	6.00	
		总数
(Total)	6			
Map2	dimension1	Brain	3	5.00	15.00	
		heart	3	2.00	6.00	
		总数
(Total)	6			
Tubb3	dimension1	Brain	3	5.00	15.00	
		heart	3	2.00	6.00	
		总数
(Total)	6			
Dcx	dimension1	Brain	3	5.00	15.00	
		heart	3	2.00	6.00	
		总数
(Total)	6			
Chat	dimension1	Brain	3	5.00	15.00	
		heart	3	2.00	6.00	
		总数
(Total)	6			
Th	dimension1	Brain	3	5.00	15.00	
		heart	3	2.00	6.00	
		总数
(Total)	6			
Ncam1	dimension1	Brain	3	5.00	15.00	
		heart	3	2.00	6.00	
		总数
(Total)	6			
Ncam2	dimension1	Brain	3	5.00	15.00	
		heart	3	2.00	6.00	
		总数
(Total)	6			
Neurod1	dimension1	Brain	3	5.00	15.00	
		heart	3	2.00	6.00	
		总数	6			
Mapt	dimension1	Brain	3	5.00	15.00	
		heart	3	2.00	6.00	
		总数
(Total)	6			
Calb1	dimension1	Brain	3	5.00	15.00	
		heart	3	2.00	6.00	
		总数
(Total)	6			
Calb2	dimension1	Brain	3	5.00	15.00	
		heart	3	2.00	6.00	
		总数
(Total)	6			
Nefh	dimension1	Brain	3	5.00	15.00	
		heart	3	2.00	6.00	
		总数
(Total)	6			
Nefl	dimension1	Brain	3	5.00	15.00	
		heart	3	2.00	6.00	
		总数(Total)	6			
Nefm	dimension1	Brain	3	5.00	15.00	
		heart	3	2.00	6.00	
		总数
(Total)	6			
Gfap	dimension1	Brain	3	5.00	15.00	
		heart	3	2.00	6.00	
		总数
(Total)	6			
S100b	dimension1	Brain	3	5.00	15.00	
		heart	3	2.00	6.00	
		总数
(Total)	6			
Vim	dimension1	Brain	3	2.00	6.00	
		heart	3	5.00	15.00	
		总数
(Total)	6			
Cnp	dimension1	Brain	3	5.00	15.00	
		heart	3	2.00	6.00	
		总数
(Total)	6			


检验统计¶qb(Test statistics)	
	Eno2	Rbfox3	Map2	Tubb3	Dcx	Chat	
Mann-Whitney U	.000	.000	.000	.000	.000	.000	
Wilcoxon W	6.000	6.000	6.000	6.000	6.000	6.000	
Z	-1.964	-1.964	-1.964	-1.964	-1.993	-1.993	
渐ªñ显µÛ©Ê(双侧) Asymptotic significance (bilateral)	.050	.050	.050	.050	.046	.046	
ºëÚÌ显µÛ©Ê[2*¡]单侧显µÛ©Ê¡^]
Precise significance [2* (unilateral significance)]	.100a	.100a	.100a	.100a	.100a	.100a	
a. 没¦³对结进¦æ­×¥¿¡C(No modifications were made)	
b. ¤À组变¶q(Grouping variable): group	

检验统计¶qb(Test statistics)	
	Th	Ncam1	Ncam2	Neurod1	Mapt	Calb1	
Mann-Whitney U	.000	.000	.000	.000	.000	.000	
Wilcoxon W	6.000	6.000	6.000	6.000	6.000	6.000	
Z	-1.993	-1.964	-1.964	-1.993	-1.964	-1.993	
渐ªñ显µÛ©Ê(双侧) Asymptotic significance (bilateral)	.046	.050	.050	.046	.050	.046	
ºëÚÌ显µÛ©Ê[2*¡]单侧显µÛ©Ê¡^] Precise significance [2* (unilateral significance)]	.100a	.100a	.100a	.100a	.100a	.100a	
a. 没¦³对结进¦æ­×¥¿¡C(No modifications were made)	
b. ¤À组变¶q(Grouping variable): group
	

检验统计¶qb(Test statistics)	
	Calb2	Nefh	Nefl	Nefm	Gfap	S100b	
Mann-Whitney U	.000	.000	.000	.000	.000	.000	
Wilcoxon W	6.000	6.000	6.000	6.000	6.000	6.000	
Z	-1.964	-1.964	-1.993	-1.964	-1.964	-1.964	
渐ªñ显µÛ©Ê(双侧) Asymptotic significance (bilateral)	.050	.050	.046	.050	.050	.050	
ºëÚÌ显µÛ©Ê[2*¡]单侧显µÛ©Ê¡^] Precise significance [2* (unilateral significance)]	.100a	.100a	.100a	.100a	.100a	.100a	
a. 没¦³对结进¦æ­×¥¿¡C(No modifications were made)	
b. ¤À组变¶q(Grouping variable): group	

检验统计¶qb(Test statistics)	
	Vim	Cnp	
Mann-Whitney U	.000	.000	
Wilcoxon W	6.000	6.000	
Z	-1.964	-1.964	
渐ªñ显µÛ©Ê(双侧) Asymptotic significance (bilateral)	.050	.050	
ºëÚÌ显µÛ©Ê[2*¡]单侧显µÛ©Ê¡^]	.100a	.100a	
a. 没¦³对结进¦æ­×¥¿¡C(No modifications were made)	
b. ¤À组变¶q(Grouping variable): group	


Liver vs Brain

NPAR TESTS
  /M-W= Eno2 Rbfox3 Map2 Tubb3 Dcx Chat Th Ncam1 Ncam2 Neurod1 Mapt Calb1 Calb2 Nefh Nefl Nefm Gfap S100b Vim Cnp BY group(1 3)
  /MISSING ANALYSIS.

NPar 检验 (NPar Test)
ªþª` (Note)	
创«Øªº输¥X(Output)	14-¤T¤ë-2019 08时08¤À21¬í	
ª`释(Note)	 (14 Mar., 2019  08:08:21)	
输¤J(Input)	数Õu(Data)	F:\¤¤医学°|\NSFC\Uricase\转录组测§Ç\´²¸¨ªº¯«经¤¸\论¤å\Cell Bio Int\Raw data for uploading\analysis.sav	
	¬¡动ªº数Õu¶°(Active data set)	数Õu¶°1(Data set 1)	
	过滤¾¹(Filter)	<none>	
	权­«(Weight)	<none>	
	©î¤À¤å¥ó(Split files)	<none>	
	¤u§@数Õu¤å¥ó¤¤ªº N ¦æ(Lines)	24	
¯Ê¥¢­È处²z
(Missing value)	¯Ê¥¢©w义(Definition)	¥Î户©w义ªº¯Ê¥¢­È¥H¯Ê¥¢对«Ý¡C(According to the default)	
	¨Ï¥Îªº®×¨Ò(Cases)	¨C个检验ªº统计¶q³£¬O°ò¤_对¤_该检验©Ò¨Ï¥Îªº变¶q³£¨ã¦³¨ä¦³®Ä数Õuªº©Ò¦³®×¨Ò¡C(Based on all the cases)	
语ªk(Syntax)	NPAR TESTS
  /M-W= Eno2 Rbfox3 Map2 Tubb3 Dcx Chat Th Ncam1 Ncam2 Neurod1 Mapt Calb1 Calb2 Nefh Nefl Nefm Gfap S100b Vim Cnp BY group(1 3)
  /MISSING ANALYSIS.
	
资·½(Resource)	处²z¾¹时间(Processor time)	00:00:00.000	
	¤w¥Î时间(Elapsed time)	00:00:00.010	
	¤¹许ªº®×¨Ò数a
(Cases permissible)	30247	
a. °ò¤_¤u§@ªÅ间内¦sªº¥i¥Î©Ê¡C(Cases allowed for analysis based on the RAM)	


[数Õu¶°1] (Data set 1) F:\¤¤医学°|\NSFC\Uricase\转录组测§Ç\´²¸¨ªº¯«经¤¸\论¤å\Cell Bio Int\Raw data for uploading\analysis.sav


Mann-Whitney 检验(Mann-Whitney Test)

¯´(Rank)	
	group	N	¯´§¡­È(Mean of rank)	¯´©M (Sum of rank)	
Eno2	dimension1	Brain	3	5.00	15.00	
		liver	3	2.00	6.00	
		总数	6			
Rbfox3	dimension1	Brain	3	5.00	15.00	
		liver	3	2.00	6.00	
		总数	6			
Map2	dimension1	Brain	3	5.00	15.00	
		liver	3	2.00	6.00	
		总数	6			
Tubb3	dimension1	Brain	3	5.00	15.00	
		liver	3	2.00	6.00	
		总数	6			
Dcx	dimension1	Brain	3	5.00	15.00	
		liver	3	2.00	6.00	
		总数	6			
Chat	dimension1	Brain	3	5.00	15.00	
		liver	3	2.00	6.00	
		总数	6			
Th	dimension1	Brain	3	5.00	15.00	
		liver	3	2.00	6.00	
		总数	6			
Ncam1	dimension1	Brain	3	5.00	15.00	
		liver	3	2.00	6.00	
		总数	6			
Ncam2	dimension1	Brain	3	5.00	15.00	
		liver	3	2.00	6.00	
		总数	6			
Neurod1	dimension1	Brain	3	5.00	15.00	
		liver	3	2.00	6.00	
		总数	6			
Mapt	dimension1	Brain	3	5.00	15.00	
		liver	3	2.00	6.00	
		总数	6			
Calb1	dimension1	Brain	3	5.00	15.00	
		liver	3	2.00	6.00	
		总数	6			
Calb2	dimension1	Brain	3	5.00	15.00	
		liver	3	2.00	6.00	
		总数	6			
Nefh	dimension1	Brain	3	5.00	15.00	
		liver	3	2.00	6.00	
		总数
(Total)	6			
Nefl	dimension1	Brain	3	5.00	15.00	
		liver	3	2.00	6.00	
		总数
(Total)	6			
Nefm	dimension1	Brain	3	5.00	15.00	
		liver	3	2.00	6.00	
		总数
(Total)	6			
Gfap	dimension1	Brain	3	5.00	15.00	
		liver	3	2.00	6.00	
		总数
(Total)	6			
S100b	dimension1	Brain	3	5.00	15.00	
		liver	3	2.00	6.00	
		总数
(Total)	6			
Vim	dimension1	Brain	3	5.00	15.00	
		liver	3	2.00	6.00	
		总数
(Total)	6			
Cnp	dimension1	Brain	3	5.00	15.00	
		liver	3	2.00	6.00	
		总数
(Total)	6			


检验统计¶qb(Test statistics)	
	Eno2	Rbfox3	Map2	Tubb3	Dcx	Chat	
Mann-Whitney U	.000	.000	.000	.000	.000	.000	
Wilcoxon W	6.000	6.000	6.000	6.000	6.000	6.000	
Z	-1.964	-1.993	-1.964	-1.964	-1.993	-2.087	
渐ªñ显µÛ©Ê(双侧) Asymptotic significance (bilateral)	.050	.046	.050	.050	.046	.037	
ºëÚÌ显µÛ©Ê[2*¡]单侧显µÛ©Ê¡^]
Precise significance [2* (unilateral significance)]	.100a	.100a	.100a	.100a	.100a	.100a	
a. 没¦³对结进¦æ­×¥¿¡C(No modifications were made)	
b. ¤À组变¶q(Grouping variable): group	

检验统计¶qb(Test statistics)	
	Th	Ncam1	Ncam2	Neurod1	Mapt	Calb1	
Mann-Whitney U	.000	.000	.000	.000	.000	.000	
Wilcoxon W	6.000	6.000	6.000	6.000	6.000	6.000	
Z	-2.087	-1.964	-1.964	-1.993	-1.964	-2.087	
渐ªñ显µÛ©Ê(双侧) Asymptotic significance (bilateral)	.037	.050	.050	.046	.050	.037	
ºëÚÌ显µÛ©Ê[2*¡]单侧显µÛ©Ê¡^]
Precise significance [2* (unilateral significance)]	.100a	.100a	.100a	.100a	.100a	.100a	
a. 没¦³对结进¦æ­×¥¿¡C(No modifications were made)	
b. ¤À组变¶q(Grouping variable): group	

检验统计¶qb(Test statistics)	
	Calb2	Nefh	Nefl	Nefm	Gfap	S100b	
Mann-Whitney U	.000	.000	.000	.000	.000	.000	
Wilcoxon W	6.000	6.000	6.000	6.000	6.000	6.000	
Z	-2.087	-1.993	-1.993	-1.964	-1.964	-1.964	
渐ªñ显µÛ©Ê(双侧) Asymptotic significance (bilateral)	.037	.046	.046	.050	.050	.050	
ºëÚÌ显µÛ©Ê[2*¡]单侧显µÛ©Ê¡^]
Precise significance [2* (unilateral significance)]	.100a	.100a	.100a	.100a	.100a	.100a	
a. 没¦³对结进¦æ­×¥¿¡C(No modifications were made)	
b. ¤À组变¶q(Grouping variable): group	

检验统计¶qb(Test statistics)	
	Vim	Cnp	
Mann-Whitney U	.000	.000	
Wilcoxon W	6.000	6.000	
Z	-1.993	-1.964	
渐ªñ显µÛ©Ê(双侧) Asymptotic significance (bilateral)	.046	.050	
ºëÚÌ显µÛ©Ê[2*¡]单侧显µÛ©Ê¡^]
Precise significance [2* (unilateral significance)]	.100a	.100a	
a. 没¦³对结进¦æ­×¥¿¡C(No modifications were made)	
b. ¤À组变¶q(Grouping variable): group	


Lung vs Brain

NPAR TESTS
  /M-W= Eno2 Rbfox3 Map2 Tubb3 Dcx Chat Th Ncam1 Ncam2 Neurod1 Mapt Calb1 Calb2 Nefh Nefl Nefm Gfap S100b Vim Cnp BY group(1 4)
  /MISSING ANALYSIS.

NPar 检验 (NPar Test)

ªþª`(Note)	
创«Øªº输¥X(Output)	14-¤T¤ë-2019 08时08¤À53¬í	
ª`释(Note)	 (14 Mar., 2019  08:08:53)	
输¤J(Input)	数Õu(Data)	F:\¤¤医学°|\NSFC\Uricase\转录组测§Ç\´²¸¨ªº¯«经¤¸\论¤å\Cell Bio Int\Raw data for uploading\analysis.sav	
	¬¡动ªº数Õu¶°(Active data set)	数Õu¶°1(Data set 1)	
	过滤¾¹(Filter)	<none>	
	权­«(Weight)	<none>	
	©î¤À¤å¥ó(Split files)	<none>	
	¤u§@数Õu¤å¥ó¤¤ªº N ¦æ(Lines)	24	
¯Ê¥¢­È处²z
(Missing value)	¯Ê¥¢©w义(Definition)	¥Î户©w义ªº¯Ê¥¢­È¥H¯Ê¥¢对«Ý¡C(According to the default)	
	¨Ï¥Îªº®×¨Ò (Cases)	¨C个检验ªº统计¶q³£¬O°ò¤_对¤_该检验©Ò¨Ï¥Îªº变¶q³£¨ã¦³¨ä¦³®Ä数Õuªº©Ò¦³®×¨Ò¡C(Based on all the cases)	
语ªk(Syntax)	NPAR TESTS
  /M-W= Eno2 Rbfox3 Map2 Tubb3 Dcx Chat Th Ncam1 Ncam2 Neurod1 Mapt Calb1 Calb2 Nefh Nefl Nefm Gfap S100b Vim Cnp BY group(1 4)
  /MISSING ANALYSIS.
	
资·½(Resource)	处²z¾¹时间 (Processor time)	00:00:00.000	
	¤w¥Î时间 (Elapsed time)	00:00:00.000	
	¤¹许ªº®×¨Ò数a
(Cases permissible)	30247	
a. °ò¤_¤u§@ªÅ间内¦sªº¥i¥Î©Ê¡C(Cases allowed for analysis based on the RAM)	


[数Õu¶°1] (Data set 1)  F:\¤¤医学°|\NSFC\Uricase\转录组测§Ç\´²¸¨ªº¯«经¤¸\论¤å\Cell Bio Int\Raw data for uploading\analysis.sav


Mann-Whitney 检验 (Mann-Whitney Test)


¯´(Rank)	
	group	N	¯´§¡­È(Mean of rank)	¯´©M (Sum of rank)	
Eno2	dimension1	Brain	3	5.00	15.00	
		lung	3	2.00	6.00	
		总数
(Total)	6			
Rbfox3	dimension1	Brain	3	5.00	15.00	
		lung	3	2.00	6.00	
		总数
(Total)	6			
Map2	dimension1	Brain	3	5.00	15.00	
		lung	3	2.00	6.00	
		总数
(Total)	6			
Tubb3	dimension1	Brain	3	5.00	15.00	
		lung	3	2.00	6.00	
		总数
(Total)	6			
Dcx	dimension1	Brain	3	5.00	15.00	
		lung	3	2.00	6.00	
		总数
(Total)	6			
Chat	dimension1	Brain	3	5.00	15.00	
		lung	3	2.00	6.00	
		总数
(Total)	6			
Th	dimension1	Brain	3	5.00	15.00	
		lung	3	2.00	6.00	
		总数
(Total)	6			
Ncam1	dimension1	Brain	3	5.00	15.00	
		lung	3	2.00	6.00	
		总数
(Total)	6			
Ncam2	dimension1	Brain	3	5.00	15.00	
		lung	3	2.00	6.00	
		总数
(Total)	6			
Neurod1	dimension1	Brain	3	5.00	15.00	
		lung	3	2.00	6.00	
		总数
(Total)	6			
Mapt	dimension1	Brain	3	5.00	15.00	
		lung	3	2.00	6.00	
		总数
(Total)	6			
Calb1	dimension1	Brain	3	5.00	15.00	
		lung	3	2.00	6.00	
		总数
(Total)	6			
Calb2	dimension1	Brain	3	5.00	15.00	
		lung	3	2.00	6.00	
		总数
(Total)	6			
Nefh	dimension1	Brain	3	5.00	15.00	
		lung	3	2.00	6.00	
		总数
(Total)	6			
Nefl	dimension1	Brain	3	5.00	15.00	
		lung	3	2.00	6.00	
		总数
(Total)	6			
Nefm	dimension1	Brain	3	5.00	15.00	
		lung	3	2.00	6.00	
		总数
(Total)	6			
Gfap	dimension1	Brain	3	5.00	15.00	
		lung	3	2.00	6.00	
		总数
(Total)	6			
S100b	dimension1	Brain	3	5.00	15.00	
		lung	3	2.00	6.00	
		总数
(Total)	6			
Vim	dimension1	Brain	3	2.00	6.00	
		lung	3	5.00	15.00	
		总数
(Total)	6			
Cnp	dimension1	Brain	3	5.00	15.00	
		lung	3	2.00	6.00	
		总数
(Total)	6			


检验统计¶qb(Test statistics)	
	Eno2	Rbfox3	Map2	Tubb3	Dcx	Chat	
Mann-Whitney U	.000	.000	.000	.000	.000	.000	
Wilcoxon W	6.000	6.000	6.000	6.000	6.000	6.000	
Z	-1.964	-1.964	-1.964	-1.964	-1.964	-1.964	
渐ªñ显µÛ©Ê(双侧) Asymptotic significance (bilateral)	.050	.050	.050	.050	.050	.050	
ºëÚÌ显µÛ©Ê[2*¡]单侧显µÛ©Ê¡^]
Precise significance [2* (unilateral significance)]	.100a	.100a	.100a	.100a	.100a	.100a	
a. 没¦³对结进¦æ­×¥¿¡C(No modifications were made)	
b. ¤À组变¶q(Grouping variable): group	

检验统计¶qb(Test statistics)	
	Th	Ncam1	Ncam2	Neurod1	Mapt	Calb1	
Mann-Whitney U	.000	.000	.000	.000	.000	.000	
Wilcoxon W	6.000	6.000	6.000	6.000	6.000	6.000	
Z	-1.964	-1.964	-1.993	-1.993	-1.964	-1.964	
渐ªñ显µÛ©Ê(双侧) Asymptotic significance (bilateral)	.050	.050	.046	.046	.050	.050	
ºëÚÌ显µÛ©Ê[2*¡]单侧显µÛ©Ê¡^]
Precise significance [2* (unilateral significance)]	.100a	.100a	.100a	.100a	.100a	.100a	
a. 没¦³对结进¦æ­×¥¿¡C(No modifications were made)	
b. ¤À组变¶q(Grouping variable): group	

检验统计¶qb(Test statistics)	
	Calb2	Nefh	Nefl	Nefm	Gfap	S100b	
Mann-Whitney U	.000	.000	.000	.000	.000	.000	
Wilcoxon W	6.000	6.000	6.000	6.000	6.000	6.000	
Z	-1.964	-1.964	-1.964	-1.964	-1.964	-1.964	
渐ªñ显µÛ©Ê(双侧) Asymptotic significance (bilateral)	.050	.050	.050	.050	.050	.050	
ºëÚÌ显µÛ©Ê[2*¡]单侧显µÛ©Ê¡^]
Precise significance [2* (unilateral significance)]	.100a	.100a	.100a	.100a	.100a	.100a	
a. 没¦³对结进¦æ­×¥¿¡C(No modifications were made)	
b. ¤À组变¶q(Grouping variable): group	

检验统计¶qb(Test statistics)	
	Vim	Cnp	
Mann-Whitney U	.000	.000	
Wilcoxon W	6.000	6.000	
Z	-1.964	-1.964	
渐ªñ显µÛ©Ê(双侧) Asymptotic significance (bilateral)	.050	.050	
ºëÚÌ显µÛ©Ê[2*¡]单侧显µÛ©Ê¡^]
Precise significance [2* (unilateral significance)]	.100a	.100a	
a. 没¦³对结进¦æ­×¥¿¡C(No modifications were made)	
b. ¤À组变¶q(Grouping variable): group	


Kidney vs Brain

NPAR TESTS
  /M-W= Eno2 Rbfox3 Map2 Tubb3 Dcx Chat Th Ncam1 Ncam2 Neurod1 Mapt Calb1 Calb2 Nefh Nefl Nefm Gfap S100b Vim Cnp BY group(1 5)
  /MISSING ANALYSIS.

NPar 检验 (NPar Test)

ªþª`(Note)	
创«Øªº输¥X(Output)	14-¤T¤ë-2019 08时09¤À23¬í	
ª`释(Note)	 (14 Mar., 2019  08:09:23)	
输¤J(Input)	数Õu(Data)	F:\¤¤医学°|\NSFC\Uricase\转录组测§Ç\´²¸¨ªº¯«经¤¸\论¤å\Cell Bio Int\Raw data for uploading\analysis.sav	
	¬¡动ªº数Õu¶°(Active data set)	数Õu¶°1(Data set 1)	
	过滤¾¹(Filter)	<none>	
	权­«(Weight)	<none>	
	©î¤À¤å¥ó(Split files)	<none>	
	¤u§@数Õu¤å¥ó¤¤ªº N ¦æ(Lines)	24	
¯Ê¥¢­È处²z
(Missing value)	¯Ê¥¢©w义(Definition)	¥Î户©w义ªº¯Ê¥¢­È¥H¯Ê¥¢对«Ý¡C(According to the default)	
	¨Ï¥Îªº®×¨Ò (Cases)	¨C个检验ªº统计¶q³£¬O°ò¤_对¤_该检验©Ò¨Ï¥Îªº变¶q³£¨ã¦³¨ä¦³®Ä数Õuªº©Ò¦³®×¨Ò¡C(Based on all the cases)	
语ªk(Syntax)	NPAR TESTS
  /M-W= Eno2 Rbfox3 Map2 Tubb3 Dcx Chat Th Ncam1 Ncam2 Neurod1 Mapt Calb1 Calb2 Nefh Nefl Nefm Gfap S100b Vim Cnp BY group(1 5)
  /MISSING ANALYSIS.
	
资·½(Resource)	处²z¾¹时间 (Processor time)	00:00:00.000	
	¤w¥Î时间 (Elapsed time)	00:00:00.010	
	¤¹许ªº®×¨Ò数a
(Cases permissible)	30247	
a. °ò¤_¤u§@ªÅ间内¦sªº¥i¥Î©Ê¡C(Cases allowed for analysis based on the RAM)	


[数Õu¶°1] (Data set 1)  F:\¤¤医学°|\NSFC\Uricase\转录组测§Ç\´²¸¨ªº¯«经¤¸\论¤å\Cell Bio Int\Raw data for uploading\analysis.sav


Mann-Whitney 检验 (Mann-Whitney Test)


¯´(Rank)	
	group	N	¯´§¡­È(Mean of rank)	¯´©M (Sum of rank)	
Eno2	Brain	3	5.00	15.00	
	kidney	3	2.00	6.00	
	总数
(Total)	6			
Rbfox3	Brain	3	5.00	15.00	
	kidney	3	2.00	6.00	
	总数
(Total)	6			
Map2	Brain	3	5.00	15.00	
	kidney	3	2.00	6.00	
	总数
(Total)	6			
Tubb3	Brain	3	5.00	15.00	
	kidney	3	2.00	6.00	
	总数
(Total)	6			
Dcx	Brain	3	5.00	15.00	
	kidney	3	2.00	6.00	
	总数
(Total)	6			
Chat	Brain	3	5.00	15.00	
	kidney	3	2.00	6.00	
	总数
(Total)	6			
Th	Brain	3	5.00	15.00	
	kidney	3	2.00	6.00	
	总数
(Total)	6			
Ncam1	Brain	3	5.00	15.00	
	kidney	3	2.00	6.00	
	总数
(Total)	6			
Ncam2	Brain	3	5.00	15.00	
	kidney	3	2.00	6.00	
	总数
(Total)	6			
Neurod1	Brain	3	5.00	15.00	
	kidney	3	2.00	6.00	
	总数
(Total)	6			
Mapt	Brain	3	5.00	15.00	
	kidney	3	2.00	6.00	
	总数
(Total)	6			
Calb1	Brain	3	3.00	9.00	
	kidney	3	4.00	12.00	
	总数
(Total)	6			
Calb2	Brain	3	5.00	15.00	
	kidney	3	2.00	6.00	
	总数
(Total)	6			
Nefh	Brain	3	5.00	15.00	
	kidney	3	2.00	6.00	
	总数
(Total)	6			
Nefl	Brain	3	5.00	15.00	
	kidney	3	2.00	6.00	
	总数
(Total)	6			
Nefm	Brain	3	5.00	15.00	
	kidney	3	2.00	6.00	
	总数
(Total)	6			
Gfap	Brain	3	5.00	15.00	
	kidney	3	2.00	6.00	
	总数
(Total)	6			
S100b	Brain	3	5.00	15.00	
	kidney	3	2.00	6.00	
	总数
(Total)	6			
Vim	Brain	3	5.00	15.00	
	kidney	3	2.00	6.00	
	总数
(Total)	6			
Cnp	Brain	3	5.00	15.00	
	kidney	3	2.00	6.00	
	总数
(Total)	6			


检验统计¶qb(Test statistics)	
	Eno2	Rbfox3	Map2	Tubb3	Dcx	Chat	
Mann-Whitney U	.000	.000	.000	.000	.000	.000	
Wilcoxon W	6.000	6.000	6.000	6.000	6.000	6.000	
Z	-1.964	-1.964	-1.964	-1.964	-1.964	-2.087	
渐ªñ显µÛ©Ê(双侧) Asymptotic significance (bilateral)	.050	.050	.050	.050	.050	.037	
ºëÚÌ显µÛ©Ê[2*¡]单侧显µÛ©Ê¡^]
Precise significance [2* (unilateral significance)]	.100a	.100a	.100a	.100a	.100a	.100a	
a. 没¦³对结进¦æ­×¥¿¡C(No modifications were made)	
b. ¤À组变¶q(Grouping variable): group	

检验统计¶qb(Test statistics)	
	Th	Ncam1	Ncam2	Neurod1	Mapt	Calb1	
Mann-Whitney U	.000	.000	.000	.000	.000	3.000	
Wilcoxon W	6.000	6.000	6.000	6.000	6.000	9.000	
Z	-1.993	-1.964	-2.087	-1.993	-1.964	-.655	
渐ªñ显µÛ©Ê(双侧) Asymptotic significance (bilateral)	.046	.050	.037	.046	.050	.513	
ºëÚÌ显µÛ©Ê[2*¡]单侧显µÛ©Ê¡^]
Precise significance [2* (unilateral significance)]	.100a	.100a	.100a	.100a	.100a	.700a	
a. 没¦³对结进¦æ­×¥¿¡C(No modifications were made)	
b. ¤À组变¶q(Grouping variable): group	

检验统计¶qb(Test statistics)	
	Calb2	Nefh	Nefl	Nefm	Gfap	S100b	
Mann-Whitney U	.000	.000	.000	.000	.000	.000	
Wilcoxon W	6.000	6.000	6.000	6.000	6.000	6.000	
Z	-2.087	-1.964	-1.964	-1.964	-1.964	-1.964	
渐ªñ显µÛ©Ê(双侧) Asymptotic significance (bilateral)	.037	.050	.050	.050	.050	.050	
ºëÚÌ显µÛ©Ê[2*¡]单侧显µÛ©Ê¡^]
Precise significance [2* (unilateral significance)]	.100a	.100a	.100a	.100a	.100a	.100a	
a. 没¦³对结进¦æ­×¥¿¡C(No modifications were made)	
b. ¤À组变¶q(Grouping variable): group	

检验统计¶qb(Test statistics)	
	Vim	Cnp	
Mann-Whitney U	.000	.000	
Wilcoxon W	6.000	6.000	
Z	-1.964	-1.964	
渐ªñ显µÛ©Ê(双侧) Asymptotic significance (bilateral)	.050	.050	
ºëÚÌ显µÛ©Ê[2*¡]单侧显µÛ©Ê¡^]
Precise significance [2* (unilateral significance)]	.100a	.100a	
a. 没¦³对结进¦æ­×¥¿¡C(No modifications were made)	
b. ¤À组变¶q(Grouping variable): group	


Stomach vs Brain

NPAR TESTS
  /M-W= Eno2 Rbfox3 Map2 Tubb3 Dcx Chat Th Ncam1 Ncam2 Neurod1 Mapt Calb1 Calb2 Nefh Nefl Nefm Gfap S100b Vim Cnp BY group(1 6)
  /MISSING ANALYSIS.

NPar 检验 (NPar Test)

ªþª`(Note)	
创«Øªº输¥X(Output)	14-¤T¤ë-2019 08时09¤À52¬í	
ª`释(Note)	 (14 Mar., 2019  08:09:52)	
输¤J(Input)	数Õu(Data)	F:\¤¤医学°|\NSFC\Uricase\转录组测§Ç\´²¸¨ªº¯«经¤¸\论¤å\Cell Bio Int\Raw data for uploading\analysis.sav	
	¬¡动ªº数Õu¶°(Active data set)	数Õu¶°1(Data set 1)	
	过滤¾¹(Filter)	<none>	
	权­«(Weight)	<none>	
	©î¤À¤å¥ó(Split files)	<none>	
	¤u§@数Õu¤å¥ó¤¤ªº N ¦æ(Lines)	24	
¯Ê¥¢­È处²z
(Missing value)	¯Ê¥¢©w义(Definition)	¥Î户©w义ªº¯Ê¥¢­È¥H¯Ê¥¢对«Ý¡C(According to the default)	
	¨Ï¥Îªº®×¨Ò (Cases)	¨C个检验ªº统计¶q³£¬O°ò¤_对¤_该检验©Ò¨Ï¥Îªº变¶q³£¨ã¦³¨ä¦³®Ä数Õuªº©Ò¦³®×¨Ò¡C(Based on all the cases)	
语ªk(Syntax)	NPAR TESTS
  /M-W= Eno2 Rbfox3 Map2 Tubb3 Dcx Chat Th Ncam1 Ncam2 Neurod1 Mapt Calb1 Calb2 Nefh Nefl Nefm Gfap S100b Vim Cnp BY group(1 6)
  /MISSING ANALYSIS.
	
资·½(Resource)	处²z¾¹时间 (Processor time)	00:00:00.000	
	¤w¥Î时间 (Elapsed time)	00:00:00.010	
	¤¹许ªº®×¨Ò数a
(Cases permissible)	30247	
a. °ò¤_¤u§@ªÅ间内¦sªº¥i¥Î©Ê¡C(Cases allowed for analysis based on the RAM)	


[数Õu¶°1] (Data set 1)  F:\¤¤医学°|\NSFC\Uricase\转录组测§Ç\´²¸¨ªº¯«经¤¸\论¤å\Cell Bio Int\Raw data for uploading\analysis.sav


Mann-Whitney 检验 (Mann-Whitney Test)


¯´(Rank)	
	group	N	¯´§¡­È(Mean of rank)	¯´©M (Sum of rank)	
Eno2	Brain	3	5.00	15.00	
	stomach	3	2.00	6.00	
	总数
(Total)	6			
Rbfox3	Brain	3	5.00	15.00	
	stomach	3	2.00	6.00	
	总数
(Total)	6			
Map2	Brain	3	5.00	15.00	
	stomach	3	2.00	6.00	
	总数
(Total)	6			
Tubb3	Brain	3	5.00	15.00	
	stomach	3	2.00	6.00	
	总数
(Total)	6			
Dcx	Brain	3	5.00	15.00	
	stomach	3	2.00	6.00	
	总数
(Total)	6			
Chat	Brain	3	5.00	15.00	
	stomach	3	2.00	6.00	
	总数
(Total)	6			
Th	Brain	3	5.00	15.00	
	stomach	3	2.00	6.00	
	总数
(Total)	6			
Ncam1	Brain	3	5.00	15.00	
	stomach	3	2.00	6.00	
	总数
(Total)	6			
Ncam2	Brain	3	5.00	15.00	
	stomach	3	2.00	6.00	
	总数
(Total)	6			
Neurod1	Brain	3	5.00	15.00	
	stomach	3	2.00	6.00	
	总数
(Total)	6			
Mapt	Brain	3	5.00	15.00	
	stomach	3	2.00	6.00	
	总数
(Total)	6			
Calb1	Brain	3	5.00	15.00	
	stomach	3	2.00	6.00	
	总数
(Total)	6			
Calb2	Brain	3	5.00	15.00	
	stomach	3	2.00	6.00	
	总数
(Total)	6			
Nefh	Brain	3	5.00	15.00	
	stomach	3	2.00	6.00	
	总数
(Total)	6			
Nefl	Brain	3	5.00	15.00	
	stomach	3	2.00	6.00	
	总数
(Total)	6			
Nefm	Brain	3	5.00	15.00	
	stomach	3	2.00	6.00	
	总数
(Total)	6			
Gfap	Brain	3	5.00	15.00	
	stomach	3	2.00	6.00	
	总数
(Total)	6			
S100b	Brain	3	5.00	15.00	
	stomach	3	2.00	6.00	
	总数
(Total)	6			
Vim	Brain	3	2.00	6.00	
	stomach	3	5.00	15.00	
	总数
(Total)	6			
Cnp	Brain	3	5.00	15.00	
	stomach	3	2.00	6.00	
	总数
(Total)	6			


检验统计¶qb(Test statistics)	
	Eno2	Rbfox3	Map2	Tubb3	Dcx	Chat	
Mann-Whitney U	.000	.000	.000	.000	.000	.000	
Wilcoxon W	6.000	6.000	6.000	6.000	6.000	6.000	
Z	-1.964	-1.964	-1.964	-1.964	-1.993	-1.993	
渐ªñ显µÛ©Ê(双侧) Asymptotic significance (bilateral)	.050	.050	.050	.050	.046	.046	
ºëÚÌ显µÛ©Ê[2*¡]单侧显µÛ©Ê¡^]
Precise significance [2* (unilateral significance)]	.100a	.100a	.100a	.100a	.100a	.100a	
a. 没¦³对结进¦æ­×¥¿¡C(No modifications were made)	
b. ¤À组变¶q(Grouping variable): group	

检验统计¶qb(Test statistics)	
	Th	Ncam1	Ncam2	Neurod1	Mapt	Calb1	
Mann-Whitney U	.000	.000	.000	.000	.000	.000	
Wilcoxon W	6.000	6.000	6.000	6.000	6.000	6.000	
Z	-1.993	-1.964	-1.964	-1.964	-1.964	-1.964	
渐ªñ显µÛ©Ê(双侧) Asymptotic significance (bilateral)	.046	.050	.050	.050	.050	.050	
ºëÚÌ显µÛ©Ê[2*¡]单侧显µÛ©Ê¡^]
Precise significance [2* (unilateral significance)]	.100a	.100a	.100a	.100a	.100a	.100a	
a. 没¦³对结进¦æ­×¥¿¡C(No modifications were made)	
b. ¤À组变¶q(Grouping variable): group	

检验统计¶qb(Test statistics)	
	Calb2	Nefh	Nefl	Nefm	Gfap	S100b	
Mann-Whitney U	.000	.000	.000	.000	.000	.000	
Wilcoxon W	6.000	6.000	6.000	6.000	6.000	6.000	
Z	-1.964	-1.964	-1.964	-1.993	-1.964	-1.964	
渐ªñ显µÛ©Ê(双侧) Asymptotic significance (bilateral)	.050	.050	.050	.046	.050	.050	
ºëÚÌ显µÛ©Ê[2*¡]单侧显µÛ©Ê¡^]
Precise significance [2* (unilateral significance)]	.100a	.100a	.100a	.100a	.100a	.100a	
a. 没¦³对结进¦æ­×¥¿¡C(No modifications were made)	
b. ¤À组变¶q(Grouping variable): group	

检验统计¶qb(Test statistics)	
	Vim	Cnp	
Mann-Whitney U	.000	.000	
Wilcoxon W	6.000	6.000	
Z	-1.964	-1.964	
渐ªñ显µÛ©Ê(双侧) Asymptotic significance (bilateral)	.050	.050	
ºëÚÌ显µÛ©Ê[2*¡]单侧显µÛ©Ê¡^]
Precise significance [2* (unilateral significance)]	.100a	.100a	
a. 没¦³对结进¦æ­×¥¿¡C(No modifications were made)	
b. ¤À组变¶q(Grouping variable): group	


Duodenum vs Brain

NPAR TESTS
  /M-W= Eno2 Rbfox3 Map2 Tubb3 Dcx Chat Th Ncam1 Ncam2 Neurod1 Mapt Calb1 Calb2 Nefh Nefl Nefm Gfap S100b Vim Cnp BY group(1 7)
  /MISSING ANALYSIS.

NPar 检验 (NPar Test)

ªþª`(Note)	
创«Øªº输¥X(Output)	14-¤T¤ë-2019 08时10¤À49¬í	
ª`释(Note)	 (14 Mar., 2019  08:10:49)	
输¤J(Input)	数Õu(Data)	F:\¤¤医学°|\NSFC\Uricase\转录组测§Ç\´²¸¨ªº¯«经¤¸\论¤å\Cell Bio Int\Raw data for uploading\analysis.sav	
	¬¡动ªº数Õu¶°(Active data set)	数Õu¶°1(Data set 1)	
	过滤¾¹(Filter)	<none>	
	权­«(Weight)	<none>	
	©î¤À¤å¥ó(Split files)	<none>	
	¤u§@数Õu¤å¥ó¤¤ªº N ¦æ(Lines)	24	
¯Ê¥¢­È处²z
(Missing value)	¯Ê¥¢©w义(Definition)	¥Î户©w义ªº¯Ê¥¢­È¥H¯Ê¥¢对«Ý¡C(According to the default)	
	¨Ï¥Îªº®×¨Ò (Cases)	¨C个检验ªº统计¶q³£¬O°ò¤_对¤_该检验©Ò¨Ï¥Îªº变¶q³£¨ã¦³¨ä¦³®Ä数Õuªº©Ò¦³®×¨Ò¡C(Based on all the cases)	
语ªk(Syntax)	NPAR TESTS
  /M-W= Eno2 Rbfox3 Map2 Tubb3 Dcx Chat Th Ncam1 Ncam2 Neurod1 Mapt Calb1 Calb2 Nefh Nefl Nefm Gfap S100b Vim Cnp BY group(1 7)
  /MISSING ANALYSIS.
	
资·½(Resource)	处²z¾¹时间 (Processor time)	00:00:00.000	
	¤w¥Î时间 (Elapsed time)	00:00:00.000	
	¤¹许ªº®×¨Ò数a
(Cases permissible)	30247	
a. °ò¤_¤u§@ªÅ间内¦sªº¥i¥Î©Ê¡C(Cases allowed for analysis based on the RAM)	


[数Õu¶°1] (Data set 1)  F:\¤¤医学°|\NSFC\Uricase\转录组测§Ç\´²¸¨ªº¯«经¤¸\论¤å\Cell Bio Int\Raw data for uploading\analysis.sav


Mann-Whitney 检验 (Mann-Whitney Test)


¯´(Rank)	
	group	N	¯´§¡­È(Mean of rank)	¯´©M (Sum of rank)	
Eno2	Brain	3	5.00	15.00	
	duodenum	3	2.00	6.00	
	总数
(Total)	6			
Rbfox3	Brain	3	5.00	15.00	
	duodenum	3	2.00	6.00	
	总数
(Total)	6			
Map2	Brain	3	5.00	15.00	
	duodenum	3	2.00	6.00	
	总数
(Total)	6			
Tubb3	Brain	3	5.00	15.00	
	duodenum	3	2.00	6.00	
	总数
(Total)	6			
Dcx	Brain	3	5.00	15.00	
	duodenum	3	2.00	6.00	
	总数
(Total)	6			
Chat	Brain	3	4.33	13.00	
	duodenum	3	2.67	8.00	
	总数
(Total)	6			
Th	Brain	3	5.00	15.00	
	duodenum	3	2.00	6.00	
	总数
(Total)	6			
Ncam1	Brain	3	5.00	15.00	
	duodenum	3	2.00	6.00	
	总数
(Total)	6			
Ncam2	Brain	3	5.00	15.00	
	duodenum	3	2.00	6.00	
	总数
(Total)	6			
Neurod1	Brain	3	5.00	15.00	
	duodenum	3	2.00	6.00	
	总数
(Total)	6			
Mapt	Brain	3	5.00	15.00	
	duodenum	3	2.00	6.00	
	总数
(Total)	6			
Calb1	Brain	3	5.00	15.00	
	duodenum	3	2.00	6.00	
	总数
(Total)	6			
Calb2	Brain	3	5.00	15.00	
	duodenum	3	2.00	6.00	
	总数
(Total)	6			
Nefh	Brain	3	5.00	15.00	
	duodenum	3	2.00	6.00	
	总数
(Total)	6			
Nefl	Brain	3	5.00	15.00	
	duodenum	3	2.00	6.00	
	总数
(Total)	6			
Nefm	Brain	3	5.00	15.00	
	duodenum	3	2.00	6.00	
	总数
(Total)	6			
Gfap	Brain	3	5.00	15.00	
	duodenum	3	2.00	6.00	
	总数
(Total)	6			
S100b	Brain	3	5.00	15.00	
	duodenum	3	2.00	6.00	
	总数
(Total)	6			
Vim	Brain	3	4.67	14.00	
	duodenum	3	2.33	7.00	
	总数
(Total)	6			
Cnp	Brain	3	5.00	15.00	
	duodenum	3	2.00	6.00	
	总数
(Total)	6			


检验统计¶qb(Test statistics)	
	Eno2	Rbfox3	Map2	Tubb3	Dcx	Chat	
Mann-Whitney U	.000	.000	.000	.000	.000	2.000	
Wilcoxon W	6.000	6.000	6.000	6.000	6.000	8.000	
Z	-1.964	-1.964	-1.964	-1.964	-1.993	-1.091	
渐ªñ显µÛ©Ê(双侧) Asymptotic significance (bilateral)	.050	.050	.050	.050	.046	.275	
ºëÚÌ显µÛ©Ê[2*¡]单侧显µÛ©Ê¡^]
Precise significance [2* (unilateral significance)]	.100a	.100a	.100a	.100a	.100a	.400a	
a. 没¦³对结进¦æ­×¥¿¡C(No modifications were made)	
b. ¤À组变¶q(Grouping variable): group	

检验统计¶qb(Test statistics)	
	Th	Ncam1	Ncam2	Neurod1	Mapt	Calb1	
Mann-Whitney U	.000	.000	.000	.000	.000	.000	
Wilcoxon W	6.000	6.000	6.000	6.000	6.000	6.000	
Z	-1.993	-1.964	-1.964	-1.964	-1.964	-1.964	
渐ªñ显µÛ©Ê(双侧) Asymptotic significance (bilateral)	.046	.050	.050	.050	.050	.050	
ºëÚÌ显µÛ©Ê[2*¡]单侧显µÛ©Ê¡^]
Precise significance [2* (unilateral significance)]	.100a	.100a	.100a	.100a	.100a	.100a	
a. 没¦³对结进¦æ­×¥¿¡C(No modifications were made)	
b. ¤À组变¶q(Grouping variable): group	

检验统计¶qb(Test statistics)	
	Calb2	Nefh	Nefl	Nefm	Gfap	S100b	
Mann-Whitney U	.000	.000	.000	.000	.000	.000	
Wilcoxon W	6.000	6.000	6.000	6.000	6.000	6.000	
Z	-1.964	-1.964	-1.964	-1.964	-1.964	-1.964	
渐ªñ显µÛ©Ê(双侧) Asymptotic significance (bilateral)	.050	.050	.050	.050	.050	.050	
ºëÚÌ显µÛ©Ê[2*¡]单侧显µÛ©Ê¡^]
Precise significance [2* (unilateral significance)]	.100a	.100a	.100a	.100a	.100a	.100a	
a. 没¦³对结进¦æ­×¥¿¡C(No modifications were made)	
b. ¤À组变¶q(Grouping variable): group	

检验统计¶qb(Test statistics)	
	Vim	Cnp	
Mann-Whitney U	1.000	.000	
Wilcoxon W	7.000	6.000	
Z	-1.528	-1.964	
渐ªñ显µÛ©Ê(双侧) Asymptotic significance (bilateral)	.127	.050	
ºëÚÌ显µÛ©Ê[2*¡]单侧显µÛ©Ê¡^]
Precise significance [2* (unilateral significance)]	.200a	.100a	
a. 没¦³对结进¦æ­×¥¿¡C(No modifications were made)	
b. ¤À组变¶q(Grouping variable): group	


Ileum vs Brain

NPAR TESTS
  /M-W= Eno2 Rbfox3 Map2 Tubb3 Dcx Chat Th Ncam1 Ncam2 Neurod1 Mapt Calb1 Calb2 Nefh Nefl Nefm Gfap S100b Vim Cnp BY group(1 8)
  /MISSING ANALYSIS.

NPar 检验 (NPar Test)

ªþª`(Note)	
创«Øªº输¥X(Output)	14-¤T¤ë-2019 08时11¤À15¬í	
ª`释(Note)	 (14 Mar., 2019  08:11:15)	
输¤J(Input)	数Õu(Data)	F:\¤¤医学°|\NSFC\Uricase\转录组测§Ç\´²¸¨ªº¯«经¤¸\论¤å\Cell Bio Int\Raw data for uploading\analysis.sav	
	¬¡动ªº数Õu¶°(Active data set)	数Õu¶°1(Data set 1)	
	过滤¾¹(Filter)	<none>	
	权­«(Weight)	<none>	
	©î¤À¤å¥ó(Split files)	<none>	
	¤u§@数Õu¤å¥ó¤¤ªº N ¦æ(Lines)	24	
¯Ê¥¢­È处²z
(Missing value)	¯Ê¥¢©w义(Definition)	¥Î户©w义ªº¯Ê¥¢­È¥H¯Ê¥¢对«Ý¡C(According to the default)	
	¨Ï¥Îªº®×¨Ò (Cases)	¨C个检验ªº统计¶q³£¬O°ò¤_对¤_该检验©Ò¨Ï¥Îªº变¶q³£¨ã¦³¨ä¦³®Ä数Õuªº©Ò¦³®×¨Ò¡C(Based on all the cases)	
语ªk(Syntax)	NPAR TESTS
  /M-W= Eno2 Rbfox3 Map2 Tubb3 Dcx Chat Th Ncam1 Ncam2 Neurod1 Mapt Calb1 Calb2 Nefh Nefl Nefm Gfap S100b Vim Cnp BY group(1 8)
  /MISSING ANALYSIS.
	
资·½(Resource)	处²z¾¹时间 (Processor time)	00:00:00.015	
	¤w¥Î时间 (Elapsed time)	00:00:00.010	
	¤¹许ªº®×¨Ò数a
(Cases permissible)	30247	
a. °ò¤_¤u§@ªÅ间内¦sªº¥i¥Î©Ê¡C(Cases allowed for analysis based on the RAM)	


[数Õu¶°1] (Data set 1)  F:\¤¤医学°|\NSFC\Uricase\转录组测§Ç\´²¸¨ªº¯«经¤¸\论¤å\Cell Bio Int\Raw data for uploading\analysis.sav


Mann-Whitney 检验 (Mann-Whitney Test)

	¯´(Rank)	
		group	N	¯´§¡­È(Mean of rank)	¯´©M (Sum of rank)	
Eno2	dimension1	Brain	3	5.00	15.00	
		ileum	3	2.00	6.00	
		总数
(Total)	6			
Rbfox3	dimension1	Brain	3	5.00	15.00	
		ileum	3	2.00	6.00	
		总数
(Total)	6			
Map2	dimension1	Brain	3	5.00	15.00	
		ileum	3	2.00	6.00	
		总数
(Total)	6			
Tubb3	dimension1	Brain	3	5.00	15.00	
		ileum	3	2.00	6.00	
		总数
(Total)	6			
Dcx	dimension1	Brain	3	5.00	15.00	
		ileum	3	2.00	6.00	
		总数
(Total)	6			
Chat	dimension1	Brain	3	4.00	12.00	
		ileum	3	3.00	9.00	
		总数
(Total)	6			
Th	dimension1	Brain	3	5.00	15.00	
		ileum	3	2.00	6.00	
		总数
(Total)	6			
Ncam1	dimension1	Brain	3	5.00	15.00	
		ileum	3	2.00	6.00	
		总数
(Total)	6			
Ncam2	dimension1	Brain	3	5.00	15.00	
		ileum	3	2.00	6.00	
		总数
(Total)	6			
Neurod1	dimension1	Brain	3	5.00	15.00	
		ileum	3	2.00	6.00	
		总数
(Total)	6			
Mapt	dimension1	Brain	3	5.00	15.00	
		ileum	3	2.00	6.00	
		总数
(Total)	6			
Calb1	dimension1	Brain	3	5.00	15.00	
		ileum	3	2.00	6.00	
		总数
(Total)	6			
Calb2	dimension1	Brain	3	5.00	15.00	
		ileum	3	2.00	6.00	
		总数
(Total)	6			
Nefh	dimension1	Brain	3	5.00	15.00	
		ileum	3	2.00	6.00	
		总数
(Total)	6			
Nefl	dimension1	Brain	3	5.00	15.00	
		ileum	3	2.00	6.00	
		总数
(Total)	6			
Nefm	dimension1	Brain	3	5.00	15.00	
		ileum	3	2.00	6.00	
		总数
(Total)	6			
Gfap	dimension1	Brain	3	5.00	15.00	
		ileum	3	2.00	6.00	
		总数
(Total)	6			
S100b	dimension1	Brain	3	5.00	15.00	
		ileum	3	2.00	6.00	
		总数
(Total)	6			
Vim	dimension1	Brain	3	2.00	6.00	
		ileum	3	5.00	15.00	
		总数
(Total)	6			
Cnp	dimension1	Brain	3	5.00	15.00	
		ileum	3	2.00	6.00	
		总数
(Total)	6			


检验统计¶qb(Test statistics)	
	Eno2	Rbfox3	Map2	Tubb3	Dcx	Chat	
Mann-Whitney U	.000	.000	.000	.000	.000	3.000	
Wilcoxon W	6.000	6.000	6.000	6.000	6.000	9.000	
Z	-1.964	-1.964	-1.964	-1.964	-1.964	-.655	
渐ªñ显µÛ©Ê(双侧) Asymptotic significance (bilateral)	.050	.050	.050	.050	.050	.513	
ºëÚÌ显µÛ©Ê[2*¡]单侧显µÛ©Ê¡^]
Precise significance [2* (unilateral significance)]	.100a	.100a	.100a	.100a	.100a	.700a	
a. 没¦³对结进¦æ­×¥¿¡C(No modifications were made)	
b. ¤À组变¶q(Grouping variable): group	

检验统计¶qb(Test statistics)	
	Th	Ncam1	Ncam2	Neurod1	Mapt	Calb1	
Mann-Whitney U	.000	.000	.000	.000	.000	.000	
Wilcoxon W	6.000	6.000	6.000	6.000	6.000	6.000	
Z	-1.964	-1.964	-1.964	-1.964	-1.964	-1.964	
渐ªñ显µÛ©Ê(双侧) Asymptotic significance (bilateral)	.050	.050	.050	.050	.050	.050	
ºëÚÌ显µÛ©Ê[2*¡]单侧显µÛ©Ê¡^]
Precise significance [2* (unilateral significance)]	.100a	.100a	.100a	.100a	.100a	.100a	
a. 没¦³对结进¦æ­×¥¿¡C(No modifications were made)	
b. ¤À组变¶q(Grouping variable): group	

检验统计¶qb(Test statistics)	
	Calb2	Nefh	Nefl	Nefm	Gfap	S100b	
Mann-Whitney U	.000	.000	.000	.000	.000	.000	
Wilcoxon W	6.000	6.000	6.000	6.000	6.000	6.000	
Z	-1.964	-1.964	-1.964	-1.964	-1.964	-1.964	
渐ªñ显µÛ©Ê(双侧) Asymptotic significance (bilateral)	.050	.050	.050	.050	.050	.050	
ºëÚÌ显µÛ©Ê[2*¡]单侧显µÛ©Ê¡^]
Precise significance [2* (unilateral significance)]	.100a	.100a	.100a	.100a	.100a	.100a	
a. 没¦³对结进¦æ­×¥¿¡C(No modifications were made)	
b. ¤À组变¶q(Grouping variable): group	

检验统计¶qb(Test statistics)	
	Vim	Cnp	
Mann-Whitney U	.000	.000	
Wilcoxon W	6.000	6.000	
Z	-1.964	-1.964	
渐ªñ显µÛ©Ê(双侧) Asymptotic significance (bilateral)	.050	.050	
ºëÚÌ显µÛ©Ê[2*¡]单侧显µÛ©Ê¡^]
Precise significance [2* (unilateral significance)]	.100a	.100a	
a. 没¦³对结进¦æ­×¥¿¡C(No modifications were made)	
b. ¤À组变¶q: group	
